# Clearance of senescent cells reverts the cigarette smoke‐induced lung senescence and airspace enlargement in p16‐3MR mice

**DOI:** 10.1111/acel.13850

**Published:** 2023-04-20

**Authors:** Gagandeep Kaur, Thivanka Muthumalage, Irfan Rahman

**Affiliations:** ^1^ Department of Environmental Medicine University of Rochester Medical Center Rochester New York USA

**Keywords:** cellular senescence, COPD, immunosenescence, p16‐3MR

## Abstract

Cigarette smoke (CS) leads to increased oxidative stress, inflammation, and exaggerated senescence, which are involved in the pathogenesis of chronic obstructive pulmonary disease (COPD). While the role of cellular senescence in COPD is known, it is not clear if the removal of senescent cells could alleviate the disease symptoms. To test this, we used the novel mouse model‐p16‐3MR, and studied the effect of ganciclovir (GCV)‐mediated removal of senescent cells after chronic CS (3 months) and environmental tobacco smoke (ETS) (6 months) exposure to CS. Our results showed the reversal of CS‐induced cellular senescence on the clearance of p16^+^ senesced cells by GCV treatment. Interestingly, the clearance of p16^+^ senescent cells via GCV led to a decrease in the neutrophil counts in the BALF of GCV‐treated CS‐exposed p16‐3MR mice, as well as reversal of CS‐mediated airspace enlargement in p16‐3MR mice. Mice exposed to low dose ETS caused insignificant changes in the SA‐β‐Gal^+^ senescent cells and airspace enlargement. Overall, our data provide evidence for the role of lung cellular senescence on smoke exposure and clearance of senescent cells in p16‐3MR mice in the reversal of COPD/emphysema pathology with a possibility of senolytics as therapeutic interventions in COPD.

## Graphical abstract

Using a novel mouse model‐p16‐3MR, we showed the reversal of cigarette smoke (CS)‐induced cellular senescence on the clearance of p16^+^ senesced cells by ganciclovir treatment as well as reversal of CS‐mediated airspace enlargement. We provide the evidence on the role of lung cellular senescence on smoke exposure and clearance of senescent cells in the reversal of chronic obstructive pulmonary disease (COPD)/emphysema pathology with a possibility of senolytics as therapeutic interventions in COPD.

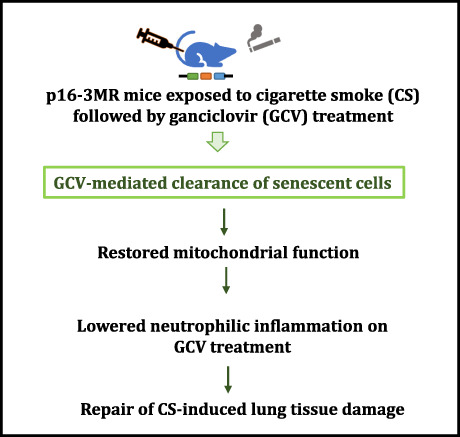

**Abbreviations**
BALF: bronchoalveolar lavage fluid
COPD: chronic obstructive pulmonary disease
CS: cigarette smoke
ETS: environmental tobacco smoke
GCV: ganciclovir
IHC: immunohistochemistry
IPF: idiopathic pulmonary fibrosis
OCT: optimal cutting temperature
PBS: phosphate buffer saline
PFA: paraformaldehyde
TPM: total particulate matter

## 1 INTRODUCTION

Accelerated premature ageing due to increased cellular senescence has been proposed in various lung diseases including chronic obstructive pulmonary disease (COPD) and idiopathic pulmonary fibrosis (IPF) (Barnes et al., 2019). While the role of cellular senescence in IPF has been studied and there is evidence that targeting senescent cells could help improve the clinical outcomes in IPF, the role of cellular senescence in the development and progress of COPD is still unknown (Kellogg et al., 2021; Mercado et al., 2015; Schafer et al., 2017). It is known that cigarette smoke (CS), one of the risk factors for the development of COPD, causes oxidative stress and DNA damage that ultimately leads to increased cellular senescence (Cottage et al., 2019; Nyunoya et al., 2006). However, there is little to no evidence with regards to the therapeutic relevance of elimination of senescent cells in reversing the lung damage and airway enlargement caused due to CS in the pathogenesis of COPD.

In this regard, tumor suppressors p19^ARF^ and p16^INK4a^, are considered to play critical roles as they function to induce and maintain permanent cell cycle arrest. Research suggests induction of p16 expression on exposure to CS (Cottage et al., 2019; Paramos‐de‐Carvalho et al., 2021). However, its role in the induction of COPD is not clear. We thus employed the novel transgenic mouse model p16‐3MR to study CS‐induced cellular senescence. This mouse model allows the identification, isolation and selective elimination of p16‐expressing senescent cells, thus making it a powerful tool to study the physiological role of cellular senescence in various pathologies (Demaria et al., 2014; Kaur et al., 2021). p16‐3MR is a trimodality reporter mice with functional domains of synthetic Renilla luciferase (LUC), monomeric red fluorescent protein (mRFP), and truncated herpes simplex virus 1 (HSV‐1) thymidine kinase (HSV‐TK). Previous work by our group studied the suitability of using p16‐3MR as a mouse model for studying cellular senescence on CS exposure (Kaur et al., 2021). However, we did not test the effectiveness of elimination of senescent cells in reversing the CS‐induced lung inflammation or tissue damage (the common hallmarks of COPD) in that study. Here, we further our studies and expose p16‐3MR mice to CS for chronic 3 months' duration to induce CS‐related cellular senescence followed by 5‐day administration of ganciclovir (GCV) to test the effect of elimination of p16‐expressing senescent cells on the CS‐induced lung cellular senescence. To test if passive exposure to CS has similar effects, we evaluated senescence induction after chronic exposure to low levels of environmental tobacco smoke (ETS) exposure for 6 months' duration in p16‐3MR mice.

COPD/emphysema is a common disease associated with aging lung. Literature suggests role of accelerated cellular senescence in COPD and speculates that senolytics/senomorphics could be a good therapeutic intervention for the disease, but there is little evidence that proves this hypothesis. Overall, current study attempts to fill this gap in knowledge by using the transgenic mouse model, p16‐3MR, to show restored mitochondrial function, lowered neutrophil influx and alteration in tissue damage mechanisms on clearance of CS‐induced senescent cells on GCV‐mediated removal of p16^+^ senescent cells. We thus provide proof that clearance of senescent cells has therapeutic potential in COPD/emphysema.

## 2 MATERIALS AND METHODS

### 2.1 Ethics statement: Institutional biosafety and IACUC/UCAR animal experiment approval

Experiments in this study were performed according to the standards and guidelines approved by The University of Rochester Institutional Biosafety Committee. All mouse housing, handling, exposure, and procedure protocols used in this study were approved by the University Committee on Animal Research (UCAR) Committee of the University of Rochester, Rochester, NY. Great care was taken to employ a robust and unbiased approach during the experimental and corresponding results analysis phase in order to ensure reproducibility befitting NIH standards.

### 2.2 Animal

We employed adult (4–12 months; both male and female) p16‐3MR mice obtained from Dr. Judith Campisi of the Buck Institute for Research on Aging to conduct our experiments as described previously (Demaria et al., 2014). For ETS exposure, 6‐ to 12‐month‐old mice were used. Whereas for CS exposure, 4‐ to 12‐month‐old p16‐3MR mice were employed.

p16‐3MR mice are diploid for p16^INK4a^ and p19^Arf^ with a trimodal (3MR) reporter fusion protein consisting of LUC (for identification), mRFP (for isolation) and HSV‐TK (for selective elimination of p16^+^ senescent cells on GCV treatment) domains. All the mice were housed in the vivarium facility at the University of Rochester Medical Center with a 12‐h light/12‐h dark cycle. All the animals used in the study were genotyped prior to experimentation.

### 2.3 Environmental tobacco smoke exposure

We used research‐grade 3R4F cigarettes (University of Kentucky, Lexington, KY) to generate low levels of ETS by mixing mainstream and sidestream smoke using the Teague smoking machine (Model TE‐10; Teague Enterprises) according to the federal trade commission method (35 cm^3^ puff volume, 2 s puff duration, once a minute) at a concentration of ∼10 mg/m^3^ total particulate matter (TPM) as described earlier (Yao et al., 2014). Whole‐body mouse exposures were performed 5 h/day, 5 days/week for 6 months. Mice not exposed to ETS were considered as the control (Air) group. Animals were sacrificed 24 h after the last ETS exposure.

### 2.4 Cigarette smoke exposure

Male and female mice of different age‐groups (4–12 months) were exposed to 3 months (chronic) CS generated by research grade cigarettes (3R4F) according to the Federal Trade Commission protocol (1 puff/min of 2 s duration and 35‐mL volume for a total of 8 puffs at a flow rate of 1.05 L/min) with a Baumgartner‐Jaeger CSM2072i automatic CS generating machine (CH Technologies) as described previously (Rajendrasozhan et al., 2010; Yao et al., 2008). The CS exposure was performed at a concentration of ~200–250 mg/m^3^ total particulate matter (TPM) by adjusting the flow rate of the diluted air, and the level of carbon monoxide in the chamber was measured at ~320 ppm. Mice not exposed to CS were considered as the control (Air) group. All mice were euthanized 24 h following the final exposure.

### 2.5 Drug dosing

Five days prior to killing the mice, a designated number of mice from the unexposed air, ETS‐exposed, and CS‐exposed groups were treated with vehicle (PBS) or GCV. GCV (Cat# G2536, Millipore Sigma) was administered intraperitoneally (i.p.) daily for five consecutive days at 25 mg/kg body weight in PBS. Control mice were injected with an equal volume of PBS.

### 2.6 Lung mechanics

Lung mechanical properties, including lung compliance, resistance, elastance, tissue damping, and hysteresis, were determined as previously described (Sundar et al., 2018; Yao et al., 2012). In brief, the mouse was weighed, anesthetized using an intraperitoneal injection of pentobarbital (90 mg/kg), and tracheostomized. The trachea was cannulated, and the cannula was connected to a computer‐controlled, small animal ventilator (FlexiVent; Scireq).

### 2.7 Tissue luminescence and fluorescence

We employed IVIS® Spectrum multispectral imaging instrument (Caliper Life Sciences, Inc.) to measure tissue luminescence and fluorescence in unexposed air, ETS‐exposed and CS‐exposed p16‐3MR mice as described earlier (Kaur et al., 2021). In brief, lung tissues harvested from euthanized mice were soaked for 10 min in staining solution containing Xenolight RediJect Coelenterazine h bioluminescent substrate (Cat# 706506, Perkin Elmer). Following a 12‐ to 15‐min incubation, tissues were transferred to a fresh 35‐mm dish and luminescence was measured using IVIS® Spectrum multispectral imaging instrument.

Similarly, IVIS® Spectrum multispectral imaging instrument was also used to measure the lung tissue fluorescence (RFP) in our control and experimental groups at an excitation maximum of 535 nm and emission maximum of 580 nm.

Tissue luminescence and fluorescence in C57BL/6J mice was used as experimental control to subtract any background luminescence or fluorescence to obtain relative values to be plotted.

### 2.8 Hematoxylin and eosin (H&E) staining and morphometric analysis

Non‐lavaged mouse lungs were inflated with OCT (50:50) solution in PBS at a pressure of 25 cm H_2_O and fixed with 4% neutral buffered PFA. Fixed lungs were dehydrated, paraffin‐embedded, and sectioned into 4‐μm sections using a rotary microtome (MICROM International GmbH). Hematoxylin and eosin staining was performed as described earlier (Wang et al., 2021) on the lung midsagittal sections to determine “Mean linear intercept” (Lm) of airspace using MetaMorph software (Molecular Devices). Fifteen randomly selected ×200 fields per slide were photographed in a blinded manner, and six of these images were randomly selected for quantification. MetaMorph software was used to calculate Lm values for each of the samples and an average of the quantified values was plotted. Keyence BZ‐X800 Epifluorescence microscope was used to take full scans of the lung tissue sections and its stitching feature was used to obtain the image.

### 2.9 Cotinine assay

Cotinine levels were measured in the blood serum from unexposed air, ETS‐exposed and CS‐exposed p16‐3MR mice using commercially available Mouse/Rat Cotinine ELISA kit (Cat# CO096D, Calbiotech) as per manufacturer's protocol. In brief, 10 μL of standards, controls and samples were added to a 96‐well plate and mixed with 100 μL of Enzyme conjugate. The plate contents were incubated for 60 min at room temperature. Next, 100 μL of Substrate reagent was added to the plate after washing it thrice with wash buffer. Thereafter, the plate was incubated for 30 min at room temperature in dark and 100 μL of stop solution was added to allow color development. Following color development, the absorbance was read at 450 nm using Cytation 5 Cell imaging Multimode instrument (Biotek Instruments).

### 2.10 Collection of bronchoalveolar lavage fluid (BALF)

To determine the immune cell infiltration in the lungs following ETS/CS‐exposure and GCV treatment, we performed flow cytometry on BALF obtained from control and treated animals. Briefly, mice were weighed and injected with 90 mg/kg body weight pentobarbital (Abbott, IL) and sacrificed by exsanguination. The trachea was cannulated and the lungs were lavaged for a cumulative of three times with 0.6 mL of 0.9% sodium chloride (with 0.5% FBS), as described previously (Rashid et al., 2018; Yao et al., 2010). The lavaged fluid was centrifuged, and the cell‐free supernatant was stored at −80°C for future experiments.

### 2.11 Differential cell counts in BALF by flow cytometry

The cell pellet acquired from the BALF collection was resuspended in 1X PBS and used to perform flow cytometry. The total cell counts in each sample was determined using AO/PI staining using Nexcelom Cellometer K2 instrument (Nexcelom Biosciences). The obtained cells were blocked using anti‐CD16/32 and stained with PerCP/Cy5.5 anti‐CD45.1 (Cat# 110728), PE antiF4/80 (Cat# 123110) (Biolegend), AlexaFlour488 anti‐Ly6B.2 (Cat# NBP2‐13770) (Novus Biologicals), PE‐Cy7 anti‐CD4 (Cat# 25‐0041‐82) and APC anti‐CD8 (Cat# 17‐0081‐82) (eBiosciences) for 20 min at 4°C in dark. After staining, the cells were washed twice with 1X FACS buffer and run on Guava easyCyte 8 instrument (Luminex Corporation).

### 2.12 Assessment of cytokine/chemokine levels using Luminex

The level of proinflammatory mediators in BALF was measured with the help of Bio‐Plex Pro Mouse Cytokine Standard 23‐Plex (Cat# 64209360, Bio‐Rad) as per manufacturer's protocol using FlexMap3D instrument (Luminex Instruments).

### 2.13 SA‐β‐Gal IHC staining

To determine the SA‐β‐Gal positive cells in control and treated samples, we employed Senescence detection kit (Cat# ab65351, Abcam). The SA‐β‐Gal staining solution was prepared as per manufacturer's protocol. Frozen tissue section slides were rinsed with 1X PBS and fixed using the Fixative solution for 5–10 min. Thereafter the staining mix was added to the slides and the slides were incubated overnight at 37°C. The next day, the slides were washed with 1X PBS, dried and mounted using the mounting solution. The senescent cells were stained blue and observed using a Nikon Elipse‐Ni brightfield microscope at 20× magnification. Fifteen random images were captured for each sample and five random images were chosen for quantification using ImageJ (Jensen, 2013).

### 2.14 SA‐β‐Gal activity assay

SA‐β‐Gal activity was measured using a cellular senescence activity assay kit (Cat# ENZ‐KIT129‐0120, Enzo Life sciences) as per the manufacturer's protocol as described earlier (Kaur et al., 2021). Briefly, one lung lobe was homogenized in ice‐cold 1X cell lysis buffer containing complete protease inhibitor cocktail. Tissue homogenate was incubated on ice for 30 min and then centrifuged at 13,000 × ***g*** for 15 min at 4°C. The supernatant was collected and stored until further analyses. The protein quantity in each sample was determined and equal quantity of protein was used for each cell lysate. The cell lysate was mixed with 50 μL of assay buffer and incubated for 3 h at room temperature. Following incubation, 50 μL of the reaction mixture was added to 200 μL of Stop solution, and fluorescence was read using Cytation 5 (Biotek) at 360 nm (Excitation)/465 nm (Emission).

### 2.15 Sudan Black IHC staining

For histochemical detection of lipofuscin as another way of measuring cellular senescence, we used Sudan Black B (SBB) staining. Commercially available SBB staining system (Cat# 380B, Millipore Sigma) was used as per manufacturer's protocol. In brief, frozen lung tissue sections were fixed in glutaraldehyde solution for 5 min at room temperature. The fixed tissue sections were washed with 1X PBS and stained with SBB solution provided with the kit for 8–10 min at room temperature. Next the slides were washed with 50% ethanol solution followed by distilled water and counterstained using Nuclear Fast red. Finally, the slides were washed, air dried and mounted using the mounting solution. The stained sections were observed and captured using a Nikon Elipse‐Ni brightfield microscope at 20× magnification. Ten to fifteen random images were captured for each sample and six random images were chosen for quantification using ImageJ (Jensen, 2013).

### 2.16 Gene expression analyses using NanoString

We used NanoString quantification method to determine the gene expression changes in control and treated samples. In brief, ~40 mg of lung tissue was homogenized and total RNA was extracted using Direct‐zol™ RNA Kits (Cat# R2072, Zymo Research) as per manufacturer's recommendations. Isolated RNA samples were quantified and checked for their purity using NanoDrop spectrophotometer (ND‐1000, NanoDrop Technologies). 100 ng RNA was used for NanoString analysis to determine the expression of senescence, mitochondrial function and epithelial‐mesenchymal transition (EMT) related genes using customized panels (Tables S1 and S2). Normalized expression counts were measured using nCounter SPRINT Profiler (NanoString Technologies) and analyzed by nSolver 4.0 software.

### 2.17 Gene expression analyses using qPCR

For few select targets, we used qPCR to determine the gene expression in our treated and control groups. For this, quantitative PCR (qPCR) was performed using cDNA prepared from the isolated RNA from mouse lungs as described earlier. 1000 ng of RNA was used for cDNA preparation using the iScript advanced cDNA kit for RT‐qPCR (Cat# 1725038; Bio‐Rad Laboratories) per manufacturer's protocol. The prepared cDNA was used to determine the expression of *Cxcl1* (PrimePCR ID: qMmuCED0003898), *Cxcl5* (PrimePCR PreAmp ID: qMmuCED0047657) and *Mmp12* (PrimePCR ID: qMmuCED0050489) using pre‐designed primers from Bio‐Rad. Relative mRNA expression of each gene was determined by using CFX96 Touch Real‐Time PCR Detection System (Bio‐Rad Laboratories) where GAPDH served as endogenous control. Calculated mRNA expression of each target gene by 2^−ΔΔCt^ normalized to housekeeping gene has been reported.

### 2.18 Immunofluorescence

In order to study the neutrophil influx into the mouse lungs on CS exposure, we used immunofluorescence on OCT fixed tissue sections. The freshly cut lung slices were fixed for 10 min in ice‐cold acetone. Thereafter, the slides were washed with 1X TBST (1X TBS with 0.025% Triton X) and blocked with 10% normal goat serum (Cat# 50062Z, Invitrogen) for 30 min. The tissue slides were then probed with Ly6 B2 antibody (Cat# NBP2‐13077, Novus Biologicals). The slides were incubated at 4°C overnight in a 1:200 dilution of Ly6 B2 antibody. The next day, the slides were washed with three changes of 1X TBST for 10 min each and incubated in secondary antibody (Cat# A11006, Invitrogen) for 1 h duration. Thereafter the slides were washed and mounted with ProLong Diamond Antifade Mountant with DAPI (Cat# P36962, Invitrogen). The images were captured Nikon Elipse‐Ni fluorescence microscope at 200× magnification.

### 2.19 Statistical analyses

All statistical calculations were performed using GraphPad Prism 9.0. Data are expressed as mean ± SEM. Pairwise comparisons were done using unpaired *t* test. For multi‐group comparisons, one‐way analysis of variance (ANOVA) with ad‐hoc Tukey's test was employed.

## 3 RESULTS

### 3.1 The p16‐3MR mouse model to study clearance of cellular senescence

Demaria et al. (2014) developed the p16‐3MR trimodality reporter mice to identify, isolate, and selectively kill senescent cells due to the presence of functional domains of synthetic Renilla LUC, mRFP, and truncated HSV‐1 HSV‐TK respectively. This model was developed by inactivating the p16^INK4a^ and adjacent p19^Arf^ genes in bacterial artificial chromosome. It was successfully used to show induction of cellular senescence on irradiation using luminescence and its clearance on GCV treatment (Demaria et al., 2014). This makes it a powerful model to study cellular senescence in other disease states as well.

In this respect, CS has been known to cause increased cellular senescence in mouse models and clinical samples (Cottage et al., 2019; Nyunoya et al., 2006; Woldhuis et al., 2020). However, the exact drivers of CS‐induced senescence are not fully understood. In order to study the role of p16‐driven senescence on tobacco smoke exposure and demonstrating if removal of senescent cells could reverse the smoke‐induced lung damage, we exposed p16‐3MR mice to CS for 3 months and low‐dose ETS for 6 months' duration. Five days prior to killing the animals, the mice were dosed with 25 mg/ kg body weight of GCV to remove the p16‐expressing senescent cells as shown in Figures S1a and S3a. This study design was thus used to ascertain the effects of ETS and CS exposure on cellular senescence and to test if removal of p16^+^ senescent cells using GCV affects the downstream signaling.

### 3.2 Effect of CS and ETS exposure on p16‐3MR mice

We began our studies, by recording the body weight changes during the 5 days of GCV treatment in our mouse model. We observed that the average body weights of CS‐exposed mice were lower than the controls during our CS exposure. However, over the course of 5 days we did not find any changes in the body weight during GCV administration (Figure S1b). Serum cotinine levels were tested in air‐ and smoke‐exposed mice to reflect nicotine exposure. Significant increase in the serum cotinine levels of CS‐exposed mice were observed for both PBS and GCV treated groups thus indicating successful exposure to tobacco smoke in our mouse model (Figure S1c).

**FIGURE 1:**
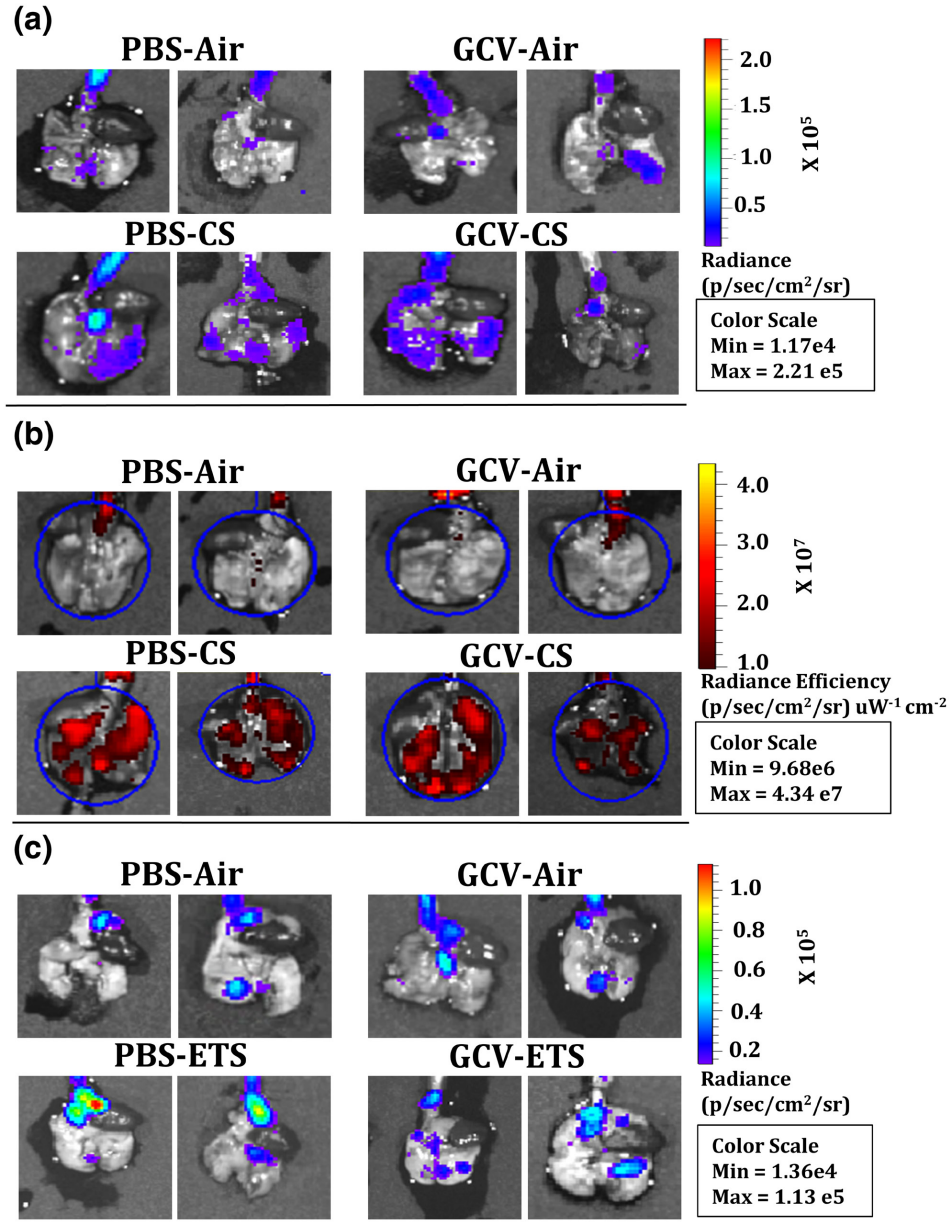
Significant increase in the lung tissue fluorescence of CS‐exposed p16‐3MR mice as compared to air control. p16‐3MR mice were subjected to chronic CS (3 months), followed by 5‐day treatment with GCV (25 mg/kg body weight) or PBS (control). The lung tissues from air‐, and CS‐exposed mice were harvested. Lung tissue (B) luminescence with (b) fluorescence with representative images of *n* = 4–5/group were provided. p16‐3MR mice were subjected to chronic (6 months) ETS exposure, followed by 5‐day treatment with GCV (25 mg/kg body weight) or PBS (control). The lung tissues fluorescence in air and ETS (6 months)‐exposed lungs measured using IVIS imaging. (c) Representative images of *n* = 4–5/group were provided. Lung tissues from air‐exposed C57BL/6J mice were taken as control to measure background fluorescence, if any. SE: *p < 0.05, and **p < 0.01; as per one‐way ANOVA for multiple comparisons.

To verify the 3MR transgene activity on CS exposure, the lung tissue luminescence and fluorescence was visualized using IVIS imaging. While no change was observed in the lung tissue luminescence on CS exposure (Figure 1a and respective relative quantified counts measured using IVIS imaging as PBS‐Air: 3248 ± 1131; PBS‐CS: 4661 ± 1413; GCV‐Air: 3014 ± 799 and GCV‐CS: 4939 ± 2834, data are mean±SEM; *n* = 4‐5/group vs their air groups), overall lung tissue fluorescence increased significantly in CS‐exposed p16‐3MR mice as compared to the air controls which showed induction of p16 gene expression on CS exposure (Figure 1b and respective relative quantified counts measured using IVIS imaging as PBS‐Air: 0.819 ± 0.14; PBS‐CS 1.588 ± 0.31** with 1.94 fold increase in tissue fluorescence; GCV‐Air: 0.841 ± 0.14; and GCV‐CS: 1.485 ± 0.39* with 1.77 fold increase in tissue fluorescence; data are mean±SEM; *n* = 4‐5/group, *p < 0.05, and **p < 0.01 vs their air groups). Removal of senescent cells by GCV administration did now show any reduction in the overall lung tissue fluorescence (Figure 1c). Of note, the tissue luminescence and fluorescence for C57BL/6J mice was used as a background control (Figure S2b) for this experiment.

On an average, the ETS‐exposed mice irrespective of PBS or GCV treatment weighed lesser than the unexposed air controls. However, during the 5‐day duration of GCV administration, we did not observe any drastic decline in the body weight of our animals, thus indicating good tolerance towards the administrated dose in our mice model. (Figure S2a). The serum cotinine (metabolite of nicotine) levels of ETS‐exposed mice groups were shown to be significantly higher than the unexposed air controls (Figure S3b). Furthermore, lung tissue luminescence in the PBS‐treated ETS‐exposed group was significantly higher as compared to the air controls (Figure 1c and the respective relative quantified counts measured using IVIS imaging as PBS‐Air: 846 ± 931; PBS‐ETS: 4133 ± 1994 with 4.89 fold increase in tissue fluorescence; GCV‐Air: 2723 ± 1409; and GCV‐ETS: 4229 ± 2338, data are mean SEM, *p < 0.05 vs their air groups). However, GCV treatment did not change the overall tissue luminescence in the GCV‐treated ETS‐exposed p16‐3MR mice. The luminescence in the C57BL/6J mice was used as background control for this experiment (Figure S2b). Similarly, overall lung tissue fluorescence also did not show any prominent change in our experimental and control groups (Figure S2c,d).

### 3.3 Increased cellular senescence in the lung tissues of CS‐exposed p16‐3MR mice

**FIGURE 2:**
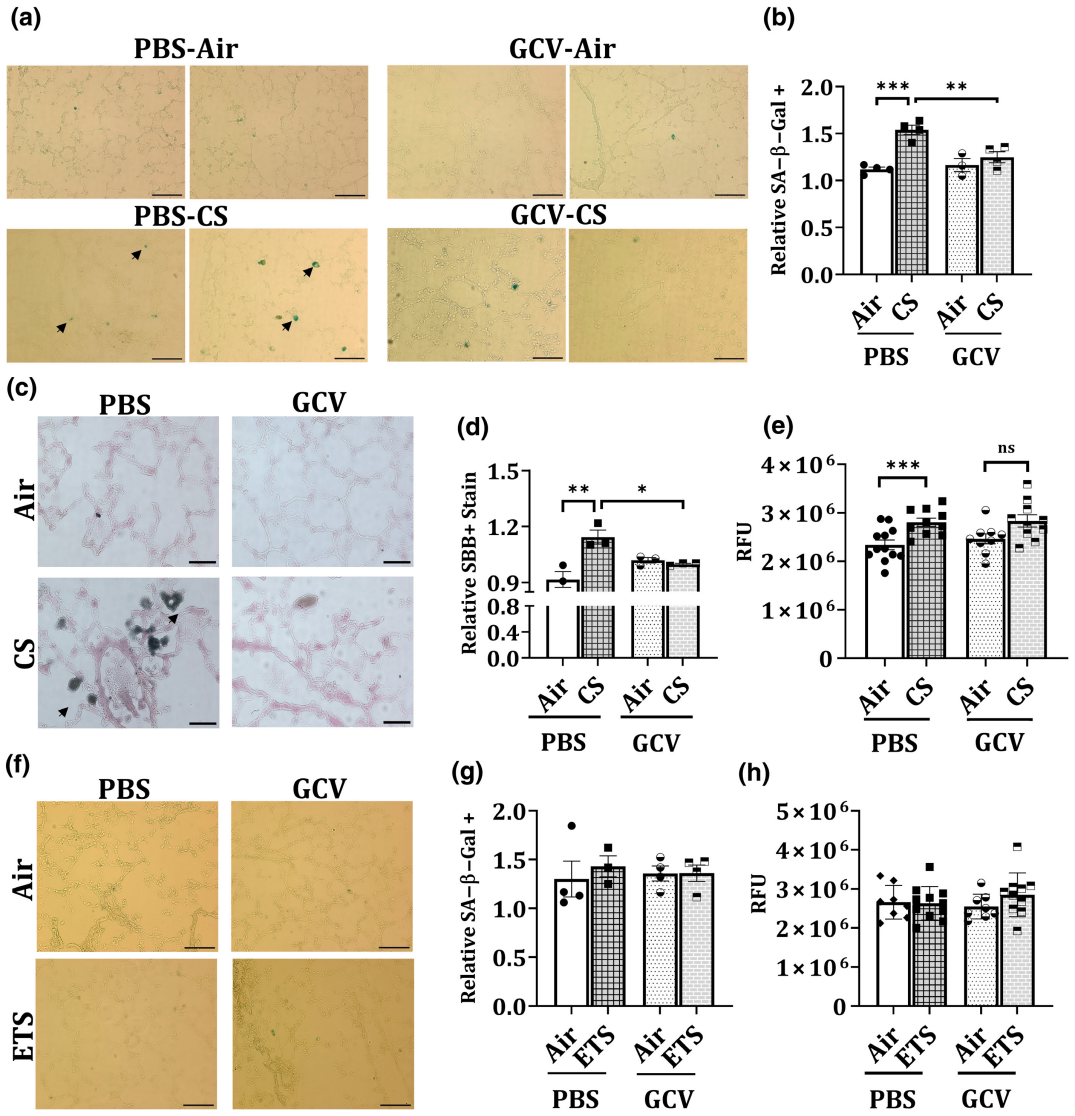
Five‐day GCV treatment significantly reverses CS‐induced cellular senescence in p16‐3MR mice as shown by SA‐β‐Gal and SBB staining. p16‐3MR mice were subjected to chronic (3 months) CS exposure, followed by 5‐day treatment with GCV (25 mg/kg body weight) or PBS (control). The lung tissues from air‐ and CS‐exposed mice were harvested and SA‐β‐Gal staining was performed. Scale bar: 100 μm (20×). Arrows denote senescent cells as stained by SA‐β‐Gal stain. (a) Representative images of *n* = 2–4/group were provided and (b) the quantified counts were plotted as mean ± SEM. SBB staining in OCT‐fixed lung tissue sections was performed. Scale bar: 100 μm (20×). Arrows denote senescent cells as stained by SBB stain. (c) Representative images of *n* = 2–4/group were provided and (d) the quantified counts were plotted as mean ± SEM. SE: *p < 0.05, **p < 0.01 and ***p < 0.001; as per one‐way ANOVA for multiple comparisons. (e) The SA‐β‐Gal activity in the lung tissues from CS‐exposed mice was analyzed using ELISA‐based assay. p16‐3MR mice were subjected to chronic (6 months) ETS exposure, followed by 5‐day treatment with GCV (25 mg/kg body weight) or PBS (control). The lung tissues from air‐ and ETS‐exposed mice were harvested and SA‐β‐Gal staining in OCT‐fixed lung tissue sections was performed. (f) Representative images of *n* = 3–4/group were provided and the (g) quantified counts were plotted as mean ± SEM. Scale bar: 100 μm (20×). (h) The SA‐β‐Gal activity in the ETS‐exposed lung tissues was analyzed using ELISA‐based assay.

Since overall tissue fluorescence alone is not a good measure to determine the overall cellular senescence in the lung tissues, we stained the lung tissues from CS‐ and ETS‐exposed mouse lung with SA‐β‐Gal. Senescence associated β‐galactosidase catalyzes the hydrolysis of β‐galactosides into monosaccharides in senescent cells only (Gary & Kindell, 2005); which can be visualized in blue color under a microscope after staining. We observed a significant increase in the SA‐β‐Gal positive cells in PBS‐treated CS‐exposed p16‐3MR mouse lungs as compared to the air controls. Interestingly, GCV treatment caused a decrease in the SA‐β‐Gal positive cells in CS‐exposed lung samples. In fact, we found a significant decrease in the SA‐β‐Gal positive cells in GCV‐treated CS‐exposed p16‐3MR mouse lung tissue sections as compared to PBS‐treated CS‐exposed lungs (Figure 2a,b), thus proving that GCV‐mediated clearance of p16^+^ senescent cells cause a significant decline in the overall CS‐induced lung tissue senescence in p16‐3MR mice. Interestingly, we also observed significant increase in the overall SA‐β‐Gal activity on treatment CS exposure in p16‐3MR mice (Figure 2e).

We validated our findings by performing SBB staining on OCT‐fixed lung tissue sections. SBB is a histochemical stain for lipofuscin which is known to accumulate in senescent cells (Evangelou & Gorgoulis, 2017). Our results were in concordance with the findings from SA‐β‐Gal staining. While we found a significant increase in the SBB stained cells on CS‐exposure in p16‐3MR mouse lungs, GCV treatment resulted in a significant reduction in the positively stained cell (Figure 2c,d).

On analyzing the blue color staining in the ETS‐exposed lung tissue, we did not see much change in the SA‐β‐Gal staining (Figure 2f,g). In addition, SA‐β‐Gal activity in the lung lysates from ETS‐exposed and unexposed air controls showed no observable change (Figure 2h). Taken together, our results indicate that chronic ETS exposure at the low dose of 10 mg/m^3^ does not induce lung senescence in p16‐3MR mice.

### 3.4 GCV treatment lowers the lung neutrophil infiltration in CS‐exposed young p16‐3MR mice

It is well known that CS skews immune responses that results in lung conditions like COPD, hypertension and cancer in humans (Qiu et al., 2017; Strzelak et al., 2018). Thus, we next studied the immune cell population in the lungs of CS‐ and ETS‐exposed mice. Flow cytometric analyses of the immune cell population from BALF in ETS‐exposed mice did not show much change in the total cell counts. Percentages of neutrophils, CD4^+^ or CD8^+^ T‐cells showed insignificant change, though neutrophil counts were higher in the BALF of air‐ and ETS‐exposed mice (Figure S4a).

**FIGURE 3:**
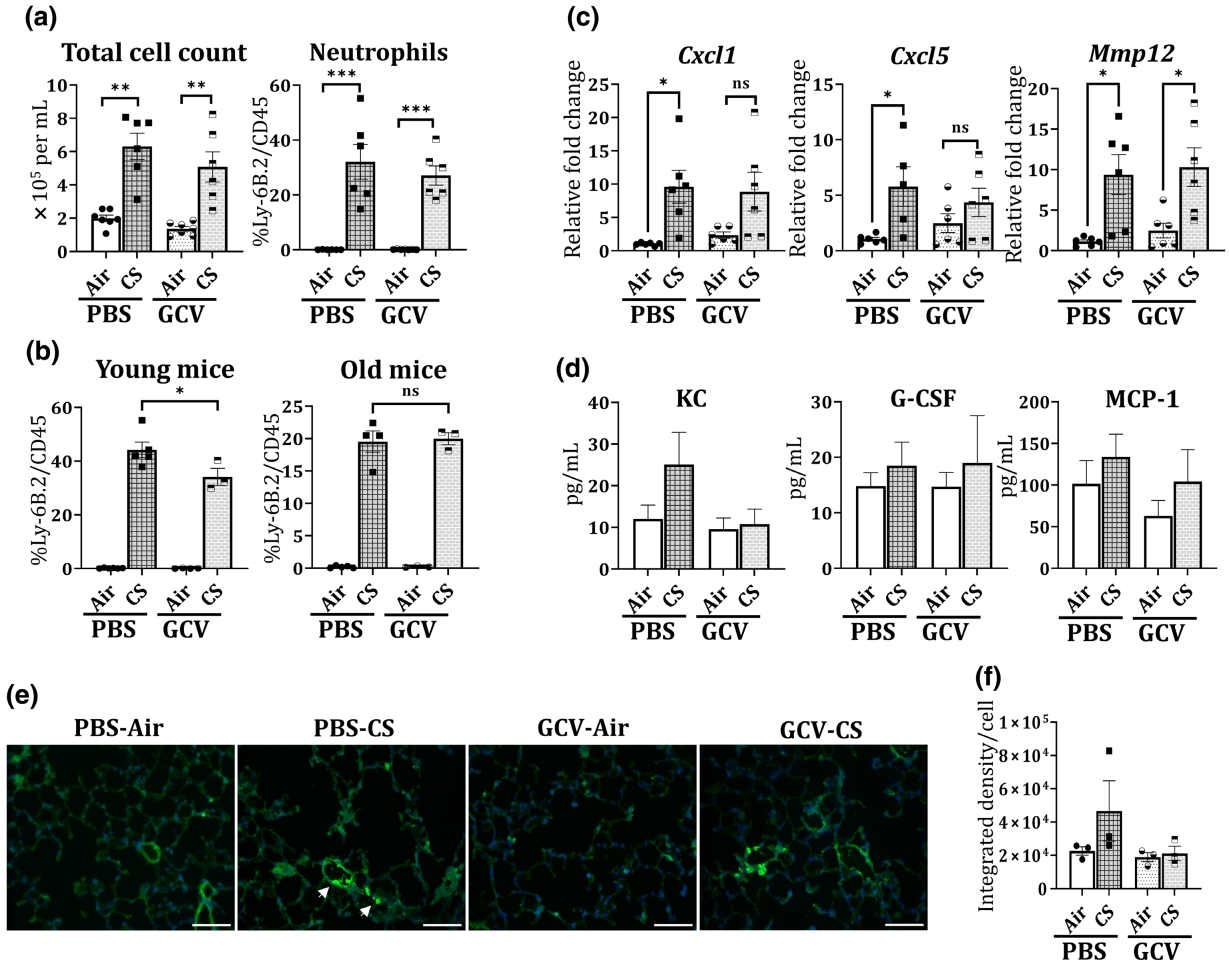
GCV treatment reverts the CS‐induced neutrophilic increase in the BALF of young p16‐3MR mice. BALF from the lungs of PBS/GCV‐treated and air/CS‐exposed (3 months) p16‐3MR mice was obtained. Total cell count was determined using AO/PI staining. Flow cytometry was used to determine the immune cell population (neutrophils, CD4^+^ and CD8^+^ T‐cells) in BALF from control and treated mice. (a) The changes in the total and neutrophil population was plotted as bar graph. Data are shown as mean ± SEM (*n* = 6–7/group); **p < 0.01 and ***p < 0.001 between groups, per one‐way ANOVA for multiple comparisons. (b) The effect of CS‐exposure on neutrophil number was re‐plotted for young and old mice. Data are shown as mean ± SEM (*n* = 3–5/group); *p < 0.05 versus PBS‐CS, per one‐way ANOVA for multiple comparisons. ns: not significant. The total RNA from the lung tissues of PBS/GCV‐treated and air/CS‐exposed (3 months) p16‐3MR mice was obtained. (c) Alterations in the mRNA expression of *Cxcl1*, *Cxcl5* and *Mmp12* gene expression was determined using qPCR. 2^−∆∆Ct^ method was used to determine the changes in the gene expression and fold changes relative to PBS‐Air were plotted. Data are shown as mean ± SEM (*n* = 5–6/group). SE: *p < 0.05 versus PBS‐Air; as per one‐way ANOVA for multiple comparisons. Here; ns: not significant. (d) The levels of inflammatory cytokine/chemokine were determined using Luminex and levels of KC, G‐CSF and MCP‐1 plotted in pg/mL. Data are shown as mean ± SEM (*n* = 7–11/group). Lung tissue slices were stained with Ly6 B.2 and DAPI nuclear stain. Increased infiltration of neutrophils into the CS‐exposed mouse lungs was studied under fluorescence microscope. (e) Representative images of *n* = 3/group were provided and the (f) integrated fluorescence density per cell was plotted as mean ± SEM. Scale bar: 100 μm (20×). Arrows indicate Ly6 B2 stained cells (neutrophils) on CS exposure.

Contrary to the ETS findings, a marked increase in the total cell count, as denoted by AO/PI staining, was observed in the CS‐exposed PBS/GCV treated mouse lungs thus showing increased immune cell influx into the lungs on CS exposure. Flow cytometric analyses showed a significant increase in the neutrophil population in BALF from CS‐exposed mouse lungs. When pooled together (young and old), GCV treatment did not affect the neutrophil counts in the BALF from CS‐exposed mice (Figure 3a). However, senescence is a complex mechanism affected by age and gender which need to be taken in account. Thus, we analyzed our data based on the ages of the mice where 4‐ to 6‐month‐old mice were considered as ‘young’ while 7‐ to 12‐month‐old mice were tagged as ‘old’. We found that while the GCV treatment did not affect the CS‐induced increase in neutrophil population in old p16‐3MR mice, it significantly lowered the neutrophil influx into the mouse lungs on CS‐exposure in the younger mice (Figure 3b). This is an interesting observation and requires further work to deduce its true implications.

While we found an increase in the T‐cell population (CD4^+^ and CD8^+^) on CS‐exposure, the observed changes were not significant. We also did not observe any change in the T‐cell population on GCV treatment in our control and CS‐exposed BALF samples (Figure S4c), thus indicating that CS exposure does not affect senescence in T‐cell population.

To validate our findings through flow cytometry, we performed gene expression analyses on RNA obtained from lung tissues of air‐ and CS‐exposed PBS/GCV‐treated mice. We observed a significant increase in the transcript levels of *Cxcl1* and *Cxcl5* genes in PBS‐treated CS‐exposed p16‐3MR mice as compared to control. We observed approximately 8% and 25% decline in the gene expressions of *Cxcl1* and *Cxcl5* respectively on GCV treatment. Similarly, the gene expression of *Mmp12* was increased significantly on CS‐exposure in p16‐3MR mice. However, no change in *Mmp12* expression was observed on GCV‐treatment in our mouse model (Figure 3c). Assessment of cytokine/chemokine production further validated the mRNA results by showing a two‐fold increase in the KC levels in the BALF of CS‐exposed mice as compared to unexposed air controls. Importantly, removal of p16^+^ senescent cells using GCV resulted in approximately 57% reduction in the levels of KC as shown in Figure 3d. Luminex data also revealed 59%, 8% and 23% decline in the levels of eotaxin, MIP‐1α and IL‐12p40 in GCV‐treated CS‐exposed p16‐3MR mice as compared to PBS‐treated CS‐exposed group (Table 1). This shows that removal of p16^+^ senescent cells does result in alleviation of CS‐induced inflammation.

**TABLE 1:** Chemokine/cytokine levels in BALF as observed by Luminex system.

Cytokine/chemokine	PBS‐air (mean ± SD)	PBS‐CS (mean ± SD)	GCV‐air (mean ± SD)	GCV‐CS (mean ± SD)
IL‐3	1.18 ± 0.47	0.99 ± 0.19	0.92 ± 0.37	0.88 ± 0.48
IL‐1β	0.94 ± 0.21	0.92 ± 0.16	0.88 ± 0.16	0.89 ± 0.13
TNF‐α	8.74 ± 2.71	8.37 ± 1.14	7.42 ± 2.08	7.66 ± 2.46
IFN‐γ	2.33 ± 1.23	1.71 ± 0.51	2.01 ± 1.23	1.61 ± 0.95
IL2	2.99 ± 1.09	2.85 ± 0.84	2.74 ± 0.85	2.52 ± 1.28
IL‐13	17.11 ± 13.79	11.9 ± 11.38	17.47 ± 11.89	15.59 ± 11.45
IL‐5	1.82 ± 0.82	1.84 ± 0.38	1.56 ± 0.88	1.52 ± 0.85
IL‐1α	3.33 ± 1.39	3.40 ± 1.10	2.77 ± 1.24	2.86 ± 1.72
RANTES	7.99 ± 2.20	7.98 ± 2.10	7.74 ± 3.02	6.65 ± 2.79
IL‐10	1.67 ± 1.77	1.71 ± 1.56	1.88 ± 1.90	1.79 ± 1.83
IL‐17	1.14 ± 0.37	1.24 ± 0.16	0.98 ± 0.28	1.20 ± 0.28
Eotaxin	8.03 ± 4.08	16.45 ± 17.53	12.92 ± 12.25	6.662 ± 4.06
MIP‐1α	12.16 ± 14.46	14.25 ± 10.68	8.09 ± 9.10	13.11 ± 12.45
IL‐12p40	114.83 ± 98.59	122.32 ± 68.24	100.92 ± 88.73	94.41 ± 87.74
IL‐12p70	3.75 ± 3.37	2.50 ± 2.27	2.23 ± 3.63	2.16 ± 1.46

We further used immunofluorescence to study the infiltration of neutrophils into the lungs of CS exposure. Immunofluorescence results showed two‐fold increase in the Ly6 B2 fluorescence in PBS‐CS group as compared to the unexposed air controls. This induction was reduced by 54% on removal of p16^+^ senescent cells on GCV administration (Figure 3e,f).

### 3.5 Clearance of senescent cells using GCV restores mitochondrial function in CS‐exposed p16‐3MR mice

**FIGURE 4:**
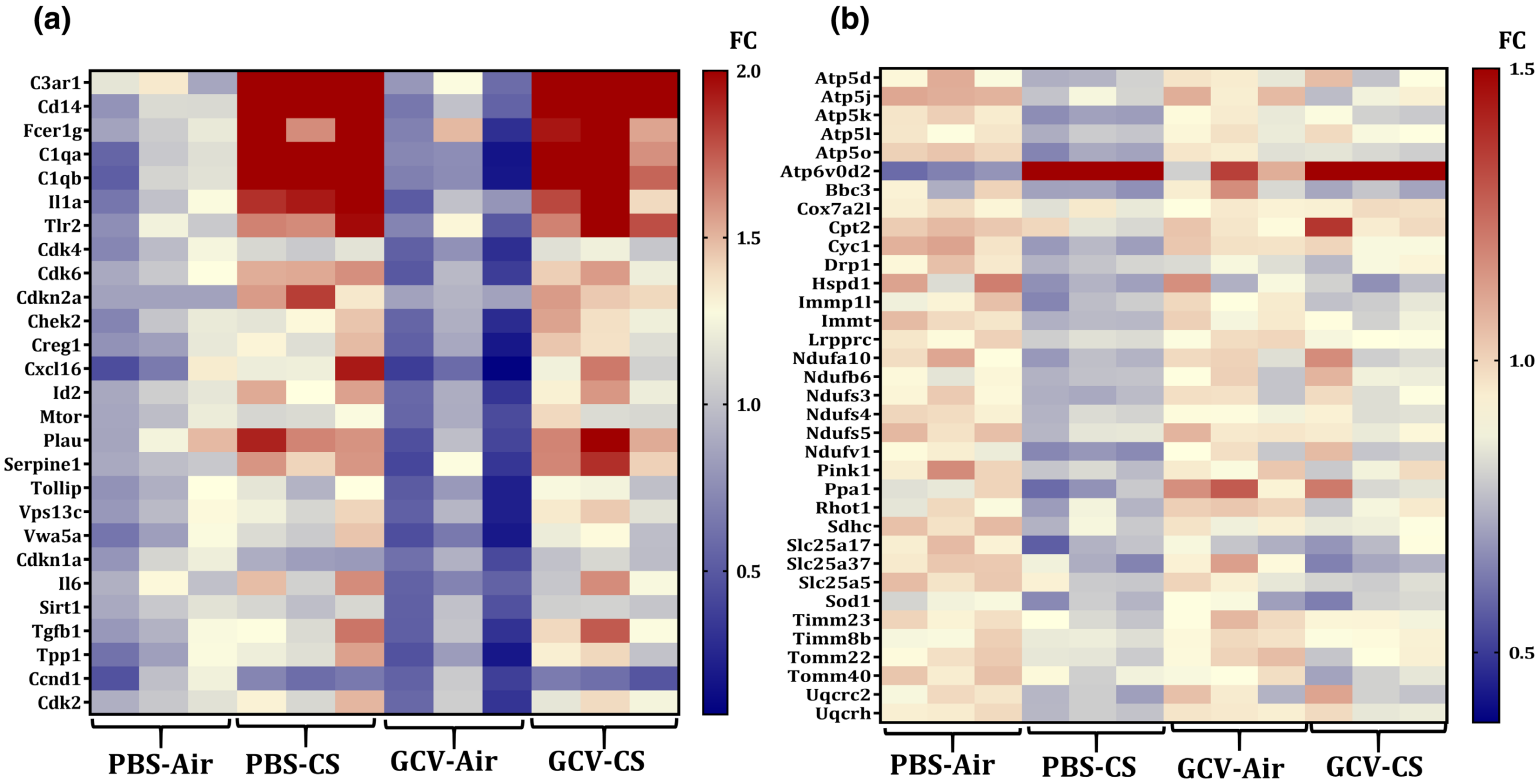
Transcript levels of senescence and mitochondrial function‐associated genes are altered in PBS/GCV treated CS‐exposed p16‐3MR mice. Total RNA was isolated from the lungs of PBS/GCV treated and air/CS‐exposed (3 months) p16‐3MR mice. Our customized NanoString panel (senescence) was used to screen the potential targets via nCounter SPRINT Profiler. Changes in the normalized counts of respective genes were obtained relative to PBS‐Air group and plotted as heat map. Heat map showing fold changes (FC) in the expression of (a) senescence and (b) mitochondrial function associated genes is graphed relative to PBS‐Air group.

We next looked into the molecular mechanism that might be affected by GCV‐mediated clearance of p16^+^ senescent cells in CS‐exposed p16‐3MR mice. For this the mRNA transcript levels of senescence and mitochondrial function‐associated genes using custom‐designed Nanostring panels were assessed. When comparing the gene expressions of various genes in the senescence panel, we found significant upregulation of the complement system genes (*C3ar1*, *CD14*, *Fcerg1*, *C1qa* and *C1qb*) in CS‐exposed p16‐3MR mice as compared to the air controls. We also found upregulation of senescence associated genes (*p16* and *SERPINE1*) and SASP factor (like, *IL‐1α*) in CS‐exposed p16‐3MR mice (Figure 4a; Figure S5).

Since mitochondrial dysfunction is a major driver of cellular senescence (Chapman et al., 2019; Miwa et al., 2022), we next tested the expression of mitochondrial function related genes in our experiment. Our analyses revealed a very interesting trend as demonstrated through the heat map in Figure 4b. We found decline in the transcripts levels of genes associated with mitochondrial complex (*Ndufs4*, *Ndufs5*), Ca^2+^ transport (*Cpt2*) and ATP synthesis (*Atp5d*, *Atp5l*) in CS‐exposed p16‐3MR mice. Importantly, GCV treatment reversed the CS‐induced changes in the transcript levels of these genes (Figure S6). This provides evidence for restoration of CS‐induced mitochondrial dysfunction on removal of p16^+^ senescent cell. However, further work is required to support this finding.

### 3.6 Clearance of senescent cells restores the CS‐induced airspace enlargement in young p16‐3MR mice

**FIGURE 5:**
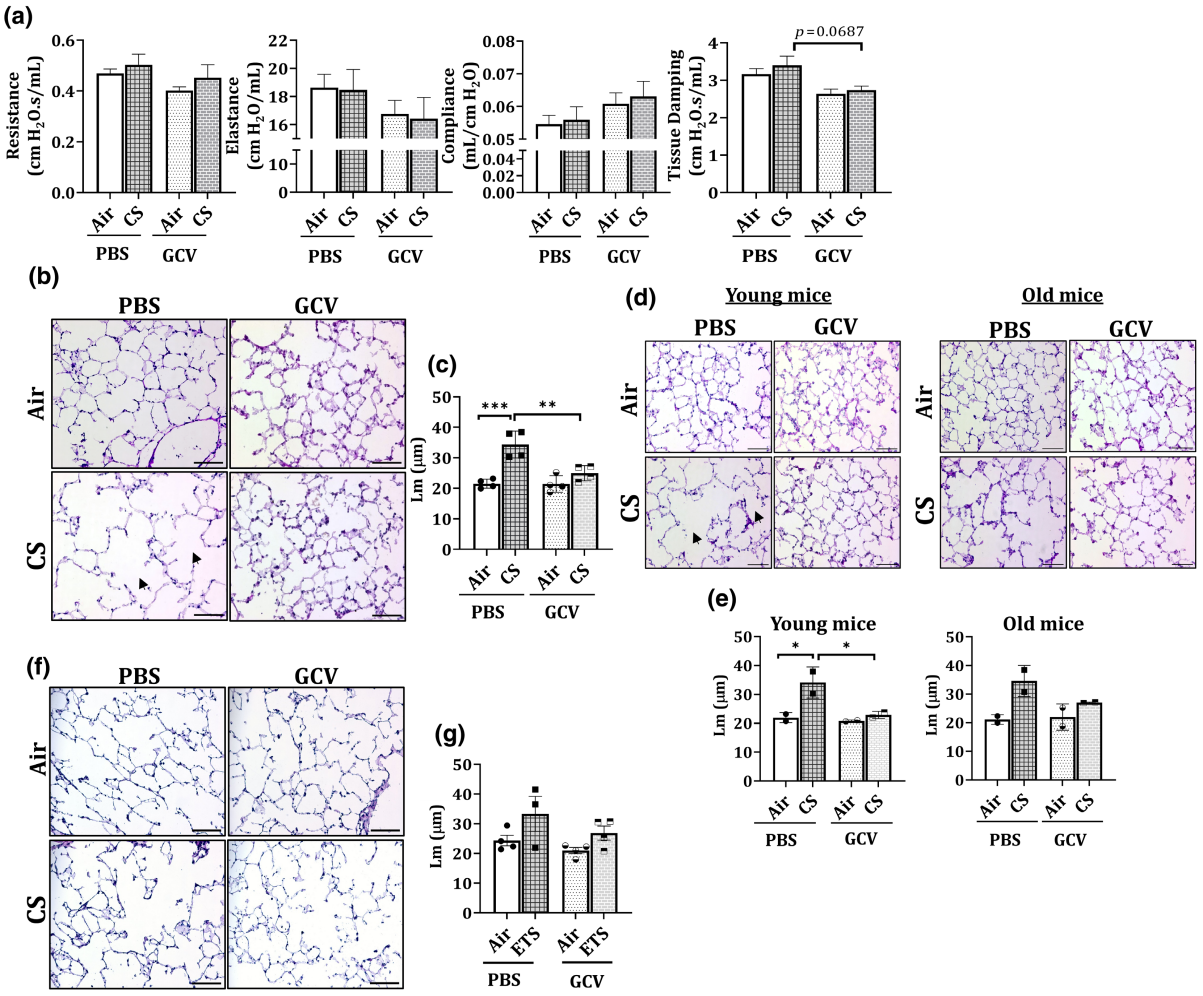
GCV treatment alters tissue damping and reverses CS‐induced airspace enlargement in the lungs of young p16‐3MR mice. p16‐3MR mice were subjected to chronic (3 months) CS exposure, followed by 5‐day treatment with GCV (25 mg/kg body weight) or PBS (control). (a) Lung function parameters of PBS/GCV treated air/CS‐exposed p16‐3MR mice were determined using SCIREQ's flexiVent system. Data are shown as mean ± SEM (*n* = 6–7/groups). *p*‐value calculated as per one‐way ANOVA for multiple comparisons. The lung tissues from air‐ and CS‐exposed mice were harvested and H&E stained. (b) Representative images of *n* = 4/group were provided and the (c) quantified counts were plotted as mean ± SEM. Scale bar: 100 μm (20×). Changes in the observed Lm values for young and old mice was analyzed and plotted. Scale bar: 100 μm (20×). (d) Representative images of *n* = 2/group were provided and the (e) quantified counts were plotted as mean ± SEM. Arrows denote airspace enlargement in the lung on CS‐treatment. SE: *p < 0.05, **p < 0.01, ***p < 0.001; as per one‐way ANOVA for multiple comparisons. p16‐3MR mice were subjected to chronic (6 months) ETS exposure, followed by 5‐day treatment with GCV (25 mg/kg body weight) or PBS (control). The OCT sections from air‐ and ETS‐exposed mouse lungs were H&E stained. (f) Representative images of *n* = 3–4/group were provided and the (g) quantified counts (bottom) were plotted as mean ± SEM. Scale bar: 100 μm (20×).

Next, we tested the effect of CS vand ETS exposures on lung function (mechanical properties) parameters and lung histology in our mouse models. On studying the lung function changes in CS‐exposed groups, we did not observe any noticeable change in the tissue resistance, elastance or compliance in CS‐exposed p16‐3MR mice as compared to air controls, which is not surprising due to shorter duration of CS‐exposure. Though not significant, the values of elastance and resistance showed decreasing trends, whereas compliance showed increasing trend in air and CS‐exposed mice. Importantly, we found a near significant (*p* = 0.0687) decrease in the tissue damping observed in the lung tissues of GCV‐treated CS‐exposed mice as compared to PBS‐treated controls (Figure 5a). This points towards some phenotypic changes in our mouse model on clearance of senescent cells.

To determine further, we studied the lung morphology using H&E stained tissue sections. We found a significant increase in the mean linear intercept (Lm) values for CS‐exposed mouse lungs as compared to air controls. GCV treatment showed a significant decrease in the CS‐induced airspace enlargement (Figure 5b,c; full scan in Figure S7). Again, when young (4–6 months) and old (7–12 months) mice data were analyzed individually, we found a significant decline in the Lm values for GCV‐treated CS‐exposed young p16‐3MR mice (Figure 5d,e), thus proving that clearance of senescent cells could improve the CS‐related disease phenotype in younger age‐groups. Based on this finding, future work must include different age groups (preferably younger) of mice to study the efficacy of using senolytics/senomorphics in smoke‐induced lung pathologies.

Supporting our previous data from immune cell composition in BALF and lung cellular senescence, we did not see any pronounced change in the mean linear intercept (Lm) of the airspace in the ETS‐exposed p16‐3MR mice as compared to unexposed air controls (Figure 5f,g). Interestingly, we observed a significant increase in the lung tissue elastance and a decline in the lung compliance in PBS‐treated ETS‐exposed p16‐3MR mice. Furthermore, ETS‐induced changes in lung elastance (*p* = 0.058) and compliance (*p* = 0.071) were further reversed on GCV‐treatment as shown in Figure S4b.

### 3.7 GCV treatment results in reversal of EMT‐related genes in CS‐challenged p16‐3MR mice

**FIGURE 6:**
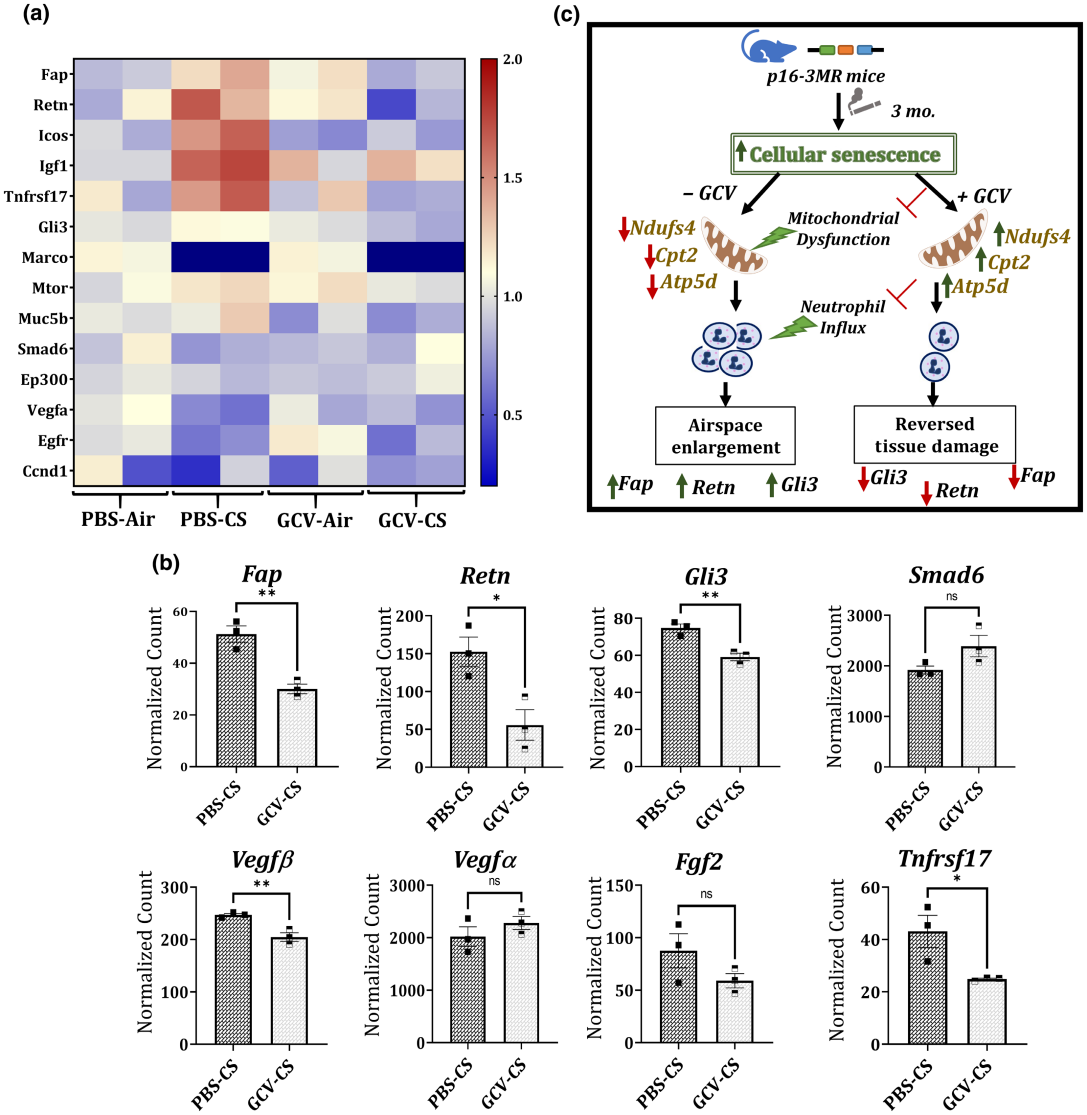
GCV treatment reverses the genes associated with EMT in CS‐exposed p16‐3MR mice. Total RNA was isolated from the lungs of PBS/GCV treated and air/CS‐exposed (3 months) p16‐3MR mice. Our customized NanoString panel to study lung regeneration was used to screen the potential targets via nCounter SPRINT Profiler. Normalization of absolute RNA count and data analysis were done by nSolver software. The overview of all the dysregulated targets is shown as a (a) heatmap and (b) selected gene transcription changes between PBS‐CS versus GCV‐CS are shown separately. Data are shown as mean ± SEM (*n* = 3/group); *p < 0.05, **p < 0.01 versus PBS‐CS, per unpaired *t* test for pairwise comparison. Here; ns: not significant. (c) Schematics demonstrating how removal of p16^+^ senescent cells in CS (3 months)‐exposed p16‐3MR mice causes reduction in neutrophilic inflammation, CS‐induced mitochondrial dysfunction and tissue damage.

In order to study how the GCV treatment improves airspace enlargement in young p16‐3MR mice, we tested the RNA expression of various growth factors and EMT related genes in our control and treatment groups. Interestingly, we found marked changes in genes involved in various cellular processes like cell differentiation and EMT as shown in Figure 6a. Importantly, we found significant reduction in genes involved in EMT (*Gli3*, *Fap*, and *Retn*) and inflammation (*TNFrsf17*) in GCV‐treated CS‐exposed mice as compared to the PBS‐treated CS‐exposed p16‐3MR mice (Figure 6b).

## 4 DISCUSSION

Chronic obstructive pulmonary disease (COPD) is characterized by emphysema and chronic bronchitis with poorly reversible airway obstruction that causes difficulty breathing amongst the patients. Cigarette/tobacco smoking is the main etiological factor for the disease and the known treatment for COPD is palliative. The disease is associated with chronic inflammation, accelerated lung aging and abnormal tissue repair on oxidative damage. However, the actual mechanism underlying the occurrence of the disease remains unknown (Barnes et al., 2015). In this respect, cellular senescence is considered to be crucial, as accumulation of senescent cells has been associated with various age‐related pathologies including those of the lung (He & Sharpless, 2017; Meiners et al., 2015). Cellular senescence is defined as a state of growth arrest in cells which can be triggered by either intrinsic factors (like, telomere shortening) or extrinsic factors (like, oxidative stress due to exposure). Key feature of cellular senescence includes activation of cell cycle pathways regulated by tumor suppressors including p16, p21 and p53 (Antony & Thannickal, 2018).

Considering this, it is obvious to consider that cellular senescence has a major role to play in the pathophysiology of COPD. In fact, various groups have shown increased expression of p16 and p21 along with heightened senescence in lung tissues on CS exposure in mouse models of COPD (Cottage et al., 2019; Sundar et al., 2018). However, despite these advances it is hard to study lung cellular senescence in response to CS exposure, due to the lack of a good mouse model. For instance, previous work from our group showed that complete gene knockout of p16^INK4A^(p16^−/−^) results in tumor generation, while the knockdown of p16 expression does not show any protection against cellular senescence in mouse models of COPD (Sundar et al., 2018). To overcome these shortcomings, Demaria et al. (2014) developed a new mouse model with a trimodality reporter gene with a p16^INK4a^ gene promoter. This model allows the identification, isolation and selective killing of p16^+^ senescent cells on GCV treatment, thereby making it a powerful model to study senescence without genetically modifying the gene expression. We previously tested the suitability of this model to study CS‐related lung pathologies. We found increased tissue luminescence and fluorescence in the mouse lung tissues exposed to CS for 30‐day duration. Gene and protein expression data also supported the successful induction of p16 pathway and SASP genes on CS exposure, thereby confirming that p16‐3MR is suited to study CS‐related pathologies (Kaur et al., 2021). In the current study, we were interested in determining if elimination of ETS or CS‐induced senescent cells could result in any reversal of the ETS or CS‐mediated inflammation and lung damage.

We found insignificant changes in the lung cellular senescence or lung inflammation (though lung neutrophil influx was prominent) on ETS exposure. One of the causes of this, could be that we used very low (10 mg/m^3^) volume of TPM for this exposure. Also, owing to the occurrence of the global pandemic of COVID‐19 while conducting these mouse exposures, the exposures had to be halted and/or intermittently exposed which might have affected the end points. However, evidence of slight airspace enlargement and decreased lung compliance in the ETS‐exposed p16‐3MR mice point towards acute lung injurious responses in these mice.

We observed more conclusive and pronounced effects in our model of CS exposure. Initial studies using IVIS multispectral imaging revealed increase in the overall tissue fluorescence of CS‐exposed mouse lung. It is pertinent to mention here that IVIS imaging captures the fluorescence in whole tissue and would not indicate the changes at cellular level. Hence, we furthered our study by taking a closer look at the senescence mechanism in our mouse lung samples.

We employed two independent techniques of SA‐β‐Gal staining and SBB staining to prove increased number of senescent cells in the lung tissues of CS‐exposed p16‐3MR mice. We further showed removal of these senescent cells on 5‐day treatment with GCV thus providing proof of concept of elimination of senescence on GCV treatment in p16‐3MR mouse model. It is pertinent to mention here that each time we use GCV‐mediated clearance of senescent cells in this study, we are referring to p16^+^ cells which will be eliminated in this mouse model due to the virtue of the presence of HSV‐TK reporter. Implications of our results are thus to be understood only in light of p16‐mediated regulation of cellular senescence.

To understand the mechanistic implications of this finding, we also studied the immune cell infiltration in the BALF from lungs of control and treated mice. Airway neutrophilia is one of the key inflammatory responses due to CS in COPD. Elevated neutrophil counts correlate to decline in forced expiratory volume in 1 s (FEV_1_), airway obstruction, and development of emphysema in COPD (Hoenderdos & Condliffe, 2013; Jasper et al., 2019). Considering this, we studied the effect of CS exposure on the neutrophil count in the BALF from CS‐exposed mice. As expected, we found a significant increase in the neutrophil count in BALF from PBS‐treated CS‐exposed p16‐3MR mouse lung as compared to air control. Interestingly, this CS‐induced influx of neutrophils was ameliorated on clearance of senescent cells using GCV in young p16‐3MR mice. In fact, the inflammatory response on CS exposure in younger mice was also more pronounced in younger mice as compared to their older counterparts. Gene expression and cytokine production data confirmed that clearance of p16^+^ senescent cells following GCV treatment reverts the CS‐induced expression of *CXCL1* in the lungs and levels of KC in the BALF of p16‐3MR mice. This is an important observation as it points towards increased neutrophilic senescence on CS exposure in p16‐3MR mice. While CS is known to induce neutrophilic influx into the lung tissues, one of the reasons for its inability to provide innate immunity is reduced migratory accuracy of these neutrophils (Deng et al., 2022; Jasper et al., 2019). Old age has been known to cause similar effects on circulating neutrophil population thus hampering the innate immune response at an old age (Barkaway et al., 2021). Though there have been numerous contentions to describe the mechanisms underpinning such defects in the neutrophil function in COPD, the exact role of immunosenescence on CS exposure has not been known. To our knowledge, we for the first time provide proof of a potential increase in senescent neutrophil population on CS‐exposure which was cleared on treatment with GCV and could be responsible for their loss of migratory ability. Future work in this direction is required to understand how cellular senescence affects the functional abilities of neutrophils (immunosenescence) on smoke exposure.

Gene expression analyses showed significant upregulation in the expression of complement system genes (*C3ar1*, *CD14*, *Fcerg1*, *C1qa* and *C1qb*) in CS‐exposed p16‐3MR mice as compared to controls. Of note, complement system is known to be activated by NETosis (neutrophils forming neutrophil extracellular traps [NET]) (Yuen et al., 2016). In fact, enhanced NET formation in the sputum of COPD patients has been reported earlier (Grabcanovic‐Musija et al., 2015; Pedersen et al., 2015). Taken together, it can be speculated that there exists a link between the increased milieu of senescent neutrophils and upregulation of complement system in the lungs on CS exposure which eventually leads to enhanced NETosis in COPD.

Furthermore, we found evidence of reversal in CS induced alterations in mitochondrial function related genes on GCV treatment thus proving that the two phenomena are inter‐related. Mitochondrial functional defects have been known to induce senescent phenotype termed as mitochondrial dysfunction‐associated senescence (MiDAS) (Gallage & Gil, 2016; Miwa et al., 2022). In fact, growing body of evidence shows that there exists a bidirectional role of mitochondrial dysfunction in regulation of cellular senescence. Mitochondrial imbalance is considered to be an epiphenomenon of cellular senescence (Vasileiou et al., 2019; Yoon et al., 2003, 2006). In this regard, CS is known to cause increased production of reactive oxygen species (ROS) within the cells; which is mainly attributed to dysregulated mitochondria. Increased mitochondrial ROS can in turn cause DNA damage thereby, leading to increased cellular senescence (Hoffmann et al., 2013; Wang et al., 2022). In this regard, our results show changes in expression of genes encoding for mitochondrial complex (*Ndufs4*, *Ndufs5*), Ca^2+^ transport (*Cpt2*) and ATP synthesis (*Atp5d*, *Atp5l*) in CS‐exposed PBS‐treated p16‐3MR mouse lungs. More importantly, GCV treatment was shown to significantly reverse the CS‐induced alteration in the expression of these respective genes, thus proving a role of MiDAS in CS‐associated senescence in vivo. Also, it is important to note that there were a few targets which were affected on treatment with GCV alone and may not have any association to cellular senescence. However, since GCV is employed in this study due to inclusion of p16‐3MR as a mouse model, these changes might not be of relevance for studying the effect of senolytics/senomophics as a therapy for CS‐induced pathologies. Taken together, our results show that removal of senescent cells using GCV might affect important metabolic processes like complement system and mitochondrial function. Further work is required in this area to identify the key players regulating this phenomenon and serving as therapeutic target in conditions like COPD.

Finally, we studied the phenotypic effects of the clearance of senescent cells in CS‐exposed p16‐3MR mice. Lung function analyses revealed a substantial reduction in the tissue damping in the lungs of CS‐exposed GCV‐treated mice as compared to PBS controls. In lungs, tissue damping is closely associated with tissue resistance and is characterized by viscoelasticity. In COPD, an increase in lung elastance might result in higher values of tissue damping than healthy controls. In a prior study conducted by Copot and colleagues, it was shown that considering the heterogeneous nature of structural changes during COPD, changes in tissue mechanics on occurrence of COPD could be related to viscoelastic properties (tissue damping) (Copot et al., 2017). In light of this, our results provide early evidence of changes in tissue mechanics on clearance of senescent cells in CS‐exposed mice. Furthermore, we also illustrate GCV‐mediated reversal of CS‐induced airspace enlargement in young p16‐3MR mice. This is an encouraging find as it indicates that if detected early, there is a possibility of reversing the tissue damage caused by CS‐exposure at a young age. We validated our histological results by performing gene expression analyses for *MMP12* in the lung tissues from air and CS‐exposed mouse lungs. MMP12 is a matrix metalloproteinase that has been shown to be upregulated on smoke exposure. Excess MMP12 has been associated with lung injury and emphysema in mice (Churg et al., 2012; Spix et al., 2022). Our results indicated a significant increase in the gene expression of *MMP12* on CS exposure in p16‐3MR mice. However, we did not observe any change in the *MMP12* mRNA expression on removal of senescent cells using GCV. This proves that CS‐mediated upregulation of *MMP12* is independent of increased cellular senescence.

To explain the observed tissue repair in young GCV‐treated CS‐exposed p16‐3MR mice, we studied the expression of genes involved in lung regeneration and repair. We found significant downregulation of EMT‐related genes like *Retn*, *FAP* and *Gli3* on GCV treatment in CS‐exposed mice as compared to the PBS control. Resistin (Retn) belongs to the RELM family of proteins that is known to be responsible for the Th2‐activated macrophage inflammation in response to tissue injury (Acquarone et al., 2019; Lin & Johns, 2020). It is known to be upregulated in allergen‐induced pulmonary vascular remodeling by induction of EMT (Fan et al., 2015). Likewise, fibroblast activation protein is associated with worse clinical outcomes in fibrosis, arthritis and cancer due to its effects on extracellular remodeling, EMT and intracellular signaling (Fitzgerald & Weiner, 2020). Gli3 has also been identified as a key regulator of EMT in various cancers (Matissek & Elsawa, 2020; Shen et al., 2021). Our results show significant decrease in the expression of all of these proteins thus proving that GCV‐mediated removal of p16^+^ senescent cells result in reversal of CS‐induced epithelial‐to‐mesenchymal transition in p16‐3MR mice. Interestingly, all these genes have been shown to modulate PI3/Akt signaling downstream (Acquarone et al., 2019; Fitzgerald & Weiner, 2020; Lo Ré et al., 2012). Previous reports have linked the PI3/Akt pathway in reverting the CS‐induced emphysema (Cottage et al., 2019), thus supporting our findings.

We also find changes in the expression of vascular endothelial growth factor (VEGFA and VEGFB) on GCV treatment. VEGF is not only important for angiogenesis, but also lung development (Li, 2010; Medford & Millar, 2006). Thus, we provide proof for involvement of EMT‐related proteins and VEGF‐mediated angiogenesis in regulating the GCV‐mediated tissue repair in our model. Further work is needed to shed light on the mechanism of lung tissue repair on removal of senescent cells in lung tissues damaged by CS exposure in vivo. Nevertheless, our results provide evidence for the first time that removal of senescent cells could have therapeutic implications in CS‐induced lung pathologies and have the potential of reverting the tissue damage caused due to environmental stressors.

While we provide proof‐of‐concept showing (a) successful removal of senescent cells using GCV, and (b) reversal of CS‐induced mitochondrial damage on removal of senescence thereby leading to lowering of CS‐induced airspace enlargement; our study had a few shortcomings. Our results are a testament to the fact that age is crucial when studying lung cellular senescence during long‐term environmental exposures (in this case CS exposure) in vivo. In light of this, inclusion of much younger (2–4 months) mice would have shed more insights onto the mechanism of acquired and innate cellular senescence in mouse lungs. While we observe few phenotypic changes (e.g., airspace enlargement) and immune responses (e.g., neutrophilic infiltration) in this study, some of the classic changes observed during COPD/emphysema (e.g., increased resistance and decreased elastance) were not observed in this work, due to shorter span of exposure to CS. One of the reasons for the shorter duration of exposure was to limit the effects of innate cellular senescence in our exposed mice lungs. However, based on the findings from this work, future studies could target prolonged exposure to understand the correlation between MiDAS and immunosenescence on CS‐exposure. Also, in this study we entirely focused on p16‐mediated senescence response due to the nature of the mouse model being studied. However, there could be other players (p21 or p53) that could have an important role in modulating CS‐mediated senescence. Further work to deduce the role of other senescent markers in COPD is thus warranted. It is also important to note that due to the lack of valid markers to indicate induction of cellular senescence we had to base our test results on the findings from SA‐β‐Gal and SBB staining. While these are well‐known markers for senescence, they are known to have various shortcoming as well, which must be considered while interpreting our study outcome (González‐Gualda et al., 2021).

In conclusion, we for the first time, shows induction of lung cellular senescence on CS exposure in p16‐3MR mice. We showed that clearance of CS‐induced senescent cells could revert the neutrophilic influx in younger p16‐3MR mice. Furthermore, this clearance results in restoring the cellular homeostasis by upregulating genes relating to mitochondrial function in CS‐exposed p16‐3MR mice. Interestingly, it could also repair the CS‐induced airspace enlargement in CS‐exposed young p16‐3MR mice (Figure 6c). Overall, this work provides proof that use of senolytics/senomorphics might be beneficial in treating CS‐induced pathologies, such as COPD/emphysema.

## AUTHOR CONTRIBUTIONS

Gagandeep Kaur designed and conducted the experiments, wrote and revised the manuscript. Thivanka Muthumalage planned and performed environmental tobacco exposure, assisted with lung function and flow cytometry, and edited the manuscript. Irfan Rahman conceptualized the research, obtained research funding, and edited the manuscript.

## FUNDING INFORMATION

This study was supported by the National Institutes of Health (NIH) 1R01HL135613, R01HL133404, and R01ES029177, and TriState SenNet U54 AG075931. The funding body has no role in design of the study, data collection, analysis, and interpretation of data and in writing the manuscript.

## CONFLICT OF INTEREST STATEMENT

The authors have declared that no competing interest.

## Supporting information


**Data S1:** Supporting Information

## Data Availability

All data and materials are described in the manuscript.
