# Supplementary material for: Clearance of senescent cells reverts the cigarette smoke‐induced lung senescence and airspace enlargement in p16‐3MR mice
Source: Aging Cell. 2023 Apr 20;22(7):e13850. doi: 10.1111/acel.13850 (PMC10352560; doi:10.1111/acel.13850)
Supplement: Supplementary file 1 — Data S1: Supporting Information [file ACEL-22-e13850-s001.docx]

**Supplementary Information File**

**Clearance of senescent cells reverts the cigarette smoke-induced lung senescence and airspace enlargement in p16-3MR mice**

Gagandeep Kaur^1,^ Thivanka Muthumalage^1^ and Irfan Rahman^1^

^1^Department of Environmental Medicine, University of Rochester Medical Center, Rochester, NY

^*^Correspondence should be addressed to:

Irfan Rahman, Ph.D.

Department of Environmental Medicine

University of Rochester Medical Center

Box 850, 601 Elmwood Avenue

Rochester 14642, NY, USA

Tel: 1 585 275 6911

E-mail: [irfan_rahman@urmc.rochester.edu](mailto:irfan_rahman@urmc.rochester.edu)

**Supplementary Figures**

**
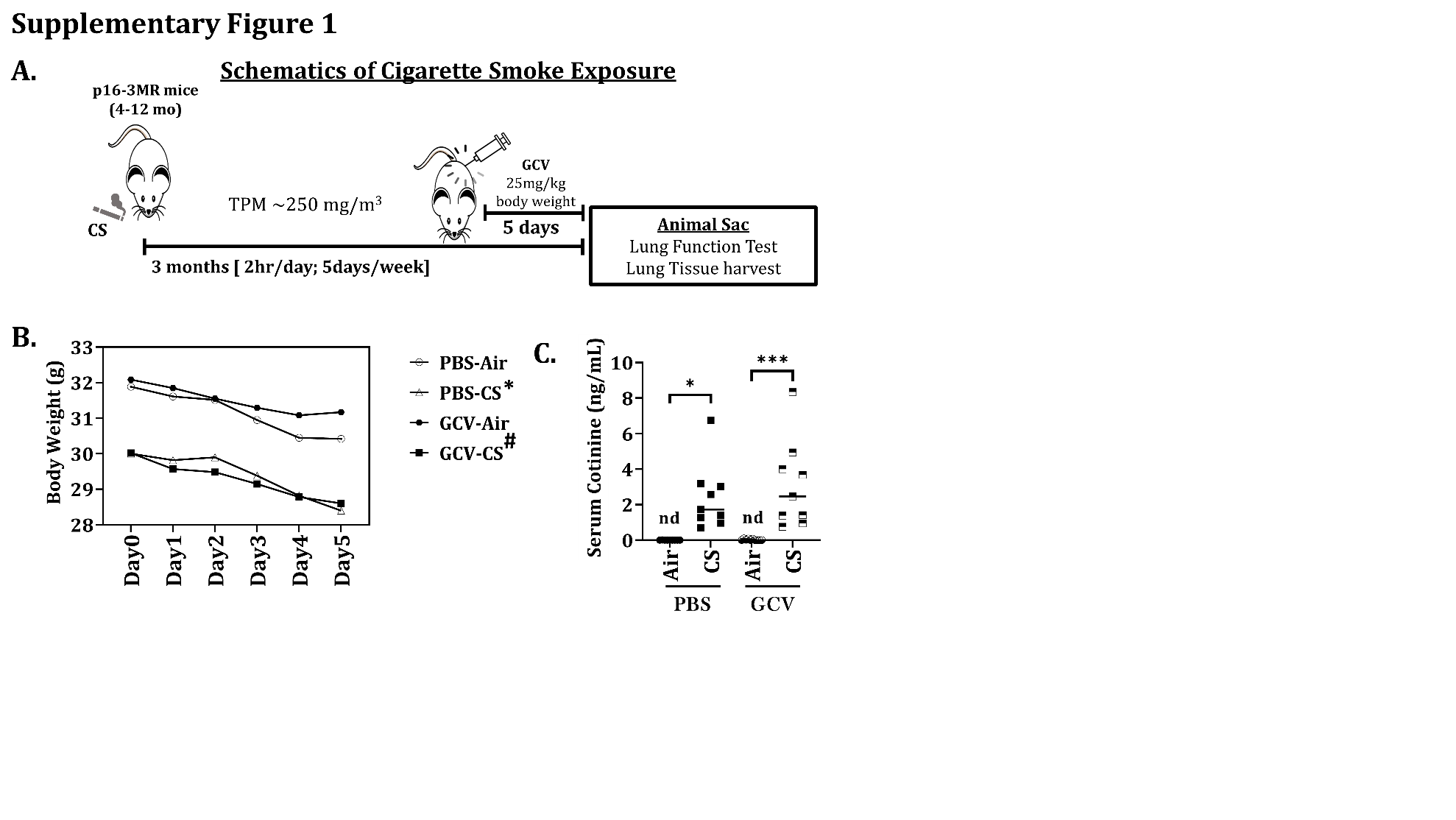
**

**Supplementary Figure 1: (A)** Schematics showing the dosage and duration of cigarette smoke (CS) exposure and GCV treatment in p16-3MR mice. Mouse exposures were performed 2 h per day, 5 days/wk for 3 months duration. **(B)** Changes observed in the body weight of air and CS-exposed p16-3MR over the span of 5-days of PBS or GCV administration. Data are shown as mean (n= 10-12/group). SE: **p* < 0.05 vs PBS-Air and # p<0.05 vs GCV-Air; as per unpaired t-test for pairwise comparisons. **(C)** The cotinine levels in plasma from PBS/GCV treated air/CS-exposed p16-3MR mice as determined using ELISA-based Cotinine assay. Data are shown as mean ± SEM (n = 10-12/groups). SE: **p* < 0.05, and ***p<0.001; as per One-way ANOVA for multiple comparisons.


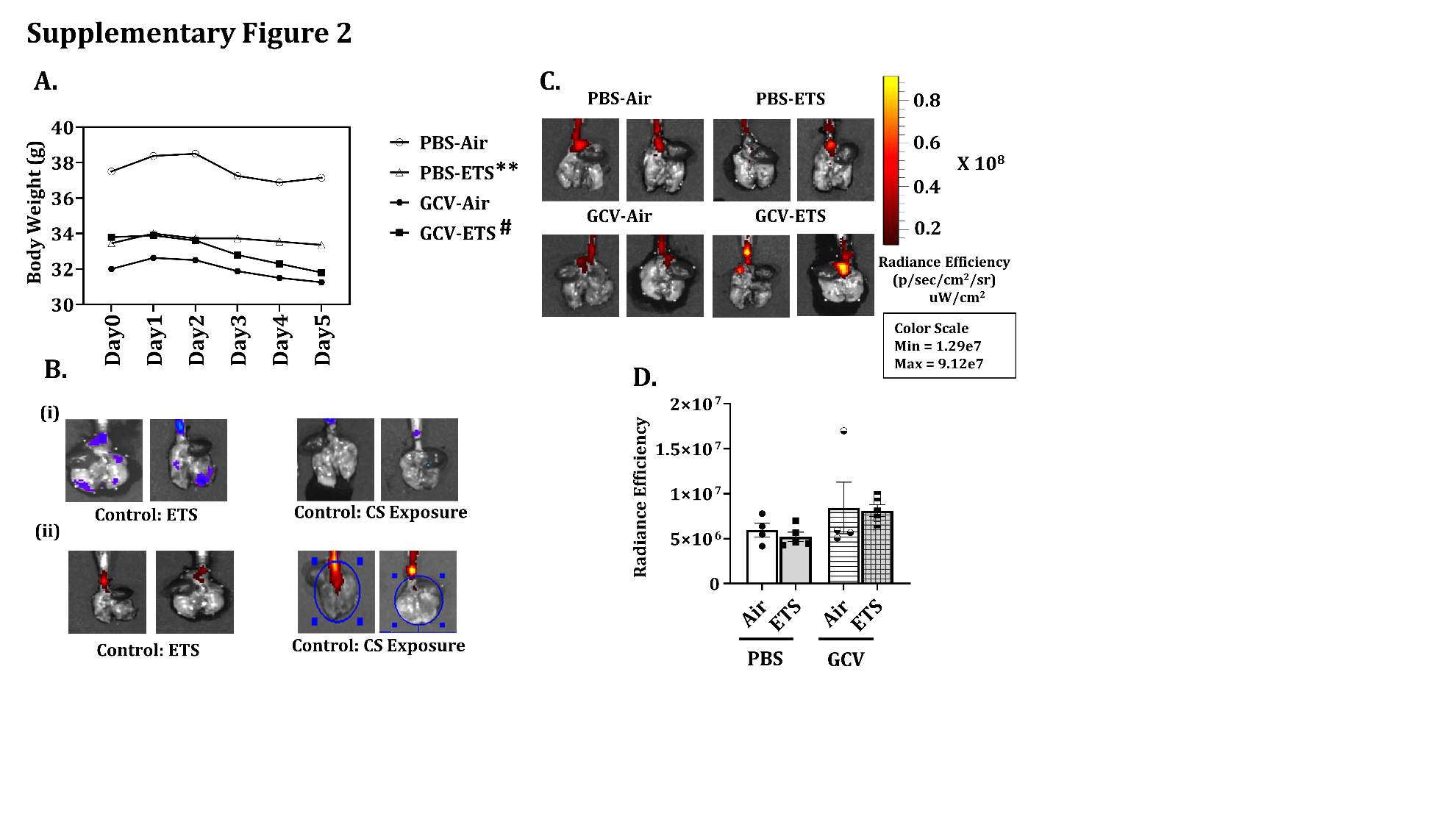


**Supplementary Figure 2: Body weight changes and alterations in the lung tissue fluorescence in PBS/GCV-treated air/ETS-exposed p16-3MR mice.** p16-3MR mice were subjected to chronic (6 months) ETS exposure, followed by 5-day treatment with GCV (25mg/kg body weight) or PBS (control). **(A)** Changes observed in the body weight of air and ETS-exposed p16-3MR over the span of 5-days of PBS or GCV administration. Data are shown as mean (n= 8-11/group). SE: **p<0.01 vs PBS-Air and # p<0.01 vs GCV-Air; as per paired t-test for pairwise comparisons. **(B)** C57BL/6J mouse lungs was used as the background control for calculating the relative (i) luminescence and (ii) fluorescence in p16-3MR mice lungs. The lung tissues fluorescence in air and ETS-exposed lungs measured using IVIS imaging. **(C)** Representative images of n = 4–5/group were provided and **(D)** the relative quantified counts (bottom) were plotted as mean ± SEM**.**

**
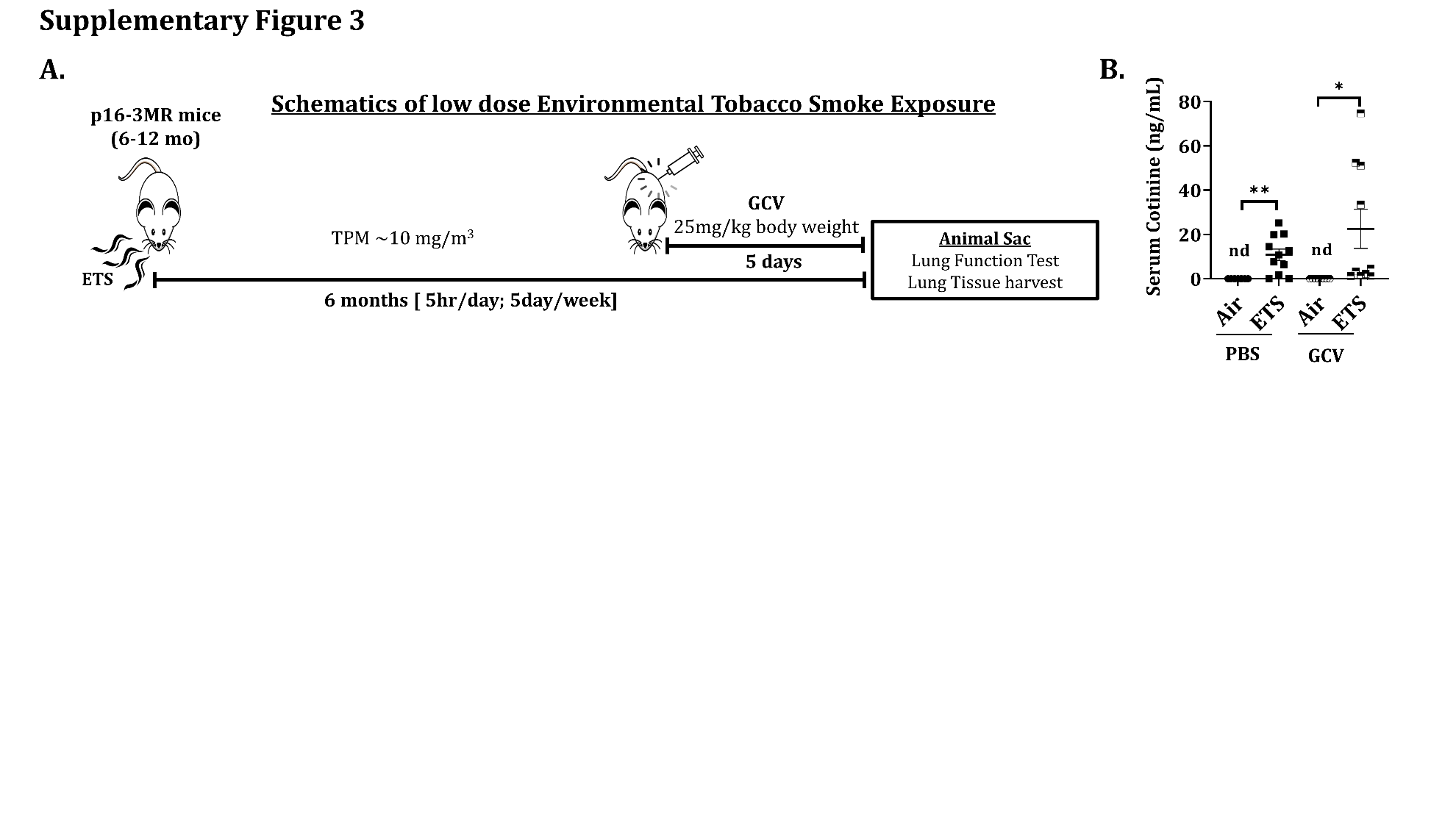
**

**Supplementary Figure 3: (A)** Schematics showing the dosage and duration of environmental tobacco smoke (ETS) exposure and GCV treatment in p16-3MR mice. Mouse exposures were performed 5 h per day, 5 days/wk for 6 months duration. **(B)** The cotinine levels in serum from PBS/GCV treated air/ETS-exposed p16-3MR mice as determined using ELISA-based Cotinine assay. Data are shown as mean ± SEM (n = 10-12/groups).

**
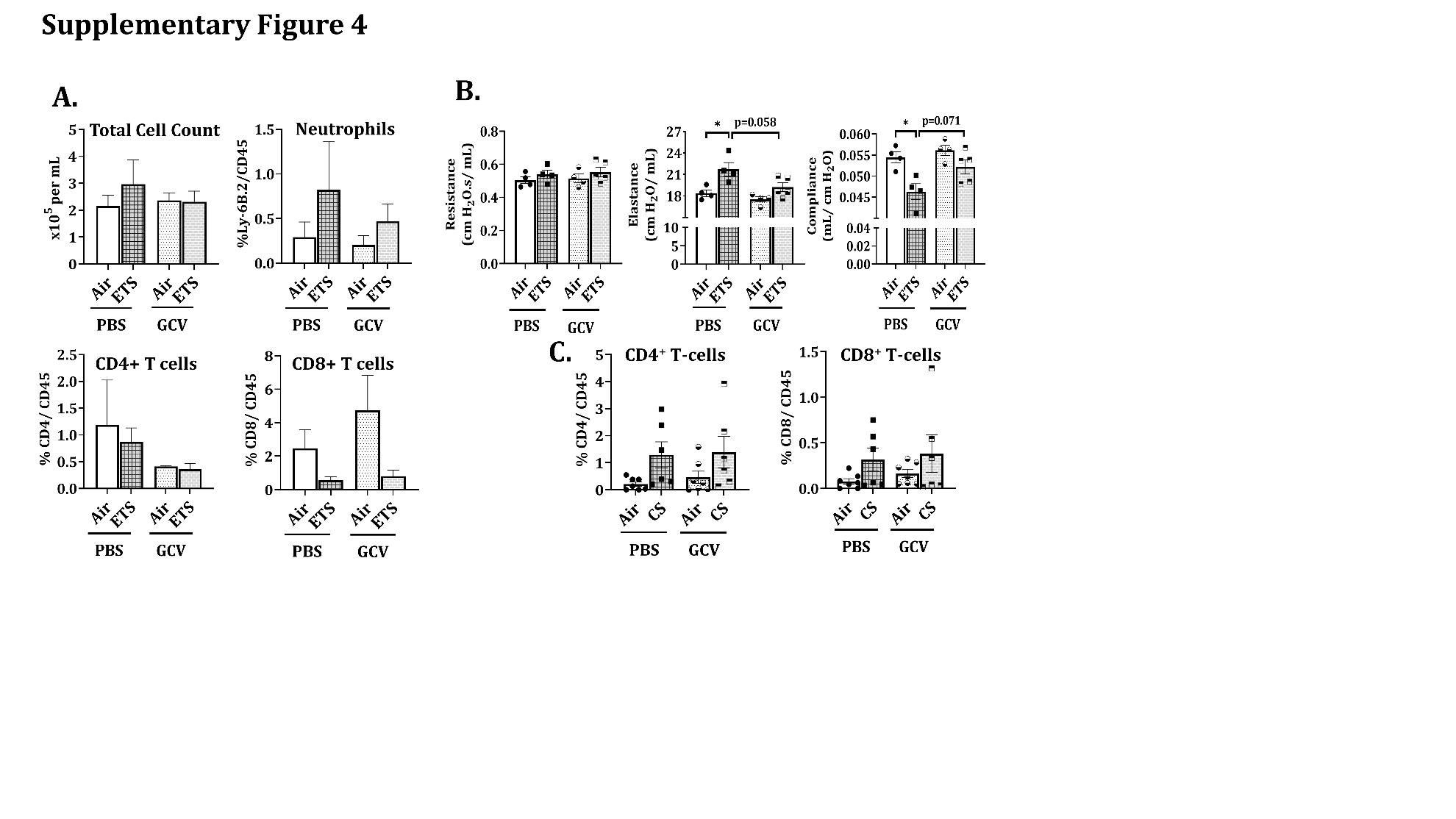
**

**Supplementary Figure 4: No change in the immune cell infiltration in BALF and lung function parameters of PBS/GCV-treated air/ETS-exposed p16-3MR mice.** p16-3MR mice were subjected to chronic (6 months) ETS exposure, followed by 5-day treatment with GCV (25 mg/kg body weight) or PBS (control). Flow cytometry was used to determine the immune cell population (neutrophils, CD4+ and CD8+ T-cells) in BALF from control and treated mice. **(A)** The changes in the total and immune cell population was plotted as bar graph. Data are shown as mean ± SEM (n = 4-6/group). **(B)** Lung function parameters of PBS/GCV treated air/ETS-exposed p16-3MR mice were determined using SCIREQ’s flexiVent system. *p<0.05 vs. Air. Data are shown as mean ± SEM (n = 4-5/groups). **(C)** BALF from the lungs of PBS/GCV-treated and air/CS exposed (3 months) p16-3MR mice was obtained. Total cell count was determined using AO/PI staining. Flow cytometry was used to determine the T cell population (CD4+ and CD8+ T-cells) in BALF from control and treated mice. The changes in the CD4+ and CD8+ T cell population was plotted as bar graph. Data are shown as mean ± SEM (n = 6-7/group); **p<0.01 and *** p<0.001 between groups, per one-way ANOVA for multiple comparisons.

**
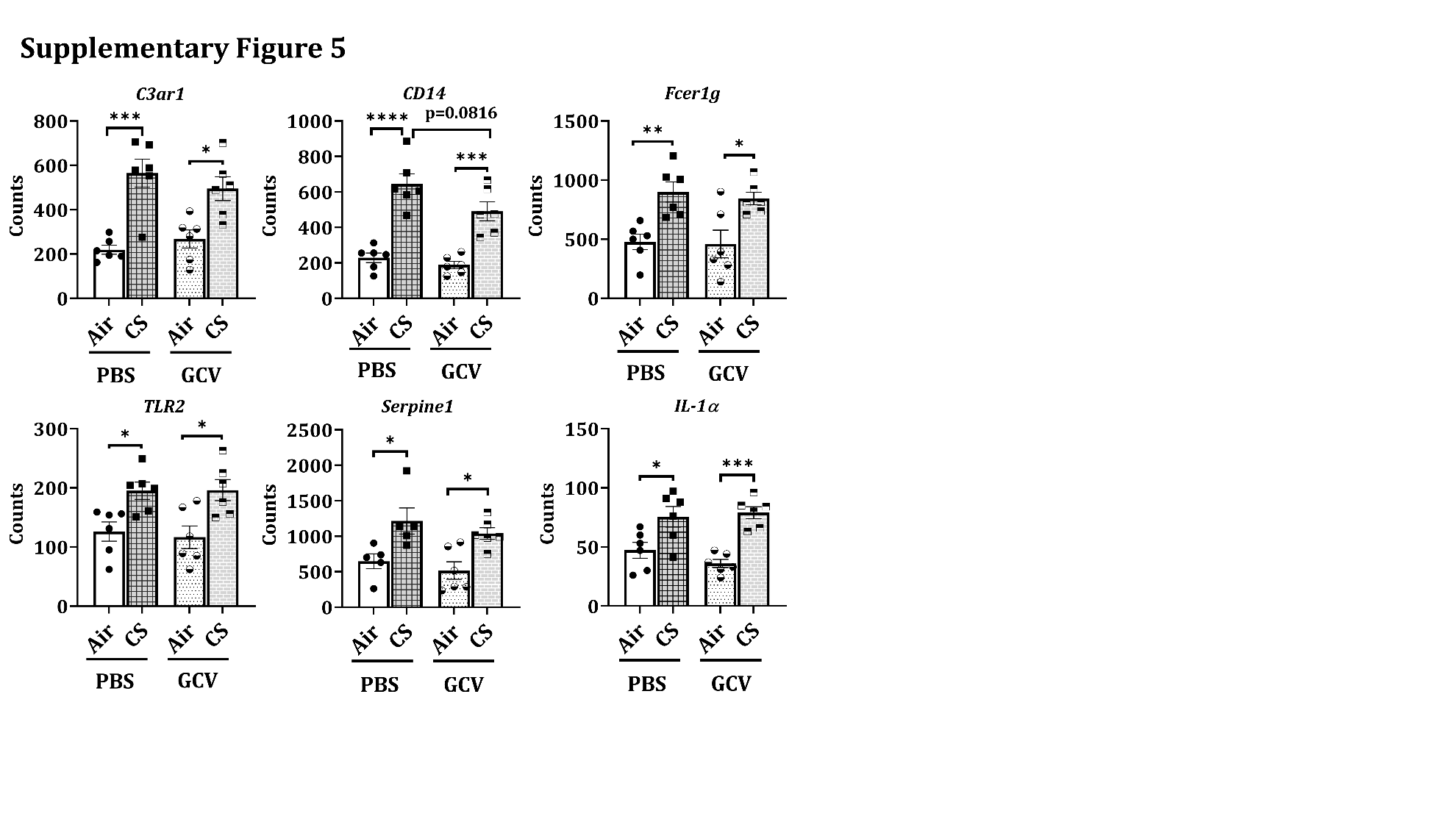
**

**Supplementary Figure 5: CS exposure alters the expression of complement system genes in lungs of p16-3MR mice.** Total RNA was isolated from the lungs of PBS/GCV treated and air/CS exposed (3-months) p16-3MR mice. Our customized NanoString panel (senescence) was used to screen the potential targets via nCounter SPRINT Profiler. Normalization of absolute RNA count and data analysis were done by nSolver software. The overview of selected dysregulated targets is shown as a bar graph. Data are shown as mean ± SEM (n = 6/group); *p < 0.05, **p< 0.01, ***p < 0.001 and **** p<0.0001 between groups, per one-way ANOVA for multiple comparisons.

**
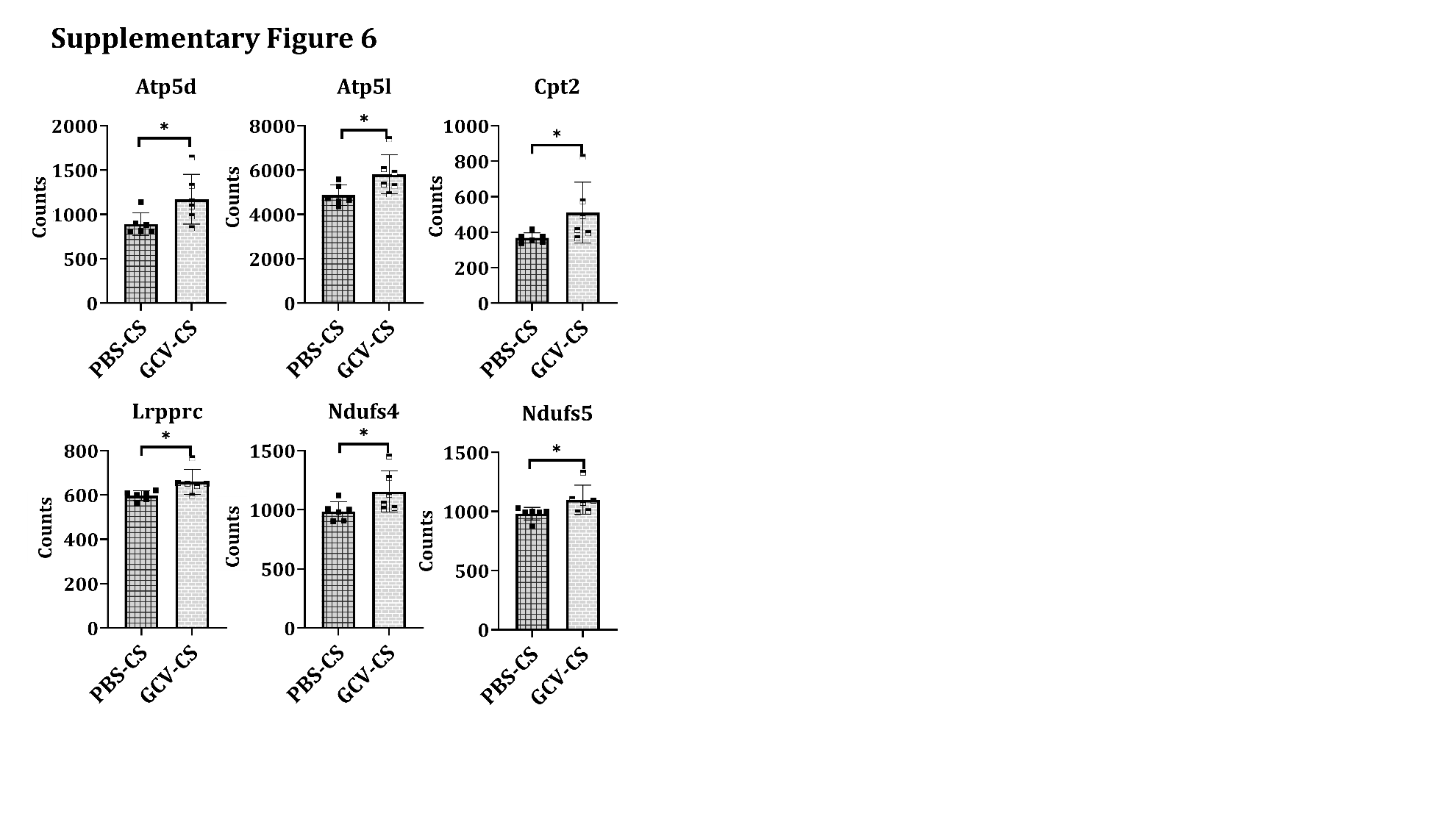
**

**Supplementary Figure 6:** **GCV treatment reverses the genes associated with MiDAS in CS-exposed p16-3MR mice.** Total RNA was isolated from the lungs of PBS/GCV treated and air/CS exposed (3-months) p16-3MR mice. Our customized NanoString panel (mitochondrial function) was used to screen the potential targets via nCounter SPRINT Profiler. Normalization of absolute RNA count and data analysis were done by nSolver software. The overview of all the selected gene transcription changes between PBS-CS versus GCV-CS are shown as bar graph. Data are shown as mean ± SEM (n = 6/group); *p < 0.05 vs PBS-CS, per Mann-Whitney’s test for pairwise comparisons.

**
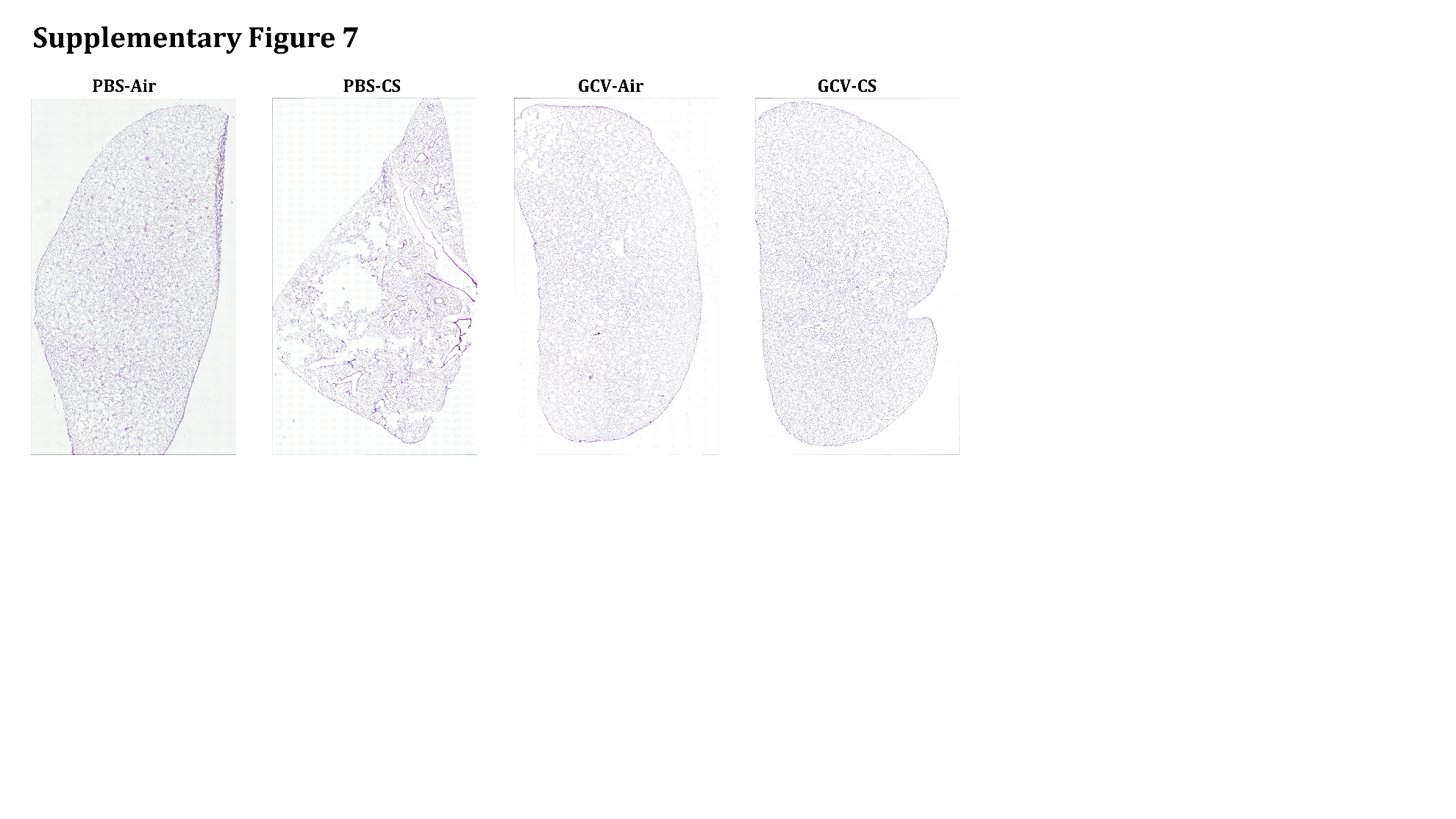
**

**Supplementary Figure 7:** Full scans of the H&E stained slides of PBS/GCV-treated Air/CS exposed mouse lungs.

**Supplementary Tables**

**Supplementary Table 1: Codeset Information for custom-designed Senescence Panel**

| **Identifier** | **Accession** | **Position** | **Target Sequence** | **Tm CP** | **Tm RP** | **NSID** |
| --- | --- | --- | --- | --- | --- | --- |
| Abcf1 | NM_013854.1 | 876-975 | GAGGTGTCTTCCCGCCAGGCAATGTTAGAAAATGCATCTGACATTAAGTTGGAAAAGTTCAGCATCTCCGCCCACGGCAAGGAGCTATTCGTCAATGCTG | 79 | 83 | NM_013854.1:875 |
| Abl1 | NM_009594.4 | 1379-1478 | ATTGCTCTGGGAGATTGCTACCTATGGCATGTCACCTTACCCGGGAATTGACCTGTCTCAGGTTTATGAGCTGCTGGAAAAAGACTACCGCATGGAGCGC | 82 | 82 | NM_009594.4:1378 |
| Akt1 | NM_001165894.1 | 899-998 | GCCATGAAGATCCTCAAGAAGGAGGTCATCGTCGCCAAGGATGAGGTTGCCCACACGCTTACTGAGAACCGTGTCCTGCAGAACTCTAGGCATCCCTTCC | 83 | 83 | NM_001165894.1:898 |
| Akt3 | NM_011785.3 | 773-872 | TTCTCTGAGGACCGCACACGTTTCTATGGTGCAGAAATTGTCTCTGCTTTGGACTATCTACATTCTGGAAAGATTGTGTACCGTGATCTCAAGTTGGAGA | 78 | 80 | NM_011785.3:772 |
| Aldh1a3 | NM_053080.3 | 126-225 | CCGCGCCCCATCCGCAACTTGGAGGTCAAGTTCACCAAGATATTTATCAACAACGACTGGCACGAATCCAAGAGTGGAAGAAAGTTTGCCACATATAACC | 80 | 83 | NM_053080.3:125 |
| Angel2 | NM_001199020.1 | 771-870 | GGGTTATCATTGTGAATATAAAATGAAAACAGGAAGGAAGCCCGACGGCTGTGCCATCTGCTTCAAACATTCCAGGTTCTCGCTCTTATCTGTGAACCCA | 82 | 79 | NM_001199020.1:770 |
| Anxa3 | NM_013470.2 | 1230-1329 | CGCCTCAAAATCTCTGCACACTGCTTTCATGCAGCACTCTAAAGTGCAAGCAAATGCAAGACAGAACCTGTCTGCCTGATAGGCATTGGCATCGTTCAGT | 83 | 84 | NM_013470.2:1229 |
| Anxa5 | NM_009673.2 | 1596-1695 | CCTTAGCGGTGAAATTGTGAACTCTTGGAAATGCTGTCAATCAAGCTTACTGCTCTAGCAGACCTGCAAAATTATGATGGTAGTATCCAAAACATTGGCG | 79 | 81 | NM_009673.2:1595 |
| Arg2 | NM_009705.2 | 250-349 | GGCTCTCCAGGTTGGGATGCCACCTAAAAGACTTTGGAGACTTGAGTTTTACTAATGTCCCACAAGATGATCCCTACAATAATCTGGTTGTGTATCCTCG | 83 | 79 | NM_009705.2:249 |
| Arid1a | NM_001080819.1 | 5194-5293 | TTAAAGGAGTATGAGGTAGGGGACCCAGGACAGAGAACATTACTAGACCCTGGGAGATTCACCAAGGTGTATAGTCCAGCCCATACAGAGGAAGAAGAGG | 89 | 89 | NM_001080819.1:5193 |
| Arl6ip6 | NM_022989.4 | 603-702 | TCCTCTTTCACCTGCTAGGTTCAAGAAACTGACTGGACATTCTTTCCACATGGGCTACAGCATGGCAATTTTGAATGGTATTGTCGCTGCTCTCACTGTG | 79 | 81 | NM_022989.4:602 |
| Arntl | NM_007489.3 | 1520-1619 | CGGGTGAAATCTATGGAGTACGTTTCTCGACACGCAATAGATGGGAAATTTGTTTTTGTAGATCAGAGGGCGACAGCTATTTTGGCGTATCTACCACAGG | 79 | 78 | NM_007489.3:1519 |
| Atm | NM_007499.2 | 5544-5643 | TTCCTCAAAGTGAAAGTCATGACATTTGGATAAAGACACTGACGTGTGCCTTTCTGGACAGTGGAGGCATAAACAGTGAAATTCTCCAGTTATTAAAGCC | 81 | 79 | NM_007499.2:5543 |
| Atr | NM_019864.1 | 4393-4492 | AGGAGTTGCTTTCTATTTATGACTGTAGAGAGATGCAGAGCAATGGCCCAGGTTACCAGTTGTGGAAAAGATTCCCTGAGCATGTCCGGGAAATATTAGA | 82 | 80 | NM_019864.1:4392 |
| Bcl2l12 | NM_029410.3 | 13-112 | GGCCCAGTGCGCAGGCGTGGGAAAGTTGAACTTCCGAAGTAAAAGTTTGTAGAAATTGTGAGAAAGCGTGTACGCCAGTGCGACGGGGACACAACCGCGA | 81 | 81 | NM_029410.3:12 |
| Bcl6 | NM_009744.3 | 186-285 | ACGTTGTCATCGTGGTGAGCCGTGAGCAGTTTAGAGCCCATAAGACAGTGCTCATGGCCTGCAGCGGCCTGTTCTACAGTATCTTCACTGACCAGTTGAA | 82 | 83 | NM_009744.3:185 |
| Bmi1 | NM_007552.4 | 3355-3454 | TCTCGAGGTTTTCATGGTGTTACCTAAGACAAAAGACATCTCACCCTCTATGATGGACTTACTTCTGAGAGTGCGTTTGAGGCACTTATGGCTTACTAAG | 78 | 80 | NM_007552.4:3354 |
| Bub1b | NM_009773.3 | 21-120 | CTGCGTCAAGAGACGTTAAATTTGAAACTTGGCGGCCCGTGCGTGATGGGGGCGTGAGGAGGCTATTCTGAGAAGGAATCGGGTGCGTGGTTTTGTTTAG | 83 | 81 | NM_009773.3:20 |
| C1qa | NM_007572.2 | 567-666 | CAGTGCCCGGCTTCTATTACTTCAACTTCCAAGTGATCTCCAAGTGGGACCTTTGTCTGTTTATCAAGTCTTCCTCCGGGGGCCAGCCCAGGGATTCCCT | 82 | 82 | NM_007572.2:566 |
| C1qb | NM_009777.2 | 866-965 | GTGCCAACAGCATCTTCACTGGCTTTCTGCTTTTCCCTGACATGGATGCGTAATCACGGGGTCAAATTACACCTATCCAACACCATCTTCCTGCCTCCCT | 81 | 82 | NM_009777.2:865 |
| C1qc | NM_007574.2 | 709-808 | CAGAGGGGCGATGAGGTGTGGCTATCAGTCAATGACTACAATGGCATGGTGGGCATAGAGGGCTCCAACAGCGTCTTCTCTGGTTTCCTACTGTTTCCCG | 82 | 83 | NM_007574.2:708 |
| C3ar1 | NM_009779.2 | 556-655 | ATAAGCCAATCTGGTGCCAGAATCATCGAAACGTGAGAACCGCCTTCGCCATCTGTGGATGTGTCTGGGTGGTAGCCTTTGTGATGTGTGTGCCCGTATT | 79 | 81 | NM_009779.2:555 |
| C5ar1 | NM_007577.3 | 596-695 | CATTGCTCCTCACCATTCCATCCTTCGTGTACCGGGAGGCATATAAGGACTTCTACTCAGAGCACACTGTATGTGGTATTAACTATGGTGGGGGTAGCTT | 83 | 82 | NM_007577.3:595 |
| Calb1 | NM_009788.4 | 344-443 | ATGGAAAAATAGGAATTGTAGAGTTGGCTCACGTCTTACCCACAGAAGAGAATTTCTTGCTGCTCTTTCGATGCCAGCAACTGAAGTCCTGCGAGGAATT | 82 | 79 | NM_009788.4:343 |
| Calr | NM_007591.3 | 552-651 | GCACCAAGAAGGTTCATGTCATCTTTAACTACAAGGGCAAGAATGTGCTGATCAACAAGGATATCCGGTGTAAGGATGATGAATTCACACACCTATACAC | 82 | 82 | NM_007591.3:551 |
| Casp1 | NM_009807.2 | 260-359 | GACAATAAATGGATTGTTGGATGAACTTTTAGAGAAGAGAGTGCTGAATCAGGAAGAAATGGATAAAATAAAACTTGCAAACATTACTGCTATGGACAAG | 78 | 81 | NM_009807.2:259 |
| Cav1 | NM_007616.3 | 1921-2020 | AACCCAAACTGAGGAATTTCACCTGTGTACCTGAGTCTCCAGAAAGCTGCCTGCCTGGGACACCCAAAAGCCTTTTACTTCCCAGCTCACATTACAGCTC | 79 | 81 | NM_007616.3:1920 |
| Ccna2 | NM_009828.2 | 1281-1380 | ACTGGATATACCCTGGAGAGTCTTAAGCCTTGTCTTGTGGACCTTCACCAGACCTACCTCAAAGCGCCACAACATGCCCAACAGTCAATACGGGAAAAGT | 83 | 79 | NM_009828.2:1280 |
| Ccnb1 | NM_172301.3 | 2137-2236 | TCATAGTAGCTCTTCCAGGGGTGTGCTTTGAATTCTGACAGCCAGATGGGTGTGGCTGCCACCATACCAAGGCGCCACTCCTGTCTTGTAATGCCACCTG | 84 | 86 | NM_172301.3:2136 |
| Ccnd1 | NM_007631.1 | 2001-2100 | GGAGGGGTTCTAATGGAATGGATGGGGATGTCCACACACGCATTCAGATGGCTGTACAACAGGTTGTAGGGCTGGTAGTATGAGGTGCTTGGGAAGTTTT | 82 | 81 | NM_007631.1:2000 |
| Ccne1 | NM_007633.2 | 886-985 | ATGATGATGAAGGCCCTTAAGTGGCGTCTAAGCCCTCTGACCATTGTGTCCTGGCTGAATGTCTATGTCCAAGTGGCCTATGTCAACGACACGGGTGAGG | 81 | 82 | NM_007633.2:885 |
| Ccr1 | NM_009912.4 | 1527-1626 | CTTCTGATTCAGACCATAGGTGTCAACCAAGGAAGGTCTAAGAAGAGAATGAGGAGACAGTATATAGCTCTCCAAGACTGATACTGACAGTTCTTACAGT | 82 | 83 | NM_009912.4:1526 |
| Cd14 | NM_009841.3 | 236-335 | CTAGACGAGGAAAGTTGTTCCTGCAACTTCTCAGATCCGAAGCCAGATTGGTCCAGCGCTTTCAATTGTTTGGGGGCGGCAGATGTGGAATTGTACGGCG | 81 | 82 | NM_009841.3:235 |
| Cd163 | NM_053094.2 | 3226-3325 | TCACGGCACTCTTGGTTTGTGGAGCCATTCTATTGGTCCTCCTCATTGTCTTCCTCCTGTGGACTCTGAAGCGACGACAGATTCAGCGACTTACAGTTTC | 79 | 82 | NM_053094.2:3225 |
| Cdc25c | NM_009860.2 | 43-142 | GAACCTCGGGCCACGTAGATGCAATTTTAACCCGAAGAATCGCTTCTGTTCCTCAGATCCCTTGTCATTCTGTGGAGTCTTCGCTTACGTCCAGTCCTGC | 80 | 80 | NM_009860.2:42 |
| Cdk2 | NM_016756.4 | 1469-1568 | TGCTGCCACTGTTTTGTGAACTAATGAACATGAGCAGAAGCCTAAGTTGGGACAGCTCAGAACCAAGCAAGAGGGGGCCGTTTTAATGAATTAGATTGAA | 83 | 83 | NM_016756.4:1468 |
| Cdk4 | NM_009870.3 | 495-594 | CCTCCACCGGGCCTGCCGGTTGAGACCATTAAGGATCTAATGCGTCAGTTTCTAAGCGGCCTGGATTTTCTTCATGCAAACTGCATTGTTCACCGGGACC | 84 | 81 | NM_009870.3:494 |
| Cdk6 | NM_009873.2 | 821-920 | TCCAAATCTGCTCAACCCATCGAGAAGTTTGTGACAGATATTGACGAACTAGGCAAAGACCTACTTCTGAAATGCCTGACGTTTAATCCAGCTAAAAGGA | 80 | 81 | NM_009873.2:820 |
| Cdkn1a | NM_007669.4 | 1671-1770 | AATACCGTGGGTGTCAAAGCACTTAGTGGGTCTGACTCCAGCCCCAAACATCCCTGTTTCTGTAACATCCTGGTCTGGACTGTCTACCCTTAGCCCGCAC | 82 | 82 | NM_007669.4:1670 |
| Cdkn1b | NM_009875.4 | 2017-2116 | AGGAAAAAAGGACACTTGTAGAGTAAGTGAAATGGATACTACATCTTTAAACAGCGTTTCTTCATTGCCTGTGTATGAGAAAAACTTGGAAGTGTGCCTG | 79 | 79 | NM_009875.4:2016 |
| Cdkn1c | NM_009876.3 | 1241-1340 | CTGGGACCTTTCGTTCATGTAGCAGGAACCGGAGATGGTTGCGTAGAGCAGCCCACGGTTTTGTGGAAATCTGAAAACTGTGCAATGTATTGAGAACACT | 82 | 80 | NM_009876.3:1240 |
| Cdkn2a | NM_001040654.1 | 567-666 | CCAATCCCAAGAGCAGAGCTAAATCCGGCCTCAGCCCGCCTTTTTCTTCTTAGCTTCACTTCTAGCGATGCTAGCGTGTCTAGCATGTGGCTTTAAAAAA | 83 | 78 | NM_001040654.1:566 |
| Cdkn2b | NM_007670.4 | 1041-1140 | AAATGGGAAACCTGGAGAGTAGATGAGAGGCACTGAAGTGGTTTTATGTACTGTCATGTGTGGGGATTCACCTGCAAAATGGCATTTATAGAAACTGAGA | 83 | 81 | NM_007670.4:1040 |
| Cdkn2c | NM_007671.2 | 266-365 | GCCAGGGGGGACCTAGAGCAACTTACTAGTTTGTTGCAAAATAATGTAAACGTCAACGCTCAAAATGGATTTGGGAGAACTGCGCTGCAGGTTATGAAAC | 82 | 82 | NM_007671.2:265 |
| Cdkn2d | NM_009878.3 | 713-812 | TCAGAACCTCATGGACATTCTGCAGGGGCACATGATGATCCCAATGTGACCCAAGGCCACTGTCTCCAGCCTTACTGGGTTACTTGTCAACAAAAGAGGA | 81 | 82 | NM_009878.3:712 |
| Chek1 | NM_007691.5 | 1221-1320 | TGGCAGCGCTTGGTCAAAAGGATGACACGATTCTTTACTAAATTGGATGCGGACAAATCTTACCAATGCCTGAAAGAGACCTTCGAGAAGTTGGGCTATC | 81 | 82 | NM_007691.5:1220 |
| Chek2 | NM_016681.3 | 791-890 | CCATAAAGATCATTAGCAAGCGGAGGTTTGCTCTTGGCTCATCGAGAGAAGCCGACACAGCTCCCAGTGTGGAAACTGAAATAGAAATTTTGAAGAAACT | 81 | 82 | NM_016681.3:790 |
| Cited2 | NM_010828.2 | 906-1005 | GTCCTTAGTGATAGAAATGGGTTTGGACCGCATCAAGGAGCTGCCCGAACTCTGGCTGGGCCAAAATGAGTTTGATTTTATGACGGACTTCGTGTGCAAG | 81 | 75 | NM_010828.2:905 |
| Creg1 | NM_011804.2 | 492-591 | GAAGAGGACTATGCAAGGGATTCGCTGTTTGTTCGACACCCTGAGATGAAGCACTGGCCTTCCAGCCATAACTGGTTCTTTGCTAAATTAAAAATAAGCC | 82 | 80 | NM_011804.2:491 |
| Cx3cl1 | NM_009142.3 | 126-225 | CGTTCTTCCATTTGTGTACTCTGCTGCCGGGTCAGCACCTCGGCATGACGAAATGCGAAATCATGTGCGACAAGATGACCTCACGAATCCCAGTGGCTTT | 82 | 83 | NM_009142.3:125 |
| Cxcl16 | NM_023158.6 | 680-779 | CGCAGGGTACTTTGGATCACATCCGAAAATACCTGAAAGCATTTCATCGTTGTCCATTCTTTATCAGGTTCCAGTTGCAGTCCAAAAGCGTGTGTGGGGG | 78 | 79 | NM_023158.6:679 |
| E2f1 | NM_007891.4 | 927-1026 | CTGTGGATTCTTCAGAGACATTTCAGATCTCCCTTAAGAGCAAACAAGGCCCCATTGATGTTTTCCTGTGCCCGGAGGAGAGTGCAGACGGGATTAGCCC | 80 | 82 | NM_007891.4:926 |
| E2f3 | NM_010093.3 | 1976-2075 | AGGCTCCCAAATCTTCCTGATGAGTCAGCAAGTGAGAAGATGTGCAATCAGGTGTCTCTCACCTGTCCTTCCCTCCCCTCCTTCCTCTCCTTTCCTTGCG | 79 | 81 | NM_010093.3:1975 |
| Egr1 | NM_007913.5 | 516-615 | CGGCAGCAGCGCCTTCAATCCTCAAGGGGAGCCGAGCGAACAACCCTATGAGCACCTGACCACAGAGTCCTTTTCTGACATCGCTCTGAATAATGAGAAG | 82 | 80 | NM_007913.5:515 |
| Elavl1 | NM_010485.3 | 4826-4925 | ATGCTTACTGTTGAGTGGCATTGATGATTTGACACATTCTTAGGGGAGCAACAGAACATGCATTTCCATCCTAAATAAGGACCCTCCCACTTCCACCTTT | 79 | 80 | NM_010485.3:4825 |
| Elp3 | NM_028811.2 | 2061-2160 | CATGACTCCCTGCCCTTTAACTACATGCACATTCACCCTGGAATTTAGAAAATGAGCTTTGCACTGGAAATCTCAGTGGCTGTGCCGTGTCTTGAAATGT | 80 | 82 | NM_028811.2:2060 |
| Eml1 | NM_001043335.1 | 2879-2978 | CTGTTTTGTCTACAGACTCTTAACAAACCTCAGGAAAACTGTCCCTCTACCAGTTACCTTAGTTGGGGAGCCAGTGCGTGTCACACCAGATAAGCGGTTG | 79 | 82 | NM_001043335.1:2878 |
| Ep300 | NM_177821.6 | 4306-4405 | TGGGACCTTTCTGGAGAATCGAGTGAATGACTTTCTGAGGCGACAAAATCACCCTGAATCAGGAGAGGTCACTGTTCGGGTTGTTCATGCTTCTGACAAA | 82 | 82 | NM_177821.6:4305 |
| Ets1 | NM_001038642.1 | 741-840 | GAAAGAGGATGTGAAACCATATCAGGTTAATGGAGCCAACCCTACCTACCCAGAATCCTGTTACACCTCGGATTACTTCATCAGCTACGGTATCGAGCAT | 80 | 78 | NM_001038642.1:740 |
| Ets2 | NM_011809.2 | 3285-3384 | TCAGTTTATGTACAGTGGAGCCACATGACATTGCCAAGTTTAACCTGAGTCTTGTCAGCCAACATGACGGTTTTACCTGGTTGAATCTAACAGTCATGGA | 79 | 78 | NM_011809.2:3284 |
| Fbxl16 | XM_001479823.1 | 2616-2715 | CCAAAACCTTTGCCTGCCCAAAGGGTTTGTGACCATGTTCTGTCAGGTCTCAAGAAATTTGGAAGTCCTAGGGGATCCTGGCATATAGGTGACAATACCG | 81 | 82 | XM_001479823.1:2615 |
| Fcer1g | NM_010185.4 | 265-364 | CTATAGCCAGCCGTGAGAAAGCAGATGCTGTCTACACGGGCCTGAACACCCGGAGCCAGGAGACATATGAGACTCTGAAGCATGAGAAACCACCCCAGTA | 83 | 82 | NM_010185.4:264 |
| Fcgr1 | NM_010186.5 | 186-285 | GAGACAGTTCCACACAATGGTTTATCAACGGAACAGCCGTTCAGATCTCCACGCCTAGTTATAGCATCCCAGAGGCCAGTTTTCAGGACAGTGGCGAATA | 81 | 84 | NM_010186.5:185 |
| Fcgr3 | NM_010188.5 | 1176-1275 | TCTGACCTCCACCATCCACCATGGCAGGTGCACACAATAAATTAAAATGTCATGTATATTTTTAAACAAGAGACAGGGGCAGGCTAAGGGTTGATGGCAT | 82 | 81 | NM_010188.5:1175 |
| Fcgr4 | NM_144559.1 | 609-708 | GGCGATCCAGGGTCTCCATCCATGTTTCCACCGTGGCATCAAATCACATTCTGCCTGCTGATAGGACTCTTGTTTGCAATAGACACAGTGCTGTATTTCT | 81 | 79 | NM_144559.1:608 |
| Foxo1 | NM_019739.2 | 2531-2630 | TTTCCTCAGACTTGGCAACAGCGGCAGCACTTTCCTGTGCAGGATGTTTGCCCAGCGTCCGCAGGTTTTGTGCTCCTGTAGATAAGGACTGTGCCATTGG | 81 | 82 | NM_019739.2:2530 |
| Foxo3 | NM_019740.2 | 2222-2321 | GATGCTGACGGGTTGGATTTTAACTTTGACTCCCTCATCTCCACACAGAACGTTGTTGGTTTGAATGTGGGGAACTTCACTGGTGCTAAGCAGGCCTCAT | 80 | 82 | NM_019740.2:2221 |
| Gadd45a | NM_007836.1 | 655-754 | ACGGTGATGGCATCCGAATGGAAATAACTGAACCAAATTGCACTGAAGTTTTGAAATACCTTTGTAGTTACTCAAGCAGTCACTCCCCACGCTGATGCAA | 83 | 80 | NM_007836.1:654 |
| Gadd45b | NM_008655.1 | 110-209 | CCTCCGACACTTCTGGTCGCACGGGAAGGTTTTTTTGCCTCTTGGGTTCGTATCTGGACTTGTACTTTGCTCTTGGGGATCTTCCGTGGGGGTCCGCTGT | 78 | 81 | NM_008655.1:109 |
| Glb1 | NM_009752.2 | 2131-2230 | GCCTTGGGGAGCACAGCTCTACTCTGGTTACACGGATCACCTTTGTTGTGCTAGAATGGAAGCTACATTTCTGGAATGTGTGTACCCTGCTGGTGACTTT | 82 | 81 | NM_009752.2:2130 |
| Gsk3b | NM_019827.3 | 2216-2315 | CCACTGATTACACGTCCAGTATAGATGTATGGTCTGCAGGCTGTGTGTTGGCTGAATTGTTGCTAGGACAACCAATATTTCCTGGGGACAGTGGTGTGGA | 81 | 81 | NM_019827.3:2215 |
| Gusb | NM_010368.1 | 284-383 | CCCTTCGGGACTTTATTGGCTGGGTGTGGTATGAACGGGAAGCAATCCTGCCACGGCGATGGACCCAAGATACCGACATGAGAGTGGTGTTGAGGATCAA | 85 | 86 | NM_010368.1:283 |
| Hprt | NM_013556.2 | 31-130 | TGCTGAGGCGGCGAGGGAGAGCGTTGGGCTTACCTCACTGCTTTCCGGAGCGGTAGCACCTCCTCCGCCGGCTTCCTCCTCAGACCGCTTTTTGCCGCGA | 82 | 82 | NM_013556.2:30 |
| Hras | NM_001130443.1 | 241-340 | GAGGCGTGGGAAAGAGTGCCCTGACCATCCAGCTGATCCAGAACCACTTTGTGGACGAGTATGATCCCACTATAGAGGACTCCTACCGGAAACAGGTGGT | 82 | 81 | NM_001130443.1:240 |
| Hsf1 | NM_008296.3 | 1210-1309 | CTTTGTCCCCAACTGCCTTCATTGACTCCATCCTTCGAGAGAGCGAGCCTACCCCTGCTGCCTCAAACACAGCCCCTATGGACACAACCGGAGCCCAAGC | 83 | 91 | NM_008296.3:1209 |
| Id1 | NM_010495.2 | 281-380 | CTGGTGCCCACCCTGCCCCAGAACCGCAAAGTGAGCAAGGTGGAGATCCTGCAGCATGTAATCGACTACATCAGGGACCTGCAGCTGGAGCTGAACTCGG | 80 | 83 | NM_010495.2:280 |
| Id2 | NM_010496.3 | 626-725 | AAGAAAGCGGAAGGAAAACTAAGGATGATCGTCTTGCCCAGGTGTCGTTCTCCGGCCTGGACTGTGATACCGTTATTTATGAGAGACTTTCAGTGCCCTT | 83 | 80 | NM_010496.3:625 |
| Ifng | NM_008337.1 | 96-195 | CTAGCTCTGAGACAATGAACGCTACACACTGCATCTTGGCTTTGCAGCTCTTCCTCATGGCTGTTTCTGGCTGTTACTGCCACGGCACAGTCATTGAAAG | 82 | 79 | NM_008337.1:95 |
| Igf1 | NM_001111274.1 | 419-518 | GGGCTTTTACTTCAACAAGCCCACAGGCTATGGCTCCAGCATTCGGAGGGCACCTCAGACAGGCATTGTGGATGAGTGTTGCTTCCGGAGCTGTGATCTG | 83 | 84 | NM_001111274.1:418 |
| Igf1r | NM_010513.2 | 3391-3490 | CGTATGAGAACTTCATGCATCTGATCATTGCTCTGCCGGTTGCCATCCTGCTGATCGTTGGGGGGCTGGTTATCATGCTGTATGTCTTCCATAGAAAGAG | 80 | 81 | NM_010513.2:3390 |
| Igfbp7 | NM_008048.2 | 616-715 | AAAAGGGATCACTCTGGAGTTCAGCGGACAGAACTCTTGCCTGGTGACCGGGAAAATCTGGCCATTCAGACCCGGGGTGGTCCAGAAAAGCATGAAGTAA | 85 | 86 | NM_008048.2:615 |
| Il1a | NM_010554.4 | 226-325 | ACCTCTGAAACGTCAAAGATGTCCAACTTCACCTTCAAGGAGAGCCGGGTGACAGTATCAGCAACGTCAAGCAACGGGAAGATTCTGAAGAAGAGACGGC | 79 | 81 | NM_010554.4:225 |
| Il6 | NM_031168.1 | 41-140 | CTCTCTGCAAGAGACTTCCATCCAGTTGCCTTCTTGGGACTGATGCTGGTGACAACCACGGCCTTCCCTACTTCACAAGTCCGGAGAGGAGACTTCACAG | 82 | 82 | NM_031168.1:40 |
| Ing1 | NM_011919.4 | 1707-1806 | CAGTGACACAGCCACCAGTGTGTTTATGGTATCGCTGCCTTCGTGGAAGTCCGAGGGCAGTCAGATGAGTATTTTAGAGAATGTTAGCCGTGCCTCCTCT | 83 | 82 | NM_011919.4:1706 |
| Irf3 | NM_016849.4 | 527-626 | GAAAGAAGTGTTGCGGTTAGCTGCTGACAATAGCAAGGACCCTTATGACCCTCATAAAGTGTATGAGTTTGTGACTCCAGGGGCGCGGGACTTCGTACAT | 81 | 82 | NM_016849.4:526 |
| Irf5 | NM_001252382.1 | 491-590 | CCCTTAACAAAAGCCGTGACTTCCAGCTGTTCTATGATGGCCCTCGGGACATGCCACCTCAGCCGTACAAGATCTACGAGGTCTGCTCCAACGGCCCTGC | 82 | 82 | NM_001252382.1:490 |
| Irf7 | NM_016850.2 | 706-805 | CGCTGTGCACTCCACAGCACAGGGCGTTTTATCTTGCGCCAAGACAATTCAGGGGATCCAGTTGATCCGCATAAGGTGTACGAACTTAGCCGGGAGCTTG | 81 | 82 | NM_016850.2:705 |
| Jakmip3 | NM_028708.2 | 5291-5390 | CCCTGTGTGCAGCACATGGAACAGCATAAGCGTTTTCGTTTGTCTAAACTAGAAATCCACGTAAACTGCAGAGTCCTTTGTGAACAGCCGTGTGGTGTCT | 78 | 83 | NM_028708.2:5290 |
| Kat6a | NM_001364449.1 | 3761-3860 | CACTCCTGTCCTAAAGCCAGTATCTCTCTTGCGAAAATGTGATGTGAACAGTGCTTCACTTGAGCCAGATACCTCTACACCTATGAAAAAGAAAAAGGGA | 81 | 82 | NM_001364449.1:3760 |
| Kras | NM_021284.5 | 2621-2720 | GGGCCACTACAACAATTCAATCTCAATCCTTTGGACTTCATTCCTGCTGCCCAGGCCACTGGTGCCTCAGTAGGAATTTTCAAAATTAGTGTGAACAGAC | 80 | 82 | NM_021284.5:2620 |
| Ldha | NM_010699.1 | 256-355 | CAGAACAAGATTACAGTTGTTGGGGTTGGTGCTGTTGGCATGGCTTGTGCCATCAGTATCTTAATGAAGGACTTGGCGGATGAGCTTGCCCTTGTTGACG | 80 | 78 | NM_010699.1:255 |
| Lmna | NM_001002011.2 | 1612-1711 | CCAGTCCATGGGCAACTGGCAGATCAGGCGTCAGAATGGTGACGATCCTTTGATGACCTATCGCTTCCCACCGAAGTTCACCCTAAAGGCTGGGCAGGTG | 86 | 84 | NM_001002011.2:1611 |
| Lmnb1 | NM_010721.2 | 806-905 | ATCAGATTGCCCAGCTAGAAGCATCCTTATCTGCCGCCAAAAAGCAGTTAGCAGATGAAACTTTACTTAAAGTGGATTTGGAGAATCGCTGTCAGAGCCT | 83 | 80 | NM_010721.2:805 |
| Lmnb2 | NM_010722.5 | 3107-3206 | CTTGTATTTGAGGAGCAGGTCTAGAGTAATCTGCAGGTCCTCAGGGGTCTGTTTCTGGCCCTGGCTAGGTGAGATGGTTACCGGAGTGGCACTGCTGCTG | 82 | 82 | NM_010722.5:3106 |
| Lsm5 | NM_025520.3 | 65-164 | CTCCTGCCACTAGAGCTTGTGGACAAATGTATAGGGTCAAGAATTCACATTGTGATGAAGAGTGATAAAGAAATTGTCGGGACACTTCTAGGATTTGATG | 83 | 78 | NM_025520.3:64 |
| Ltf | NM_008522.3 | 2546-2645 | TGCGTATGGGTGACCATGTATGCCTCAACATAAGTTTGCACATGACTGTGTGCACTCCTTGCACTGATGTTTGCATAGGACTGTGTACTTGCACTAATGC | 82 | 83 | NM_008522.3:2545 |
| Map2k1 | NM_008927.3 | 1696-1795 | CTTTGTGCTTGGGGCTATTTGTCTGTTCATCAAACACATGCCAGGCTGAACTACAGTGAAACCCTAGTGACCTGGGTGGTCGTTCTTACTGATGTTTGCA | 79 | 81 | NM_008927.3:1695 |
| Map2k3 | NM_008928.4 | 1116-1215 | TGGAGCTGATGGAACACCCATTCTTCACCTTGCACAAAACTAAGAAGACAGACATTGCTGCCTTTGTGAAGGAGATCCTGGGAGAGGATTCATAGGGACT | 81 | 83 | NM_008928.4:1115 |
| Map2k6 | NM_011943.2 | 321-420 | GGGCCTTAAGATTCCAAAAGAAGCGTTTGAACAGCCTCAGACCAGTTCCACGCCGCCTCGGGATTTAGACTCCAAGGCTTGCATATCTATTGGAAACCAG | 83 | 83 | NM_011943.2:320 |
| Mapk14 | NM_011951.2 | 1421-1520 | GAAGACCTTCTCATGGGAACTCTCCAAATACCATTCAAGTGCCTCTTGTTGAAAGATTCCTTCATGGTGGAAGGGGGTGCATGTATGTGTTAGTGTTTGT | 80 | 78 | NM_011951.2:1420 |
| Mdm2 | NM_010786.4 | 1665-1764 | GTCATGTTTCACGTGTGCAAAGAAGCTAAAAAAAAGAAACAAGCCCTGCCCAGTGTGCAGACAGCCAATCCAAATGATTGTGCTAACTTACTTCAACTAG | 82 | 80 | NM_010786.4:1664 |
| Morc3 | NM_001045529.1 | 2211-2310 | ACAGGTGTTACAGCAAAGACTATTGGAGATGAACGACAAGTGCGTGAAGAAGGAGAAGTGCCACCAGTCTACTGAAACTGACGCAGTGTTTCTACTTGAC | 79 | 82 | NM_001045529.1:2210 |
| Mrpl43 | NM_053164.3 | 495-594 | GGATTCTGCTCCAGCTTCGATGCAAGCACAGTAAAGAACCAGGCGCTTTGGACCGTGAGAGGGATAGGATTGGTTCCTCTTTTGGATTTCAAGCTCAGGC | 82 | 81 | NM_053164.3:494 |
| Mtor | NM_020009.2 | 2433-2532 | CATGGAGCCTATCCTGAAGGCTTTAATTTTGAAACTGAAAGATCCAGACCCTGACCCAAACCCGGGCGTGATCAATAACGTGTTGGCCACTATAGGAGAA | 80 | 84 | NM_020009.2:2432 |
| Myc | NM_010849.4 | 631-730 | CCCTCAACGTGAACTTCACCAACAGGAACTATGACCTCGACTACGACTCCGTACAGCCCTATTTCATCTGCGACGAGGAAGAGAATTTCTATCACCAGCA | 83 | 78 | NM_010849.4:630 |
| Nbn | NM_013752.3 | 1001-1100 | AAGGAATGGTCTCAGACCTATTCCTGAAGCGGAGATTGGATTGGCTGTTATTTTTATGACTACAGAGAATTACTGTAATCCGCAGGGCCAGCCTTGTACA | 83 | 79 | NM_013752.3:1000 |
| Ndufb11 | NM_019435.4 | 79-178 | CCCAGACTAACAGAAATGATAACGTTGATTCGGTAGCGACACGGCGTGGGAGGCAAAATAGAGTCCGCACCTCGCCGAACTCGTAAACAAACTTAGGCCG | 82 | 82 | NM_019435.4:78 |
| Nfatc1 | NM_016791.4 | 1571-1670 | ATCCCGTTGCTTCCAGAAAATAACATGCGAGCCATCATCGACTGTGCTGGGATCCTGAAGCTCAGAAACTCTGATATTGAGCTGAGGAAAGGGGAGACAG | 83 | 84 | NM_016791.4:1570 |
| Nfatc2 | NM_001037177.1 | 1560-1659 | GGATCCTTAAGCCGCACGCCTTCTACCAAGTACACAGGATCACTGGGAAAACGGTCACCACCACGAGCTATGAGAAGATCGTAGGCAACACCAAGGTCCT | 82 | 84 | NM_001037177.1:1559 |
| Nfkb1 | NM_008689.2 | 2126-2225 | GTCTTACACTTAGCCATCATCCACCTCCACGCTCAGCTTGTGAGGGATCTGCTGGAAGTCACATCTGGTTTGATCTCTGATGACATCATCAACATGAGAA | 83 | 81 | NM_008689.2:2125 |
| Nox4 | NM_015760.4 | 501-600 | TCCCAGAAAGCTTCTCTTCACAACCATTCCTGGTCTGACGGGTGTCTGCATGGTGGTGGTATTGTTCCTCATGGTTACAGCTTCTACCTACGCAATAAGA | 82 | 79 | NM_015760.4:500 |
| Opa1 | NM_001199177.1 | 2846-2945 | CTACCAGAGGCATTTTATAGATTCTGAGCTGGAATGCAATGACGTGGTCCTGTTTTGGCGAATACAGCGCATGCTCGCTATCACTGCCAATACATTAAGG | 81 | 80 | NM_001199177.1:2845 |
| Panx1 | NM_019482.2 | 1323-1422 | CTTCATCCCATTCCGGCAGAAAACGGACATTCTCAAAGTGTATGAAATCCTGCCCACCTTCGATGTTCTACATTTCAAGTCTGAAGGCTACAATGACTTG | 78 | 78 | NM_019482.2:1322 |
| Pawr | NM_054056.2 | 1211-1310 | GGTGTGGAAAGCCTGCTTTTACACTACTGATGAATGTCATGGCTAAGGCTGAGCTGAGACGCCTGTGATTCACTGCGTTGTTGAGAGGACTGTATATTTA | 80 | 80 | NM_054056.2:1210 |
| Pcna | NM_011045.2 | 591-690 | AGACCTTAGCCACATTGGAGATGCTGTTGTGATATCCTGTGCAAAGAATGGGGTGAAGTTTTCTGCAAGTGGAGAGCTTGGCAATGGGAACATTAAGTTG | 82 | 81 | NM_011045.2:590 |
| Pdcd6 | NM_011051.3 | 519-618 | CTCATCCGAAAATTTGACAGGCAAGGACGGGGCCAGATCGCATTTGATGACTTCATCCAGGGCTGCATCGTCTTGCAGAGGTTGACAGACATATTCAGAC | 82 | 82 | NM_011051.3:518 |
| Phf3 | NM_001081080.1 | 3815-3914 | TTACACTTTGCTGTTTGCGTACTTCAGTAGCAGAAAGCGCTATGGTGTGGCTGCTAACAACATGAAGCAGGTTAAAGACATGTACCTCATTCCATTGGGC | 79 | 82 | NM_001081080.1:3814 |
| Pik3ca | NM_008839.1 | 1256-1355 | ACTGTCCGTTGGCCTGGGGAAACATAAACTTGTTTGATTATACAGACACCCTAGTGTCCGGGAAAATGGCTTTGAATCTCTGGCCTGTACCGCATGGGTT | 81 | 82 | NM_008839.1:1255 |
| Pla2r1 | NM_008867.2 | 859-958 | TATGAAGGTGTTCTGTGATGCTACGTGGCAAAGGAATGGCAGTTCACGCATTTGCTACCAGTTCAACCTGCTTTCGTCTCTGTCTTGGAACCAGGCCCAT | 82 | 80 | NM_008867.2:858 |
| Plau | NM_008873.2 | 1951-2050 | CAAGTCTAGGTATTTCCCTAACTCCAGACTGTGATGCGGGGCCATTTGGTCTTCCATGTGATGCTCCACGTGAATGTATCATTCCCGGGCGTGACCCGTG | 82 | 82 | NM_008873.2:1950 |
| Polr1b | NM_009086.2 | 2796-2895 | TGCCTTTCACTGAGAGTGGCATGATGCCGGACATTCTGTTTAATCCTCACGGGTTTCCCTCCCGTATGACCATAGGTATGTTAATCGAGAGCATGGCTGG | 81 | 83 | NM_009086.2:2795 |
| Polrmt | NM_172551.3 | 3651-3750 | TGACCCACGCCATGCGGTCCCTAAACCTGTGCATAATGTAAATAAATGAAAGTTTGTGTCTACCTATGCCAGCCGCCACTTTCTGGCGGTGCGCAAACCC | 82 | 83 | NM_172551.3:3650 |
| Pot1a | NM_133931.4 | 2237-2336 | TCACCATCGGAACAGAGCGACGAATTTGCTACCAGATTTTTGATACTACAGTTGCAGAAAATGTTGTCTAGTACTGCCATTGAGTGCAGTGTGTATGGGA | 78 | 80 | NM_133931.4:2236 |
| Prkcd | NM_011103.2 | 1266-1365 | AGGACCACCTGTTCTTCGTGATGGAGTTTCTCAATGGGGGTGACCTGATGTTCCACATTCAGGACAAAGGCCGCTTCGAACTCTACCGGGCTACGTTTTA | 82 | 81 | NM_011103.2:1265 |
| Pten | NM_008960.2 | 5161-5260 | CAGCCTTACCCCGATTCAGCCTCTTCAGATACTCTTGTGCTGTGCAGCAGTGGCTCTGTGTGTAAATGCTATGCACTGAGGATACACAAATATGACGTGT | 81 | 79 | NM_008960.2:5160 |
| Rap1a | NM_145541.4 | 696-795 | CAGCTCTGAGCCAGATTACAGAAATGAAGAACTGTTGCCTAATTGGAAAGTGCCAGCATTCCAGACTTCAAAAACGAAATCTGAAGAGGCTTCTCCTGTT | 83 | 81 | NM_145541.4:695 |
| Rb1 | NM_009029.2 | 1591-1690 | CTGGCCTGTGCTCTTGAAGTTGTAATGGCTACGTATAGCAGAAGTACATTGCAGCATCTTGATTCTGGAACAGATTTGTCCTTCCCGTGGATTCTGAACG | 80 | 78 | NM_009029.2:1590 |
| Rbl1 | NM_001139516.1 | 1193-1292 | ACTGGACGGCGGTATTTACAAGAAAAAGAGGCAGTCACCACGCCTGTAGCTTCAGCCACTCAAAGTGTAAGCCGGTTACAGAGCATCGTCGCTGGATTAA | 82 | 81 | NM_001139516.1:1192 |
| Rbl2 | NM_011250.4 | 1055-1154 | AGAAAATCTTACTGGCTTCCTGGAGCCCGGAAACTTTGGAGAGAGTTTTAAGGCCGTTAATAAGGCATATGAAGAATACGTGTTAGCCGCTGGGAATCTG | 81 | 83 | NM_011250.4:1054 |
| Rela | NM_009045.4 | 1047-1146 | GGCACGAGGCTCCTTTTCTCAAGCTGATGTGCATCGGCAAGTGGCCATTGTGTTCCGGACTCCTCCGTACGCCGACCCCAGCCTCCAGGCTCCTGTTCGA | 85 | 87 | NM_009045.4:1046 |
| Rnf144b | NM_146042.4 | 2535-2634 | GAGCATATTTTTCCTCTAGCAGTATACAGTTGCCATTAGCAAAGCAGCCCGGTGCTTCATAAGAACTGTGTGTGTTCCACGTTAACAGAGACCTCTCCTG | 78 | 81 | NM_146042.4:2534 |
| Rplp0 | NM_007475.5 | 496-595 | TCAGAACACTGGTCTAGGACCCGAGAAGACCTCCTTCTTCCAGGCTTTGGGCATCACCACGAAAATCTCCAGAGGCACCATTGAAATTCTGAGTGATGTG | 84 | 83 | NM_007475.5:495 |
| Scn2b | NM_001014761.2 | 1266-1365 | AGGTGGGGTCCTACCATTTGTGAAAGCACTATTTAAAGGGATGACTGTGCATTTCCAGGGTACTCAAGGAAAAAGGAAAGATAGGGCTGAGTCGTTTCTG | 82 | 82 | NM_001014761.2:1265 |
| Serpinb2 | NM_001174170.1 | 716-815 | TGGGCTTTATCCTTTCCGTGTGAACTCGCATGAGAGCATACCTGTCCAGATGATGTTCCTCCATGCAAAGCTGAACATTGGATACATAAAGGACCTGAAG | 82 | 82 | NM_001174170.1:715 |
| Serpine1 | NM_008871.2 | 1823-1922 | AGGGGCAACGGATAGACAGATCAAATGGTGGCCCAATAGCGAGCCTTCTCCCTGCTCCCTCCCTTGACACAGCTTGCTTATGTTATTTCAGAGTGTAGGT | 84 | 79 | NM_008871.2:1822 |
| Sirt1 | NM_019812.2 | 844-943 | GGATTCCTGACTTCAGATCAAGAGACGGTATCTATGCTCGCCTTGCGGTGGACTTCCCAGACCTCCCAGACCCTCAAGCCATGTTTGATATTGAGTATTT | 83 | 79 | NM_019812.2:843 |
| Sirt3 | NM_001127351.1 | 806-905 | CCAGCTTGTCTGAAGCAGTACAGAAATCAGTGCCCCGACTGCTCATCAATCGAGACTTGGTGGGGCCGTTCGTTCTGAGTCCTCGAAGGAAAGATGTGGT | 82 | 83 | NM_001127351.1:805 |
| Sirt6 | XM_006513863.2 | 136-235 | TTCGAGAATGCTCGGCCCTCGAAGACCCACATGGCCCTGGTTCAGCTAGAACGCATGGGCTTCCTCAGCTTCCTGGTCAGCCAGAACGTAGACGGGCTGC | 86 | 85 | XM_006513863.2:135 |
| Smad2 | NM_010754.4 | 881-980 | TCACAGCTTGGATTTGCAGCCAGTTACTTACTCGGAACCTGCATTCTGGTGTTCAATCGCATACTATGAACTAAACCAGAGGGTTGGAGAGACCTTCCAT | 81 | 82 | NM_010754.4:880 |
| Snap23 | NM_009222.3 | 853-952 | CCGCTGAGTCATTTCTTATTCAGAGTTTGAAAGGCTGTGAGAAGGAGCAGAGGAGGAATGCCTCGCTACTTTCTTACTTTGTCTTTAGGGGGTAGGTTCT | 79 | 79 | NM_009222.3:852 |
| Sod1 | NM_011434.1 | 407-506 | TGTGTCCATTGAAGATCGTGTGATCTCACTCTCAGGAGAGCATTCCATCATTGGCCGTACAATGGTGGTCCATGAGAAACAAGATGACTTGGGCAAAGGT | 80 | 81 | NM_011434.1:406 |
| Tbx2 | NM_009324.2 | 2811-2910 | AGTCTATCCAGAACACAAGTCACTCTGGAATCCCTGGATTTGATGCAAATTTCTGTTCGAGAGTCAACTACCAGGGCACCTCCTTCTTTCAACACTGTGG | 82 | 82 | NM_009324.2:2810 |
| Tbx3 | NM_198052.1 | 2036-2135 | CGCGGTTCCACATCGTCAGAGCCAACGATATCCTGAAACTGCCTTACAGTACTTTTCGAACCTACCTGTTCCCGGAAACAGAATTCATCGCCGTTACTGC | 82 | 79 | NM_198052.1:2035 |
| Terf1 | NM_009352.3 | 369-468 | TGTCAGTTTTTGACAAGAGTTGCATCTGGAAAGGCCCTTGATGCACAGTTTGAAGTTGATGAGCGTATTACACCCTTGGAATCAGCCCTGATGATTTGGA | 79 | 80 | NM_009352.3:368 |
| Terf2 | NM_001083118.2 | 773-872 | AAAAGAACTTGGCCCACCCTGTTATCCAGAACTTTTCCTATGAGGTCTTCCAGCAGAAGATGCTGCGTTTCCTAGAGAGCCACCTGGATGACACGGAGCC | 79 | 83 | NM_001083118.2:772 |
| Tert | NM_009354.1 | 2163-2262 | CAGGGTAAGCTGGTGGAGGTTGTTGCCAATATGATCAGGCACTCGGAGAGCACGTACTGTATCCGCCAGTATGCAGTGGTCCGGAGAGATAGCCAAGGCC | 83 | 82 | NM_009354.1:2162 |
| Tfam | NM_009360.4 | 791-890 | CATCCGTCGAAGTGTGAAACGATCCGGAGACATCTCTGAGCATTAAGATGGAAGACGGAGTTGTCATTGGGATTAGGCCCAAGAAACCAGTTAGGTCTCA | 81 | 83 | NM_009360.4:790 |
| Tfb1m | NM_146074.1 | 249-348 | GGTTGAAAAGGACACTCGCTTTATCCCAGGGTTACAGATGCTCTCTGATGCAGCACCTGGAAAACTCCGAATTGTTCATGGAGATGTGCTGACATACAAG | 82 | 82 | NM_146074.1:248 |
| Tfb2m | NM_008249.4 | 2158-2257 | TTACGCTTAGAAAATAAATTGATTTGGGCAGACATGGTGGCATAAGTCCCAGGCCTTGTGAGGTGGAGGGCCCATCAGTTTAATGCCACTTTCAACTGTA | 79 | 84 | NM_008249.4:2157 |
| Tgfb1 | NM_011577.1 | 1471-1570 | GGAGTTGTACGGCAGTGGCTGAACCAAGGAGACGGAATACAGGGCTTTCGATTCAGCGCTCACTGCTCTTGTGACAGCAAAGATAACAAACTCCACGTGG | 82 | 78 | NM_011577.1:1470 |
| Tgfb1i1 | NM_001289550.1 | 1322-1421 | TCCGGAGGCAGCTTTTTTGAGCACGAGGGTCGCCCTTTGTGTGAAAACCATTTCCATGCTCAGCGTGGTTCGCTGTGTGCCACGTGTGGTCTCCCAGTGA | 83 | 83 | NM_001289550.1:1321 |
| Tgfb2 | NM_009367.1 | 1686-1785 | CCCAAAGCCAGAGTGGCCGAGCAGCGGATTGAACTGTATCAGATCCTTAAATCCAAAGACTTAACATCTCCCACCCAGCGCTACATCGATAGCAAGGTTG | 82 | 79 | NM_009367.1:1685 |
| Tgfb3 | NM_009368.2 | 2411-2510 | TCATGTAATTAGTTTCTGGGCCAGCAACTAGCTATCTCAGGTCCCTTAGAGATGCTGGACTCAAAAGCAGAGGTCAGAATTGGTTCTCTCATGTATTCCC | 80 | 82 | NM_009368.2:2410 |
| Thbs1 | NM_011580.3 | 4-103 | ACTTCTGCAGGCAATCGCGAAGCTGCTATCCAGTTCTGCCACGGTCTCTCCCGGCGCACCGGCAGTCTCAGCGTCTTCACCGGACTCAGCGTCCTTGTCC | 86 | 86 | NM_011580.3:3 |
| Tinf2 | NM_145705.3 | 709-808 | AAGAGCTTCAGGATGCTCTAAGTTGGAGTCAGCCGGGCTCGTTCATCACTTCTTCGGTTGCTTTGCACCAGTATGGTATGGACATGGGGTGGACATTTCC | 81 | 81 | NM_145705.3:708 |
| Tlr2 | NM_011905.2 | 256-355 | GCAGGCGGTCACTGGCAGGAGATGTGTCCGCAATCATAGTTTCTGATGGTGAAGGTTGGACGGCAGTCTCTGCGACCTAGAAGTGGAAAAGATGTCGTTC | 82 | 80 | NM_011905.2:255 |
| Tlr4 | NM_021297.2 | 2511-2610 | AACGGCAACTTGGACCTGAGGAGAACAAAACTCTGGGGCCTAAACCCAGTCTGTTTGCAATTAATAAATGCTACAGCTCACCTGGGGCTCTGCTATGGAC | 81 | 79 | NM_021297.2:2510 |
| Tmem135 | NM_028343.4 | 911-1010 | CCAAGGCACAGATGCTGCAAGCATTATGAAGACAACTGCATTTCCTACTGCATTAAAGGCTTCATCAGAATGTTCAGCGTGGGCTACTTGATCCAGTGCT | 82 | 80 | NM_028343.4:910 |
| Tmem33 | NM_028975.4 | 891-990 | TAATGAACTGAGGATTGTGGTTGAACACATTATTATGAAGCCCTCCTGCCCACTGTTTGTGAGAAGACTATGTCTCCAGAGCATCGCCTTTATCAGCAGA | 81 | 80 | NM_028975.4:890 |
| Tollip | NM_023764.3 | 261-360 | CTCAGCATCACTGTGGTACAGGCAAAATTGGCAAAGAATTATGGCATGACTCGTATGGACCCTTACTGCCGTCTGCGTCTGGGCTATGCTGTTTATGAAA | 81 | 82 | NM_023764.3:260 |
| Tpp1 | NM_009906.5 | 717-816 | TACTTCCATAACTCGGATCTGACTGAGTTCATGCGCCTATTCGGTGGCAGTTTTACACACCAGGCCTCAGTAGCAAAAGTTGTTGGAAAGCAAGGGCGAG | 82 | 82 | NM_009906.5:716 |
| Trp53 | NM_011640.1 | 1836-1935 | CCCTCTCTGAGTAGTGGTTCCTGGCCCAAGTTGGGGAATAGGTTGATAGTTGTCAGGTCTCTGCTGGCCCAGCGAAATTCTATCCAGCCAGTTGTTGGAC | 82 | 83 | NM_011640.1:1835 |
| Trp53bp1 | NM_013735.3 | 3795-3894 | TGGCCATGTCTTGCATCGCCACATGAGAACCATTCGAGAAGTCCGTACACTCGTCACCCGCGTCATCACAGATGTTTATTATGTGGATGGGACAGAAGTG | 81 | 80 | NM_013735.3:3794 |
| Tsc1 | NM_022887.3 | 4646-4745 | GCTGAGGGGTTGCCTCACCTTACTAGGTTCTCACAGCTTTTACACCAACTCCAACTTCAGGAAAGACCCTCCCTGGAGCTTCATATCAAACATTTCTGTT | 82 | 79 | NM_022887.3:4645 |
| Tsc2 | NM_001039363.1 | 1555-1654 | GTTCTATGAGGAGGAGCTGATTAACTCGGTGGTCATCTCGCAGCTCTCCCACATTCCCGAGGATAAGGACCATCAGGTCCGAAAGCTGGCTACTCAGCTG | 85 | 85 | NM_001039363.1:1554 |
| Twist1 | NM_011658.2 | 995-1094 | AATGGACAGTCTAGAGACTCTGGAGCTGGATAACTAAAAATAAATCTATATGACAAAGATTTTCATGGAAATTAGAAGAGCAGAGACCAAATTCACAAGA | 82 | 78 | NM_011658.2:994 |
| Txnip | NM_023719.1 | 2341-2440 | CCTGAGTGCTGCGATCAAAGGCCCAGCTTGGTTATTGCTTTTGAGGCTTTCTCCCAACGCACAGACTTGTGTAATTCTAACACTAATCCTGTGAAGGGTT | 80 | 81 | NM_023719.1:2340 |
| Vps13c | NM_177184.3 | 5025-5124 | AAGTCTTCCGGTTCCAGATGAGTCTGTATCCAGATGCCACAGAAGGAGAGAATTATGGCGATATGTCTAAAGTGGATGGCAGACTTAGTCTCAAAGTGGG | 83 | 81 | NM_177184.3:5024 |
| Vwa5a | NM_172767.3 | 2529-2628 | TGCTTGTGCAAGCTGCCAATAGTCTCCTGAAATTATCTGTGAATCCTGCTGTCTTTGGCGTCTAAACATGTCACCCATGAAAGGAAAGCCTTTTCTGGCG | 81 | 80 | NM_172767.3:2528 |
| Wrn | NM_001122822.1 | 1903-2002 | TAGGGTCATCTACATAACTCCAGAGTTCTGTTCTGGTAACTTGGATCTACTCCAGCAACTTGACTCTAGTATTGGCATCACTCTCATTGCTGTGGATGAG | 79 | 82 | NM_001122822.1:1902 |
| Zbtb10 | NM_177660.3 | 6739-6838 | TCCTCCCTGTGAGATGCTCTGTTAGGAGTCCTGTTCTCTTTGGAAGTTGCTTTTGTCACTTTTGACTCAAGCACACCTCACTGCCATCCTCTGTGAGTGG | 81 | 80 | NM_177660.3:6738 |
| Zfp9 | NM_011763.2 | 2981-3080 | ACCAGTGGCTCAAAGTGAGATTCTAGGCTCTTACTACCTTTGTTGACCCAGATCCCAGCATGGACATACCATGTAACTTCACTGTGCCTCACCTTCTTCC | 79 | 82 | NM_011763.2:2980 |
| Zfr | NM_011767.2 | 2595-2694 | CGTGTCTGAGGCAGCGATAATTTTGAACTCGTGTGTGGAGCCCAAAATGCAAGTCACTATCACCCTGACATCTCCAATTATTCGAGAAGAGAACATGAGG | 82 | 81 | NM_011767.2:2594 |
| Zmpste24 | NM_172700.2 | 1435-1534 | ACCTTACAATGAGGTTCTGTCTTTCTGCCTAACAGTCCTGAGCCGCAGATTTGAATTTCAAGCTGATGCGTTTGCCAAGAAACTTGGGAAGGCTAAAGAC | 82 | 81 | NM_172700.2:1434 |

**Supplementary Table 2: Codeset Information for the custom-designed Mitochondria Panel**

| **Customer Identifier** | **Accession** | **Position** | **Target Sequence** | **Tm CP** | **Tm RP** | **NSID** |
| --- | --- | --- | --- | --- | --- | --- |
| Abcf1 | NM_013854.1 | 876-975 | GAGGTGTCTTCCCGCCAGGCAATGTTAGAAAATGCATCTGACATTAAGTTGGAAAAGTTCAGCATCTCCGCCCACGGCAAGGAGCTATTCGTCAATGCTG | 79 | 83 | NM_013854.1:875 |
| Aifm2 | NM_153779.2 | 341-440 | GGGCAAAGTGATTGGCATAGACTTGAAGAACCGGATGGTGTTGCTACAGGGTGGCGAGGCCTTGCCCTTCTCACATCTTATCCTGGCCACAGGCAGCACC | 82 | 82 | NM_153779.2:340 |
| Aip | NM_016666.2 | 936-1035 | GTGGCTCAGGAGTATTATGAAGTGTTGGATCACTGCTCCTCCATCCTCAACAAGTATGATGACAATGTCAAGGCTTACTTCAAGAGGGGTAAGGCCCATG | 82 | 83 | NM_016666.2:935 |
| Atp12a | NM_138652.2 | 2835-2934 | GGGAGGTTTCCTCGTCTATTTCACTGTCTATGCACAGCAGGGCTTTTGGCCCACCTCTCTCATCAACCTGAGGGTATCATGGGAAACAGATGACATAAAT | 81 | 81 | NM_138652.2:2834 |
| Atp4a | NM_001290627.1 | 3361-3460 | AAATTAAGCGGTTGGCAGTTCCTGTGGAGGTGTGTCCATCTCAAGGACTCCATCTGGACAAGAAGGTTGTCTGTTACAAACCCCTCATGTTCTACTGGGC | 82 | 83 | NM_001290627.1:3360 |
| Atp4b | NM_009724.2 | 1131-1230 | GCCACTAAGGAAGGCCTATCTGTCCACATTTCTGTCCTGTGATCATTTGCCCGTCCTGCACTTCAATATGAACTATGGGTCCACATCAGGGTAACACTGG | 81 | 82 | NM_009724.2:1130 |
| Atp5a1 | NM_007505.2 | 1761-1860 | GGGGTTATCTTGACAAACTGGAGCCCAGCAAGATCACAAAGTTTGAGAATGCTTTCTTGTCTCACGTTATCAGCCAGCACCAGTCCCTCTTGGGCAATAT | 82 | 80 | NM_007505.2:1760 |
| Atp5b | NM_016774.3 | 1306-1405 | AGGACTACAAATCTCTCCAGGACATCATTGCCATCTTGGGTATGGATGAACTTTCTGAGGAAGATAAATTGACTGTGTCCCGGGCAAGAAAGATACAGCG | 83 | 83 | NM_016774.3:1305 |
| Atp5c1 | NM_020615.4 | 448-547 | TGAAGAATGAAGTGGCTGCCCTCACAGCAGCTGGGAAAGAAGTTATGATTGTTGGAGTTGGTGAAAAAATCAAGGGCATACTTTATAGGACTCATTCTGA | 85 | 77 | NM_020615.4:447 |
| Atp5d | NM_025313.2 | 77-176 | CTCCCTGAAGGCCGCCTCGCTTGCCCAGTGTGTCGGCCGCCCGCGAAGCTAGAGTCCACTGACTTTTCCGCCACCATGTTGCCCGCCTCACTGCTTCGTC | 85 | 84 | NM_025313.2:76 |
| Atp5e | NM_025983.3 | 187-286 | AAGACCGAGTTCAAAGCGAACGCTGAGAAGACTTCGGGCAGCAGCATAAAAATTGTGAAAGTCTCGAAGAAGGAGTAGCTGAATCTGAAGCCTGAAGTGC | 85 | 85 | NM_025983.3:186 |
| Atp5g1 | NM_007506.6 | 378-477 | CATCTCTCAAGCAGCAGCTCTTCTCCTATGCCATTCTGGGGTTTGCCCTGTCTGAGGCCATGGGACTCTTCTGTTTGATGGTCGCCTTCCTCATCCTCTT | 83 | 81 | NM_007506.6:377 |
| Atp5g2 | NM_026468.2 | 187-286 | GATGAGAGCCTCAGCAGCTTGGCGGTCCGGCGGCCTCTGACTTCACTTATCCCTAGCCGCAGTTTCCAAACCAGCGCCATTTCAAGGGACATCGACACAG | 82 | 82 | NM_026468.2:186 |
| Atp5g3 | NM_175015.2 | 173-272 | ATATAGACCAATTTCTGCATCAGTGTTATCTCGGCCAGAGACTAGGACTGGAGAGGGCTCTACAGTTTTTAATGGGGCCCAGAATGGTGTGTGTCAGCTG | 81 | 82 | NM_175015.2:172 |
| Atp5h | NM_027862.1 | 179-278 | AAACCACCTGCGATTGACTGGGCTTACTACAGGGCCAATGTGGCCAAGCCTGGCTTGGTGGATGATTTTGAAAAGAAGTATAATGCCCTGAAGATTCCTG | 93 | 79 | NM_027862.1:178 |
| Atp5j | NM_016755.2 | 356-455 | GAGGATCTTCAGGCTCTCCTCTGTCCTTCGGTCAGCAGTCTCTGTGCATTTGAAGAGGAACATTGGTGTTACAGCTGTGGCCTTTAATAAGGAACTTGAT | 82 | 81 | NM_016755.2:355 |
| Atp5j2 | NM_020582.2 | 53-152 | GTTCTGCGGACACCAGGACTTCAAGATGGCGTCACTCGTGCCGCTGAAGGAGAAGAAGCTCATGGAAGTCAAACTTGGAGAGCTGCCGAGCTGGATAATG | 83 | 83 | NM_020582.2:52 |
| Atp5k | NM_007507.3 | 227-326 | AGCTCAAGATGACAGCATTCTCAAGTGAGGCGTCAGCGAGCTTGCTTTTCTCTAGTCGTTGAGAACGAATAAAGCTTCATTGTGTGATGCTGTGAAAAAA | 82 | 76 | NM_007507.3:226 |
| Atp5l | NM_013795.5 | 249-348 | CATTCAAAGTGCTAAAACTGGTAGCTTCAAACACCTTACAGTTAAGGAAGCTGTGCTGAATGGTTTGGTGGCCACTGAGGTGTGGATGTGGTTTTATATC | 78 | 80 | NM_013795.5:248 |
| Atp5o | NM_138597.2 | 517-616 | CCTCTAGATGACGCTGTTCTCTCTGAGTTAAAGACGGTGCTGAAGAGCTTCCTGAGTCCAAACCAAATACTGAAACTGGAGATCAAGACTGACCCGTCAA | 83 | 84 | NM_138597.2:516 |
| Atp5f1 | NM_009725.3 | 705-804 | CTGAATATGGAGGAAAAGTGCGTCTTGGGCTGATTCCTGAGGAATTTTTCCAGTTCCTTTACCCTAAGACTGGTGTAACAGGACCTTATGTGCTTGGAAC | 79 | 82 | NM_009725.3:704 |
| Atp6v0a2 | NM_011596.4 | 201-300 | AAGGGCCTGGTGCAGTTCCGAGACCTCAATCAAAATGTAAGTTCTTTTCAAAGAAAATTCGTCGGTGAGGTAAAGAGGTGTGAAGAGCTCGAACGAATAC | 79 | 82 | NM_011596.4:200 |
| Atp6v0d2 | NM_175406.2 | 631-730 | TCTTACCTTGAGGCATTCTACAAATTCTGCAAGGATCACGGTGATGTCACAGCAGACGTTATGTGTCCCATTCTTGAGTTTGAGGCCGACAGACGCGCTT | 80 | 81 | NM_175406.2:630 |
| Atp6v1c2 | NM_001159632.1 | 741-840 | CCAAGTCAAGCTTTGCACAGTGGCAGAAGACATATGAGTCCCTATCGGACATGGTGGTCCCTCGGTCAACCAAATTGATCGCCGAGGACAACGAAGGTGG | 82 | 82 | NM_001159632.1:740 |
| Atp6v1e2 | NM_029121.3 | 757-856 | CATGCAGAAGATGCCAGAAATACGAGGGATTCTGTTTGGAGACAACACCAGCAGAAAGTTCTTTACATGAGGCTCTGGGACAGGGAGCCTGGGCTAAACT | 82 | 82 | NM_029121.3:756 |
| Atp6v1g3 | NM_177397.3 | 1109-1208 | AAATTATCTTAGGCCTTGTGTGGAGAAACAGACATCTAGTAGAGCTGGTCCCTTGCTTCCCTGTGAGGAATGTCCACTTTAACTCAGATGTGGAGAGCCC | 82 | 81 | NM_177397.3:1108 |
| Bak1 | NM_007523.2 | 471-570 | GTCGGCAGCTTGCTCTCATCGGAGATGATATTAACCGGCGCTACGACACAGAGTTCCAGAATTTACTAGAACAGCTTCAGCCCACAGCCGGGAATGCCTA | 81 | 82 | NM_007523.2:470 |
| Bbc3 | NM_133234.1 | 1462-1561 | CCCCAATCCCCATCCATCTCATTGCATAGGTTTAGAGAGAGCACGTGTGACCACTGGCATTCATTTGGGGGGTGGGAGATATTGGCGGAAGCCACCCCAG | 82 | 84 | NM_133234.1:1461 |
| Bcl2 | NM_009741.3 | 1845-1944 | GGCCTTCTTTGAGTTCGGTGGGGTCATGTGTGTGGAGAGCGTCAACAGGGAGATGTCACCCCTGGTGGACAACATCGCCCTGTGGATGACTGAGTACCTG | 85 | 87 | NM_009741.3:1844 |
| Bcl2l1 | NM_009743.4 | 201-300 | GAGCAACCGGGAGCTGGTGGTCGACTTTCTCTCCTACAAGCTTTCCCAGAAAGGATACAGCTGGAGTCAGTTTAGTGATGTCGAAGAGAATAGGACTGAG | 81 | 81 | NM_009743.4:200 |
| Bcs1l | NM_025784.4 | 159-258 | GTGCCTCAGAGCCTATAAGCATCTGTGCTGCTTCTTTTCAAGATGCCATTTTCAGACTTTGTTCTGGCCCTTAAAGACAATCCCTACTTTGGGGCTGGAT | 79 | 81 | NM_025784.4:158 |
| Bid | NM_007544.3 | 1308-1407 | AATCATGCACACGACTTATCTGTGTGGTGTCAGTTACACTCAGGCTCTTGCTACGGAATGCAAAGAACAACTCACATACCAGTGTCAAACAGAATGCACA | 80 | 82 | NM_007544.3:1307 |
| Bnip3 | NM_009760.4 | 1109-1208 | TGTGGCCTTATAATCCTATTACATAGCAGAAAATTAAAGGGTGCGTGCGGGTTATCTGTAAAGGCCTCTAACTTTGTGAACTGAGTAGCAAGTAGAAGCT | 80 | 80 | NM_009760.4:1108 |
| Cdkn2a | NM_001040654.1 | 567-666 | CCAATCCCAAGAGCAGAGCTAAATCCGGCCTCAGCCCGCCTTTTTCTTCTTAGCTTCACTTCTAGCGATGCTAGCGTGTCTAGCATGTGGCTTTAAAAAA | 83 | 78 | NM_001040654.1:566 |
| Cox10 | NM_178379.3 | 717-816 | TTTTTGAGGTGCCGTTCGACTCAAACATGAATAGAACAAAGAACAGGCCTCTGGTTCGAGGGCAGATAAGCCCATTGCTAGCCGTGTCTTTTGCCACCTG | 80 | 81 | NM_178379.3:716 |
| Cox11 | NM_199008.2 | 1177-1276 | AGGGATATGAATCAGTGGGTTAAATAAAGTGTTTGCCACATAGGCCTGACAACTTGATTTCTATTCCCGGAACCAGCCTTAAAAGAGTGACTACAGTAGC | 82 | 80 | NM_199008.2:1176 |
| Cox18 | NM_001033310.2 | 346-445 | TAAGGTGGAAAACTTGCAACCAGAAATAAAAGACATCGCCAAGCGCCTTAACCAAGAAGTTGCAGTCTGTGCCCGTCAGTTTGGATGGTCCAAGAGAGTG | 82 | 82 | NM_001033310.2:345 |
| Cox4i1 | NM_009941.2 | 71-170 | TTGGCTTCCAGAGCGCTGAGCCTGATTGGCAAGAGAGCCATTTCTACTTCGGTGTGCCTTCGAGCACATGGGAGTGTTGTGAAGAGTGAAGACTATGCTT | 82 | 83 | NM_009941.2:70 |
| Cox4i2 | NM_053091.2 | 381-480 | TCTTCTTCTTCATTGGATTCACGGCTCTGGTGATTTGGTGGCAGCGAGTCTATGTGTTCCCTAAGAAGGTTGTCACCCTGACGGAAGAACGGAAAGCCCA | 78 | 83 | NM_053091.2:380 |
| Cox5a | NM_007747.2 | 283-382 | GCCTGGGAATTGCGTAAAGGGATGAATACACTTGTTGGCTATGATCTGGTTCCTGAGCCCAAAATCATTGATGCTGCATTGCGAGCATGTAGACGGTTAA | 82 | 82 | NM_007747.2:282 |
| Cox5b | NM_009942.2 | 333-432 | ATCTAGTCCCGTCCATCAGCAACAAGAGAATAGTGGGCTGCATCTGTGAAGAGGACAACTGTACTGTCATCTGGTTTTGGCTGCACAAAGGCGAGAGTCA | 82 | 82 | NM_009942.2:332 |
| Cox6a1 | NM_007748.3 | 379-478 | CACAGCACTGGTTTTGGACCCTTACTCTGTGTGGACCACGAAAACCCTTTGGATGCTAAGCTCGTGTCTCCTTTCCTCAGATGGCGACCATTACTCTGAT | 79 | 81 | NM_007748.3:378 |
| Cox6a2 | NM_009943.2 | 468-567 | GGTGTGGAAGTTTTGTGTCCTCTGGCTCTTTGGGAACAGCATGGTGGAAGGGGCTGGGCAGGCTCTTGGGCAGTTGGTATCTGGGTTCCAGTTATTTTTT | 82 | 80 | NM_009943.2:467 |
| Cox6b1 | NM_025628.3 | 349-448 | CTCTCCTCTGTTCTTTGTCTTTCTCCCCGGATAGAAAAGGGGGACCTCAGCATATGATGGTCCTTACCCTGGGACCCTGAATCATGATGCAACTACTAAT | 81 | 84 | NM_025628.3:348 |
| Cox6b2 | NM_001289848.1 | 246-345 | ATCCGCGCTTCCCTAACCAGAACCAGACGCGTAACTGCTACCAGAATTTTCTGGACTACCACCGGTGTGTGAAGACCATGAATCGCCGCGGAAAGAGCAC | 83 | 85 | NM_001289848.1:245 |
| Cox6c | NM_053071.2 | 2-101 | CGCTTGAATACTTTTGGACTCCTTAGGCCACCGTCGCCGGGTCCTCCATCGACTCTTGCGCATGCGTGCTGCTGGAAGGCTCTCCGTTTCTCCGAGAACC | 83 | 85 | NM_053071.2:1 |
| Cox7a2 | NM_009945.3 | 420-519 | TGGCTGCATTTCCCAAGAAGCAGAACTAATGTCGTCATCCCAGTCTTCACGTGGTTCAGTTTCATCAACGCTCGATGGACCAGGAATCTGATGAGTAACT | 82 | 81 | NM_009945.3:419 |
| Cox7a2l | NM_001159529.1 | 1040-1139 | AGTGCTGCACATTAGACAGCTGTGTCAGGAGTTCTCCTTTAATAATGCTGTGGCTTTACGTTATGATTGACCGGACTGCGGAATAAACACTGGAATGAAG | 80 | 82 | NM_001159529.1:1039 |
| Cox7b | NM_025379.2 | 691-790 | AGCTGTTTTAGGAGCCCACTGGGTCATTGGCCATATAGGTTATGCTTACTGCCCTCTACCTCGTGGTTATATTTGGAATTGCCATTAGCTCCCTTCTGCT | 81 | 78 | NM_025379.2:690 |
| Cox8a | NM_007750.2 | 317-416 | GGAGCAGTCTTCCCTCATCCTTTGACTAGACCACTTTTGCCAGCCCACCTTGATCATGTTGCCTGCATTCCTGGCTGGCCTTCCCCGGGATCATGTTATT | 84 | 82 | NM_007750.2:316 |
| Cox8c | NM_001039049.1 | 215-314 | TTACGACCTTTTACATCCCAGCTGCATATGTGCTAAGCAGTCTGAAGTATTTCAAAGGAGAGTAGATACAGGATGTCCAGGAGCAGCACGGCATGGCTCC | 79 | 82 | NM_001039049.1:214 |
| Cpt1b | NM_009948.2 | 1298-1397 | ACAAGATGTCTCTGGACGCCATCGAACGTGCTGCTTTCTTTGTGACCCTGGATGAAGATTCTCATTGCTACAACCCTGACGATGAGACCAGTCTTAGCCT | 82 | 83 | NM_009948.2:1297 |
| Cpt2 | NM_009949.2 | 2026-2125 | GAGTTTCTCCACTGTGTCCAGAAGTGCTTGGAAGACATGTTCGATGCCCTCGAAGGCAAAGCCATCAAAACTTAGCTTCTTGGTCGATGAAAAGCCTCCA | 82 | 82 | NM_009949.2:2025 |
| Cyc1 | NM_025567.2 | 961-1060 | AGCGGCATAAGTGGTCAGTCCTGAAGAGTCGAAAGCTGGCTTATCGGCCACCCAAGTGACCCTGTTCAGTATCTGCTTGTCATCTTGCCAGAACAAGCTC | 83 | 82 | NM_025567.2:960 |
| Cycs | NM_007808.4 | 2511-2610 | CCTGTGAGATGCAGAAAGGGGACAGTCTCTGTACACTGATTTTACAAGTCAGGAAGTGGGACCACTCTAGCTGATCTGCTTGCTCTTAGTTGCTTTGTAG | 82 | 83 | NM_007808.4:2510 |
| Dnajc19 | NM_026332.3 | 212-311 | CCATGAAGCATGTGGAGCCTCAAGTAAAACAAGTTTTTCAGAGCCTACCAAAATCTGCATTCGGTGGTGGGTACTACAGAGGTGGATTTGAACCCAAAAT | 81 | 83 | NM_026332.3:211 |
| Dnm1l | NM_001025947.1 | 2076-2175 | GGTTAATCATGTGAAAGATACTCTTCAGAGTGAACTGGTAGGGCAGCTGTATAAGTCATCCTTATTAGATGACCTTCTGACTGAATCCGAGGACATGGCC | 82 | 82 | NM_001025947.1:2075 |
| Fis1 | NM_025562.3 | 303-402 | AGCAAAGAGGAACAGCGGGACTATGTCTTCTACCTGGCCGTGGGCAACTACCGGCTCAAGGAATATGAAAAGGCTCTAAAGTATGTGCGAGGGCTGTTGC | 83 | 82 | NM_025562.3:302 |
| Grpel1 | NM_024478.2 | 1639-1738 | CACTTGGGAGGTTGTGGTCAAGGGGATTAGAAGGCTCGTCTTTTATCTTCCCACTTTCTGGTACTGGATTTTGTCATCTAATGGAGATCTGGTAGGCTCC | 79 | 79 | NM_024478.2:1638 |
| Gusb | NM_010368.1 | 284-383 | CCCTTCGGGACTTTATTGGCTGGGTGTGGTATGAACGGGAAGCAATCCTGCCACGGCGATGGACCCAAGATACCGACATGAGAGTGGTGTTGAGGATCAA | 85 | 86 | NM_010368.1:283 |
| Hprt | NM_013556.2 | 31-130 | TGCTGAGGCGGCGAGGGAGAGCGTTGGGCTTACCTCACTGCTTTCCGGAGCGGTAGCACCTCCTCCGCCGGCTTCCTCCTCAGACCGCTTTTTGCCGCGA | 82 | 82 | NM_013556.2:30 |
| Hsp90aa1 | NM_010480.5 | 236-335 | CCTTGATCATCAATACCTTCTACTCGAACAAAGAGATCTTTCTGAGGGAGCTCATCTCCAATTCATCGGACGCTCTGGATAAAATCCGTTACGAGAGCCT | 78 | 80 | NM_010480.5:235 |
| Hspd1 | NM_010477.4 | 1067-1166 | GATTTGGGGACAATAGGAAGAACCAGCTTAAAGATATGGCTATTGCTACTGGTGGTGCAGTGTTTGGAGAAGAGGGGTTGAATCTAAATCTTGAAGATGT | 83 | 82 | NM_010477.4:1066 |
| Idh2 | NM_173011.2 | 374-473 | TGCTCTGGCCACCCAGAAGTACAGTGTGGCTGTCAAGTGTGCCACAATCACCCCTGATGAGGCCCGTGTGGAAGAGTTCAAGCTGAAGAAAATGTGGAAG | 85 | 85 | NM_173011.2:373 |
| Immp1l | NM_028260.2 | 185-284 | TAGAACAGACTTATGGCTGGTGGTAGCAGAGTCAACATCAAAGTATGCTTCGTGGTGTTCTGGGAAAGGCTTTTCGACTTGCTGGCTATACCATTCAGTA | 82 | 79 | NM_028260.2:184 |
| Immp2l | NM_053122.4 | 521-620 | TAGTAATTCTTTTGGACCGGTTTCTCTGGGACTCCTGCATGCCCATGCCACACATATTTTGTGGCCTCCAGAGCGCTGGCAGAGACTGGAATCTGTTCTT | 81 | 82 | NM_053122.4:520 |
| Immt | NM_029673.2 | 2006-2105 | GGGGGTATACAGTGAAGAGACCCTAAGAGCCCGTTTCTATGCTGTTCAAAAACTAGCCCGAAGGGTAGCGATGATTGACGAAACCAGAAATAGCCTGTAC | 79 | 81 | NM_029673.2:2005 |
| Ldha | NM_010699.1 | 256-355 | CAGAACAAGATTACAGTTGTTGGGGTTGGTGCTGTTGGCATGGCTTGTGCCATCAGTATCTTAATGAAGGACTTGGCGGATGAGCTTGCCCTTGTTGACG | 80 | 78 | NM_010699.1:255 |
| Lhpp | NM_029609.1 | 566-665 | ACGTTGGAGGTTACATGAAGGCGCTCGAGTATGCCTGTGGTATCAAGGCTGAAGTGGTGGGGAAACCCTCCCCTGAGTTCTTCAAGTCTGCTCTACAAGC | 82 | 82 | NM_029609.1:565 |
| Lrpprc | NM_028233.2 | 2451-2550 | AGAAGATGCGTGGAACTTGAAACAAGAAGTTGACCGCTTAGATGCTTCGGCTATTCTTGACACTGCCAAGTACGTAGCCCTTGTAAAAGTACTGGGAAAG | 83 | 81 | NM_028233.2:2450 |
| Mfn1 | NM_024200.4 | 2066-2165 | CTCTGTCACCTTAAGTATGTACGGAGCTCTGTACCTTTATGAGAGGCTGACGTGGACGACCCGTGCGAAAGAGAGAGCGTTTAAGCAGCAGTTTGTAAAC | 81 | 82 | NM_024200.4:2065 |
| Mfn2 | XM_006535920.1 | 693-792 | CTTTTTTTGGCCGGACGAGCAATGGGAAGAGCACCGTGATCAATGCCATGCTCTGGGACAAAGTTCTGCCATCTGGGATTGGTCATACCACCAATTGCTT | 82 | 82 | XM_006535920.1:692 |
| Minos1 | NM_001163006.2 | 2151-2250 | TTTAGATGGATGACTTTTGTATTCTGAGTGGCTGGGGAGCCTTCATTGGAGCACATTTACTGTCTACAGAGGCAGGCCTTTGTTCTGAGACGTGGCTTTT | 79 | 79 | NM_001163006.2:2150 |
| Mipep | NM_027436.3 | 571-670 | TGAATACAAACGTGGAGCTGTATCAGAGTCTGCAAAGGTTACTGGGTGATAAGAAGCTTATGGAGTCCCTTGATGCGGAAACCAGGCGAGTGGCCGAACT | 82 | 82 | NM_027436.3:570 |
| Mpv17 | NM_008622.1 | 731-830 | GTTCCAAGCACCCACATCGGGTGGCTCACAACTCCCTATGTCTCAAGCTCCAGGAGATCTGATGTTCTCTTCTGGCCTCTGAAGCACCTGCACAAACATG | 82 | 82 | NM_008622.1:730 |
| Msto1 | NM_144898.2 | 241-340 | CACTGCATGGCCAGGAAACCTATACGCCTCGACTCATCCTAATGGATCTGAAAGGTAGTCTGAACACTCTAAAAGAAGAAGGTAACCTCTACAGAGACCG | 82 | 82 | NM_144898.2:240 |
| Mtx2 | NM_016804.4 | 305-404 | ATGCAGCATCTCTTGCTGTGCAGGCATTTTTGCAGATGTGTAATCTGCCTGTCAAAGTGGTGTGTAGGGCAAATGCGGAATATATGTCTCCATCTGGAAA | 80 | 82 | NM_016804.4:304 |
| Ndufa1 | NM_019443.2 | 233-332 | TTCACCAACGGGGGCAAGGAAAAACGAGTTGCTCGTGTTCAGTACCAGTGGTATCTGATGGAACGCGATAGACGTATCTCTGGAGTCAATCGCTACTATG | 82 | 81 | NM_019443.2:232 |
| Ndufa10 | NM_024197.1 | 815-914 | ACCCAACCAAGGTGGTAGAGGACATTGAATACCTTAAGTACAACAAAGGGCCTTGGCTGAAACAGGACGACTGGACCTTTCACTACCTGCGGATGCTGGT | 82 | 82 | NM_024197.1:814 |
| Ndufa11 | NM_027244.1 | 2339-2438 | CCCACCTCTTGCCTCCATCTACCTCAGGGCCAAGGCTCAAGTCCTCTCTGCTCCAGCGGCTGCACCATGTTGTCACGTGTCATCTCGTCCTGTGTGTCCC | 83 | 82 | NM_027244.1:2338 |
| Ndufa2 | NM_010885.4 | 394-493 | ATGTGTGCTTTGGGTCCTTATAAAGCTTACGCTGTACAGTGTCCCTTCAGAATGTCCTCTTCATTACCTTCTCCCTCTTACTGCGCAACACTGAGGCAAA | 78 | 81 | NM_010885.4:393 |
| Ndufa3 | NM_025348.2 | 16-115 | CAAGAACAAGATGGCCGGGAGAATCTCTGCCTTCCTCAAGAATGCCTGGGCGAAGGAGCCGGTGCTGGTGGTGTCCTTCTCTGTCTGGGGCCTCGCTATA | 83 | 83 | NM_025348.2:15 |
| Ndufa4 | NM_010886.2 | 115-214 | CATCCCAGCTTGATTCCTCTCTTCGTATTTATTGGAGCAGGGGGTACTGGAGCAGCACTGTATGTGATGCGCTTGGCACTGTTTAATCCAGATGTCAGCT | 79 | 82 | NM_010886.2:114 |
| Ndufa5 | NM_026614.3 | 365-464 | GTGGTGAAGTGGAAGAGGTGATTCTTCAGGCTGAAAAAGAACTAAGTCTGGCAAGGAAAATGTTGAAGTGGAAGCCATGGGAGCCATTGGTGGAAGAGCC | 84 | 91 | NM_026614.3:364 |
| Ndufa6 | NM_025987.3 | 110-209 | CCAGTACCTCGGTGAAGCCCATTTTCAGTCGCGACCTGAACGAGGCCAAGCGGAGGGTGCGCGAGCTCTACCGCGCTTGGTATCGGGAGGTGCCGAACAC | 86 | 85 | NM_025987.3:109 |
| Ndufa7 | NM_023202.2 | 156-255 | ACAAGCTGTCCAACAATTACTACTGTACTCGTGATGGCCGCCGGGAAGTTGTGCCTCCCTCAATCATCATGTCCTCACAAAAGGCCCTGGTGTCAGGCAA | 81 | 81 | NM_023202.2:155 |
| Ndufa8 | NM_026703.2 | 45-144 | GCCGTCATGCCGGGGATAGTGGAGCTGCCAACTCTGGAAGAGCTGAAAGTGGAGGAGGTGAAAGTCAGCTCAGCTGTGCTTAAAGCTGCCGCCCATCACT | 86 | 85 | NM_026703.2:44 |
| Ndufab1 | NM_001360743.1 | 255-354 | GACGGAATCAAGGACCGAGTTCTGTATGTCTTGAAACTCTATGATAAGATTGATCCAGAAAAGCTCTCCGTAAATTCTCATTTTATGAAGGACCTGGGCT | 79 | 80 | NM_001360743.1:254 |
| Ndufb10 | NM_026684.2 | 179-278 | ACTGGCCCGTGACCCTCGTGAGAGAGTTTATTGAACGACAGCATGCCAAGAACCGAACCTACTACTACCACCGACAGTACCGTCGAGTGCCAGACATCAC | 82 | 83 | NM_026684.2:178 |
| Ndufb2 | NM_026612.3 | 233-332 | CTGGATTCTTTGGCGATTTTGGCATGACTCGGATGCTGTGCTGGGTCACTTTTCATATCCAGATCCTTCACAGTGGACAGATGAAGAACTGGGAATCCCT | 82 | 83 | NM_026612.3:232 |
| Ndufb3 | NM_025597.2 | 343-442 | CTGAATATTTCCTGGATTCCCAGAATGGTGATAAGAAGCATCACTGAAGAGAGCGCCTTGTGACGTCTCTTCCATAAAAATAAGATTCTCTCACTGTAGC | 80 | 78 | NM_025597.2:342 |
| Ndufb4 | NM_026610.1 | 198-297 | TGCCTTGATTCGCTGGACCTATGCAAGATCAGCAAATATTTATCCTAATTTCAGGCCCACTCCCAAGAACTCACTTTTAGGAGCTGTGGCAGGGTTTGGG | 77 | 74 | NM_026610.1:197 |
| Ndufb5 | NM_025316.2 | 107-206 | GGAAGTTTTCTCACGCGGAGCTTTCCGAAGACTGTCGCTCCTGTGCGGCACAGTGGGGACCATGGCAAGAGACTGTTTGTCGTCAAGCCTTCTTTATACT | 82 | 81 | NM_025316.2:106 |
| Ndufb6 | NM_001033305.2 | 311-410 | CCATACACCATTGTTAGCTCGAAGCCCAGGATATTTCCAGGTGATACAATTCTGGAGACTGGAGAAGTAATTCCACCAATGAGAGATTTTCCTGATCAAC | 79 | 82 | NM_001033305.2:310 |
| Ndufb7 | NM_025843.3 | 213-312 | TGACACTGCAGCAACGTGACTACTGTGCCCACTACCTCATCCGGCTGCTGAAGTGCAAGCGAGACAGCTTCCCCAACTTCCTGGCCTGCAAGCACGAGCA | 85 | 85 | NM_025843.3:212 |
| Ndufb8 | NM_026061.2 | 380-479 | CCCTGTGTCCTGGGATGTCATGTGTAAACATCTCTTCGGCTTTGTGGCTTTCATGGTTTTCATGTTCTGGGTAGGGCACGTGTTCCCTTCCTACCAGCCT | 80 | 80 | NM_026061.2:379 |
| Ndufb9 | NM_023172.3 | 295-394 | TTCCTTCGAGAGATATGAGTGCTACAAGGTTCCAGAATGGTGCTTAGATTACTGGCATCCCTCTGAGAAAGCAATGTATCCTGATTACTTTTCCAAGAGA | 79 | 80 | NM_023172.3:294 |
| Ndufc1 | NM_025523.1 | 273-372 | AGTCAGCTTCATGTGATGATCACAGTCCTTCCTCTGGATTCACCCTCAGTGACGGATGTGAGAAAAGAGTTACATGCGAATATGAGTCAGTCAACACGTT | 81 | 80 | NM_025523.1:272 |
| Ndufc2 | NM_024220.2 | 270-369 | CCCGCGGCTTGTCTACATGGGCTTGTTGGGCTACTGCACGGGCCTGATGGACAACATGCTGCGGATGCGACCGGTGATGAGAGCAGGTTTGCACCGCCAG | 88 | 87 | NM_024220.2:269 |
| Ndufs1 | NM_001160038.1 | 553-652 | CCTAAAGGATATGTTCGCACAACTGGCACAGCAGCAAGTAACTTGATTGAAGTATTTGTTGATGGTCAGTCTGTCATGGTGGAACCAGGAACCACTGTTC | 83 | 80 | NM_001160038.1:552 |
| Ndufs2 | NM_153064.4 | 403-502 | AAGACCTATCTGCAGGCCCTTCCATACTTTGACCGGTTGGACTATGTGTCCATGATGTGTAATGAACAGGCCTATTCGATAGCTGTGGAGAAGTTGCTAA | 82 | 81 | NM_153064.4:402 |
| Ndufs3 | NM_026688.2 | 351-450 | ATCCCAACGCTGACTTTTCTCAGGGATCACACCAATGCACAATTCAAATCCTTGGCTGACTTGACGGCAGTGGATGTCCCAACTCGGCAGAACCGTTTTG | 80 | 82 | NM_026688.2:350 |
| Ndufs4 | NM_010887.1 | 71-170 | GTAGAGTTCCATCCAGGTTGTTGAGCACATCCACTTGGAAGCTGGCAGACAACCAGACTCGGGACACACAGCTTATAACAGTTGATGAGAAACTGGATAT | 82 | 81 | NM_010887.1:70 |
| Ndufs5 | NM_001030274.1 | 111-210 | TCAGCCTGGACCGGCACTTTATGTTCCTAAGCGCAGAACAGCCCTATAAGAACGCCGCTCGGTGCCACGCATTTGAAAAAGAGTGGATAGAGTGTGCACA | 82 | 80 | NM_001030274.1:110 |
| Ndufs6 | NM_010888.2 | 410-509 | TGACTCCTATGGAACATCTCCACGCTGGGTGTTCTGTGTGAGGCCACTGCTCTGTGAATGGTGTCCCTTGTTTTGAATAAAGGATGCTCCCACCATGAAA | 88 | 79 | NM_010888.2:409 |
| Ndufs7 | NM_029272.4 | 453-552 | CCCCGCGCTCCGAAAGGTGTACGACCAGATGCCCGAACCCCGCTATGTGGTGTCCATGGGGAGCTGTGCCAATGGCGGTGGCTACTACCACTACTCCTAC | 92 | 92 | NM_029272.4:452 |
| Ndufs8 | NM_144870.5 | 721-820 | GCAGCCGCCGAACGACACGCTATGACATCGACATGACCAAGTGTATCTACTGTGGTTTCTGCCAGGAAGCCTGCCCTGTTGATGCCATTGTGGAGGGCCC | 82 | 82 | NM_144870.5:720 |
| Ndufv1 | NM_133666.2 | 1151-1250 | GGCACGGCTGCAGTTATTGTTATGGATCGCTCGACAGACATTGTGAAAGCCATCGCTCGTCTCATTGAGTTCTACAAGCATGAGAGCTGTGGCCAGTGTA | 83 | 82 | NM_133666.2:1150 |
| Ndufv2 | NM_001278415.1 | 545-644 | TACAATGTATAATCGAAAGCCAGTTGGGAAGTACCATATCCAGGTCTGCACTACTACACCTTGCATGCTGCGAGATTCTGACAGCATATTGGAGACCCTT | 81 | 82 | NM_001278415.1:544 |
| Ndufv3 | NM_001083891.1 | 103-202 | GCGGTCTCTCTCCTCCTGCGGGGAGGACGGATCCGGGCGCTGAAGGCTGTGCTCCTGGAGGCAAGGGTGTTCCCGGGAGAACTGGTTTCTGTAGTGAGGC | 93 | 91 | NM_001083891.1:102 |
| Nefl | NM_010910.1 | 1871-1970 | CCTCCTTACGCAGAGTATCTGTTTGCTTGCAGAGTGGCTTTCTGGCTTGCTGCCAGCCTGTGCATGGTCCATGCTTATGAGTTCAGGATCTATGGCAATG | 80 | 83 | NM_010910.1:1870 |
| Nrf1 | NM_010938.3 | 571-670 | CCTCATGTGTTTGAGTCTAATCCATCTATCCGAAAGAGACAGCAGACACGTTTGCTTCGGAAACTCAGAGCCACGTTGGATGAGTACACGACGCGAGTGG | 80 | 82 | NM_010938.3:570 |
| Opa1 | NM_001199177.1 | 2846-2945 | CTACCAGAGGCATTTTATAGATTCTGAGCTGGAATGCAATGACGTGGTCCTGTTTTGGCGAATACAGCGCATGCTCGCTATCACTGCCAATACATTAAGG | 81 | 80 | NM_001199177.1:2845 |
| Oxa1l | NM_026936.3 | 1426-1525 | TTTCCACTACAGACTCTTATTTGTGTGTATTAAAAGAGCCCTGGAGAGCCAAGCAGCCTTCCATCCACAGAACTAGTAAACCCCTGTCTTGTCTGGGCTT | 79 | 82 | NM_026936.3:1425 |
| Pink1 | NM_026880.2 | 689-788 | GGAGCAGGCTCCAGGGACTCCCACCTTTCCCTTTGCCATCAAGATGATGTGGAATATCTCGGCAGGTTCCTCCAGCGAAGCCATCTTAAGCAAAATGAGC | 82 | 82 | NM_026880.2:688 |
| Pmaip1 | NM_021451.2 | 1431-1530 | TGTGACACTCAGACTGCTTAATAGCAAATCAGTCCTGGTTAACAGAGAGAGGCAAGAAACTTGAGTAGACATTCCCAAAAGGCAGGTGGCCAGCAGATAC | 78 | 82 | NM_021451.2:1430 |
| Polr1b | NM_009086.2 | 2796-2895 | TGCCTTTCACTGAGAGTGGCATGATGCCGGACATTCTGTTTAATCCTCACGGGTTTCCCTCCCGTATGACCATAGGTATGTTAATCGAGAGCATGGCTGG | 81 | 83 | NM_009086.2:2795 |
| Ppa1 | NM_026438.4 | 1106-1205 | CCACTAGTTCAGAACTGGTTCAGAAGGTTAGCCATCTAGACACCTCACATCTCAACCAAGACAGCTTTTATTAAACCTTAGCTCTCAAAGCCGTGGGGAC | 83 | 82 | NM_026438.4:1105 |
| Ppa2 | NM_146141.1 | 465-564 | TCACCTAAGAGATAAGAGCACCGACTGCTGTGGGGATAATGATCCCATTGACGTCTGTGAAATAGGCTCAAAGGTTCTTTCCCGTGGAGACGTGGTCCAT | 83 | 82 | NM_146141.1:464 |
| Pprc1 | NM_001081214.1 | 1897-1996 | AGTCCTGGCTGACAAGAAAGGAATTGAACCTGCAGTGGCTATTCCCACTTCGGATAACTTGTCTCCAGCTGATGTCCTAGCAAACACAGTGGCAGCTGAC | 82 | 82 | NM_001081214.1:1896 |
| Prkn | NM_016694.3 | 649-748 | GATGTCTTAATTCCAAACCGGATGAGTGGTGAGTGCCAGTCTCCAGACTGCCCTGGAACCAGAGCTGAATTTTTCTTTAAATGTGGAGCACACCCAACCT | 82 | 82 | NM_016694.3:648 |
| Rhot1 | NM_021536.7 | 941-1040 | TTAAGTGACGGTGTGGCTGACAGTGGGCTGACGCTCAGAGGTTTTCTCTTTTTACATACACTTTTTATCCAGAGGGGGAGGCATGAGACTACTTGGACTG | 82 | 81 | NM_021536.7:940 |
| Rhot2 | NM_145999.2 | 1855-1954 | GGACACCTCTCTAGACACTACCTGTGATGTCGCCTGCTTAATGTTTGACAGCAGTGATCCCAAGACCTTTGTACACTGTGCTACCATATACAAGCGTTAT | 82 | 81 | NM_145999.2:1854 |
| Rplp0 | NM_007475.5 | 496-595 | TCAGAACACTGGTCTAGGACCCGAGAAGACCTCCTTCTTCCAGGCTTTGGGCATCACCACGAAAATCTCCAGAGGCACCATTGAAATTCTGAGTGATGTG | 84 | 83 | NM_007475.5:495 |
| Sdha | NM_023281.1 | 251-350 | CTTGCGAGCTGCATTTGGCCTTTCTGAGGCAGGGTTTAATACTGCATGCCTTACAAAGCTCTTTCCTACCCGATCACATACTGTTGCAGCACAGGGAGGT | 82 | 82 | NM_023281.1:250 |
| Sdhb | NM_023374.3 | 566-665 | TTTGAGTAACTTCTACGCACAATACAAATCCATTGAGCCTTATCTGAAGAAGAAGGATGAGTCCCAGGAGGGCAAGCAACAGTATCTGCAGTCCATCGAG | 76 | 88 | NM_023374.3:565 |
| Sdhc | NM_025321.3 | 326-425 | ATGTTTGTGAAGTCCCTGTGTTTGGGGCCAACACTGATCTACTCGGCTAAGTTTGTGCTTGTCTTCCCGCTCATGTACCACTCACTGAATGGGATCCGAC | 82 | 82 | NM_025321.3:325 |
| Sdhd | NM_025848.2 | 186-285 | CAAGCCACCACTCTGGTTCCAAGGCTGCATCTCTCCACTGGACCAGTGAGAGGGTTGTCAGTGTTCTGCTCTTGGGGCTGATCCCTGCTGGGTACTTGAA | 82 | 81 | NM_025848.2:185 |
| Sfn | NM_018754.2 | 43-142 | AATCTGATTTGGTAATCCAAGACGCTCCTGCAATGCAGCCAGCCCTGAACTGCAGGGGGCAGTCTGGAGCCGAAAGGTGCCTTTGCAGGTGGGACCTGCG | 87 | 88 | NM_018754.2:42 |
| Sh3glb1 | NM_019464.2 | 1061-1160 | ATCAGACCTCTGGGACACCAGTGCCATATGCTTTGTCAAATGCAATTGGTCCTTCTGCCCAGGCTTCAACGGGTAGCCTTGTAATCACCTGTCCTTCTAA | 83 | 82 | NM_019464.2:1060 |
| Slc25a1 | NM_153150.2 | 1183-1282 | TCAAGTTCTGTGGCCTGGTGTGTCCCATAGTTCTGTATATCCCATGTGGTCTGTGTGTGTGTAACACTACCATTGTATCTGTGTCCAGCCTGGCCATGGC | 80 | 82 | NM_153150.2:1182 |
| Slc25a10 | NM_013770.1 | 1389-1488 | GGGTACGGCCCGTGGCAGCTTCTGCTTACCAAATGACTAGAGCACACACACAAGCACTTTGTCACAAGAGGGACCACCGTGCTGTGTTCTGGAAGGTAGT | 83 | 82 | NM_013770.1:1388 |
| Slc25a12 | NM_172436.3 | 1459-1558 | CTTTTCGGGCTGTACAAGGGTGCCAAAGCCTGTTTCCTCCGAGACATTCCCTTCTCCGCAATCTATTTTCCCGTGTACGCTCACTGCAAACTCCTCCTGG | 82 | 79 | NM_172436.3:1458 |
| Slc25a13 | NM_001177572.1 | 930-1029 | TGGCAGCCCAGAAGTTTGGTCAGGTTACACCCATGGAAGTTGACATCTTGTTTCAGTTAGCAGATTTATATGAGCCGAGGGGGCGCATGACCTTAGCAGA | 83 | 82 | NM_001177572.1:929 |
| Slc25a14 | NM_001166450.1 | 255-354 | AGTTCCACTTTAAGCCATGAGATGTCTGGTCTGAACTGGAAACCTTTTGTGTATGGCGGCCTTGCCTCTATTGTTGCCGAGTTCGGCACTTTCCCTGTGG | 78 | 82 | NM_001166450.1:254 |
| Slc25a15 | NM_181325.4 | 2721-2820 | TCTTTCCCTTCTAGGTGCCTGGGTCTAGTGCACATTAGTCTTGTTGGCAGCGTGTCTCCTCAGTCTGGCTATTGTGATCTTTCCCATAGAAAGAGTCAGG | 80 | 79 | NM_181325.4:2720 |
| Slc25a16 | NM_175194.2 | 991-1090 | ATGGACAGCATGGGATTCGGAGAGGATTGTACCGTGGCTTATCTCTGAACTACATCCGCTGTATCCCCTCTCAAGCTGTGGCTTTCACAACGTATGAGCT | 81 | 81 | NM_175194.2:990 |
| Slc25a17 | NM_011399.3 | 707-806 | GACGGTACAGTCAATTCTGAGGTTTGGACGTCATAGACTGAACCCAGAAAACAGGACCCTGGGAAGTCTTCGGAATGTTCTCTCTCTTCTTCACCAGCGA | 83 | 81 | NM_011399.3:706 |
| Slc25a19 | NM_001252384.1 | 2505-2604 | GTCACTTGGGAGGGCTTCTGAGATGGGTGGCCCGTTGGCTTCCTGGGCTTGGAGCAGCAGAAGTGTTTTGTGACCCTATTGCTGGAGAGATGTGAATAAA | 83 | 83 | NM_001252384.1:2504 |
| Slc25a2 | NM_001159275.1 | 757-856 | GAACTAGGCCCTGTCCCTTTGATGTTAAGTGGAGGCTTTGCTGGGATCTGTCTCTGGCTTATCATATTCCCAGTGGACTGCATTAAATCCAGAATCCAGG | 82 | 80 | NM_001159275.1:756 |
| Slc25a20 | NM_020520.4 | 593-692 | TGCGCAAAGAAGCTGTATCAGGAGTTCGGGATCCGCGGCTTCTACAAAGGGACTGTGCTCACACTCATGCGAGATGTTCCTGCCAGTGGGATGTATTTCA | 83 | 83 | NM_020520.4:592 |
| Slc25a21 | NM_001167976.1 | 207-306 | GTTCCAGGTGCAGAGAAGTGTAACAGACCCACAAAGTTATAGAACTGTAAGAGGCAGCTTCCAGATGATCTTCCGAACAGAGGGGTTGTTTGGCTTTTAC | 83 | 81 | NM_001167976.1:206 |
| Slc25a22 | NM_026646.1 | 966-1065 | CAGTCCCTTGAGAGAGGTGTTAATGAGGACACTTACTCTGGGTTTCTGGACTGTGCAAGGAAGATCTGGAGACATGAAGGTCCCTCAGCCTTCCTGAAAG | 81 | 82 | NM_026646.1:965 |
| Slc25a23 | NM_025877.3 | 431-530 | CTTCTGCTCATGTTTCACAGCCTTGACCGGAACCAAGATGGTCACATAGATGTCTCTGAGATTCAACAGAGCTTCCGAGCACTAGGTATCTCCATCTCAC | 79 | 82 | NM_025877.3:430 |
| Slc25a24 | NM_172685.3 | 1101-1200 | TTACTAGGCATCATTCCTTACGCAGGCATTGATCTCGCTGTGTACGAGCTTTTGAAGTCTTATTGGCTGGATAACTTTGCCAAAGACTCGGTCAACCCTG | 81 | 79 | NM_172685.3:1100 |
| Slc25a25 | NM_001164358.1 | 3451-3550 | TCCAAACAGGATGCAAAGATCAATGCAAAAATCACCGGTCTGCATAGTCTATGCTGTAACTGGAGTTTGTCAAAGGCAAGCAGCCTTCTAATAAAGTCGC | 82 | 82 | NM_001164358.1:3450 |
| Slc25a27 | NM_028711.3 | 370-469 | TGAAGATAAGCATTATCCCCTCTGGAAATCGGTCATTGGAGGGATGATGGCTGGTGTCATCGGACAGTTTTTAGCCAACCCCACTGACCTTGTGAAGGTC | 84 | 82 | NM_028711.3:369 |
| Slc25a3 | NM_133668.3 | 458-557 | TATGAAGTCTTCAAAGCCTTATATAGCAACATACTTGGTGAGGAAAACACCTACCTGTGGCGCACATCACTGTATTTAGCTTCTTCTGCCAGTGCTGAAT | 79 | 81 | NM_133668.3:457 |
| Slc25a30 | NM_026232.3 | 231-330 | CACAGCAGAATGCGGCACATTTCCAATTGATTTAACTAAGACACGGCTTCAGATTCAAGGCCAGACAAATGATGCCAACTTCCGAGAGATCAGGTACCGA | 79 | 82 | NM_026232.3:230 |
| Slc25a31 | NM_178386.3 | 859-958 | AGTTAGAAGACGTATGATGATGCAGAGTGGGGAATCTGATCGGCAATATAAAGGAACCATAGACTGCTTTCTGAAAATCTACCGTCATGAAGGAGTTCCT | 83 | 82 | NM_178386.3:858 |
| Slc25a37 | NM_026331.3 | 2556-2655 | CCATCCTCATGGAGATGACAGGATAGGCTCAGTTTCCTTCTTCCAGTTGTCAACGAGTAAGCTGGGAAGACGCTCATGTTTTCGTGTCCATTGCCCTGGT | 80 | 82 | NM_026331.3:2555 |
| Slc25a4 | NM_007450.4 | 855-954 | CTGCTGGAGGAAGATTGCAAAAGATGAAGGAGCCAACGCTTTCTTCAAAGGTGCTTGGTCCAATGTACTGAGAGGCATGGGTGGTGCTTTTGTATTGGTA | 82 | 81 | NM_007450.4:854 |
| Slc25a5 | NM_007451.3 | 696-795 | AGAATACTCACATCTTCATCAGCTGGATGATTGCACAGTCTGTCACTGCTGTCGCTGGCCTGACTTCCTATCCTTTTGACACGGTTCGCCGTCGTATGAT | 82 | 79 | NM_007451.3:695 |
| Sod1 | NM_011434.1 | 407-506 | TGTGTCCATTGAAGATCGTGTGATCTCACTCTCAGGAGAGCATTCCATCATTGGCCGTACAATGGTGGTCCATGAGAAACAAGATGACTTGGGCAAAGGT | 80 | 81 | NM_011434.1:406 |
| Sod2 | NM_013671.3 | 1496-1595 | ATAGCTTTGCTCCTGCTTGAGGAGTGCTTATTATGAGAGGAAGGTAATGGTGCCTCTGGGTTTTCTATAGGCAGACATGCAAAAACAGCTAATCTTGTCT | 82 | 80 | NM_013671.3:1495 |
| Stard3 | NM_021547.3 | 1393-1492 | TGCACCTTCGTCTGGATTCTTAACACAGATCTTAAGGGCCGCTTGCCTCGGTACCTTATCCACCAGAGCCTTGGGGCCACCATGTTTGAATTTGCCTTTC | 82 | 80 | NM_021547.3:1392 |
| Taz | NM_181516.5 | 27-126 | GACGCTTCCTCGAGTTGCTTTCCGGCTGATTCGTCGAGTGAGGCCGTCAAGACTTGGTTTCTAGTGCGCATTGCTTTGTACAGCAGTCCCGTCTCGTCGC | 81 | 78 | NM_181516.5:26 |
| Timm10 | NM_013899.2 | 17-116 | CCGGAAGTGACCCACGTGCTTCTTATTTCGCCTGCCTTGGTACCCGGTAACTTTTTACTATTGACCCGAAGTTGGGGACGTTGCGGGAGCTGTGACCTGC | 82 | 83 | NM_013899.2:16 |
| Timm10b | NM_019502.3 | 302-401 | AAACTCATCCATTCTAACCACCGCCTCATGGCCGCTTACGTGCACCTCATGCCCGCCCTGGTCCAGCGCCGCATCGCGGACTACGAGGCTGCCTCGGCCG | 84 | 92 | NM_019502.3:301 |
| Timm17a | NM_011590.2 | 347-446 | TAGCCATGGTTGGGTCCGCTGCGATGGGCGGCATTCTCCTAGCTTTAATTGAAGGAGCTGGTATCTTGCTGACAAGATTTGCCTCTGCACAGTTTCCCAA | 81 | 83 | NM_011590.2:346 |
| Timm17b | NM_011591.4 | 375-474 | TGGCCATGGTGGGCTCTGCGATGATGGGAGGCATCCTGTTGGCCCTCATTGAGGGTGTTGGCATCCTTCTCACCCGCTATACTGCCCAGCAGTTCCGAAA | 82 | 81 | NM_011591.4:374 |
| Timm22 | NM_019818.4 | 1617-1716 | GTTCCTGGATGTATTGCAACATGCTGGTAACTCCTCTGCCTCCTCAAAGGCATTGGATTGGCTGATGGTCATGCCTACAGCTTACAAAACCCTTGGTCTT | 82 | 81 | NM_019818.4:1616 |
| Timm23 | NM_016897.3 | 411-510 | TTCGGGGCAATGAACGGTCTTCGTTTAGGATTGAAGGAAACCCAGAGCATGGCCTGGTCCAAACCAAGAAATGTACAGATTTTGAATATGGTGACTAGGC | 82 | 82 | NM_016897.3:410 |
| Timm44 | NM_011592.2 | 877-976 | CTAGGAGGTCTTTTCTCAAAGACAGAAATGTCAGAGGTGCTAACGGAGATCCTGAGAGTGGACCCAACCTTTGACAAGGACCACTTTCTTCATCAGTGTG | 82 | 83 | NM_011592.2:876 |
| Timm50 | NM_025616.3 | 591-690 | TTCACATCAGAGACTGGCATGACTGCATTTCCACTCATTGATAGTGTGGACCCTCACGGTTTCATCTCCTACCGTCTGTTCCGGGACGCCACCAGATATA | 81 | 82 | NM_025616.3:590 |
| Timm8a1 | NM_013898.1 | 736-835 | GTGTCAGTCAAATTGGCAGTGGGGAAAGATCCTGGCACTGTTTAACTGTGAGCCTTCTTTCATGTCTGACCTGGAGCTGAAAAAAAGTCCTATGGAGTGT | 77 | 75 | NM_013898.1:735 |
| Timm8b | NM_013897.2 | 319-418 | GAATGACAGAAGACCAAAAGACTTGTTACCAAGCAGATTGAATGGCCAGTGGTGAAAGACCTGCCAACCTGTCAGGTTAGCGTCAGGCAGTTACAAAGTC | 82 | 82 | NM_013897.2:318 |
| Timm9 | NM_001024853.1 | 377-476 | GAGTTCCTGGGAACCTACAATAAACTTACAGAAACCTGCTTTTTGGACTGTGTTAAAGACTTCACAACAAGAGAGGTGAAACCTGAAGAGGTGACCTGTT | 80 | 81 | NM_001024853.1:376 |
| Tomm20 | NM_024214.2 | 331-430 | AGAAGCTTGCTAAGGAGAGAGCTGGGCTTTCCAAGTTACCTGATTTAAAAGATGCTGAAGCAGTTCAGAAATTCTTCCTTGAAGAGATACAGCTTGGTGA | 82 | 80 | NM_024214.2:330 |
| Tomm22 | NM_172609.3 | 653-752 | TTCCTGTTCTCACTCCAATGCTGGAGTACAGCCATGTGGAAGGTTAGCCTACTCTCTAATAAACTGCTCCTAAGGAAGTTCTTACATGCCCATTGTGGGA | 83 | 81 | NM_172609.3:652 |
| Tomm34 | NM_025996.4 | 649-748 | CCGAAGAAGAAAGTGTTCTGTACTCCAACCGTGCAGCGTGCTACTTGAAGGATGGGAACTGCACAGATTGCATCAAAGATTGCACTTCCGCGCTGGCCTT | 82 | 82 | NM_025996.4:648 |
| Tomm40 | NM_001109748.1 | 1455-1554 | GCCCAGGCAGAGGCGCAGAGGCGGCTGTACACAGGCTCAGAAAGGAAAGACTTGATGTCCTCCTGAGGGCAGCAGAGGAGCGCCGAGCCGCCTGTCACTT | 88 | 89 | NM_001109748.1:1454 |
| Tomm40l | NM_001037170.2 | 371-470 | GCTGCACCGGTTGTGCAAAGATGTATTCCCAGCACAGATGGAAGGCGTGAAGCTGGTTGTCAACAAGGTTCTGAGCAGCCATTTCCAGGTGGCTCATACT | 82 | 82 | NM_001037170.2:370 |
| Tomm70a | NM_138599.5 | 1461-1560 | CTTTGGCGCAGGCTCAGAAGTGTTTTGCATTGTATCGCCAGGCATATACAGCAAACAATTCTTCGCAAGTCCAGGCAGCTATGAAAGGTTTTGAAGAGAT | 79 | 79 | NM_138599.5:1460 |
| Trp53 | NM_011640.1 | 1836-1935 | CCCTCTCTGAGTAGTGGTTCCTGGCCCAAGTTGGGGAATAGGTTGATAGTTGTCAGGTCTCTGCTGGCCCAGCGAAATTCTATCCAGCCAGTTGTTGGAC | 82 | 83 | NM_011640.1:1835 |
| Tspo | NM_009775.4 | 242-341 | GACACTGGCTCCCATCTGGGGCACACTGTATTCAGCCATGGGGTATGGCTCCTACATAGTCTGGAAAGAGCTGGGAGGTTTCACAGAGGACGCTATGGTT | 85 | 85 | NM_009775.4:241 |
| Ucp1 | NM_009463.2 | 739-838 | CTTGTCAACACTTTGGAAAGGGACGACCCCTAATCTAATGAGAAATGTCATCATCAATTGTACAGAGCTGGTAACATATGACCTCATGAAGGGGGCCCTT | 81 | 83 | NM_009463.2:738 |
| Ucp2 | NM_011671.4 | 951-1050 | TGTGCTGAGCTGGTGACCTATGACCTCATCAAAGATACTCTCCTGAAAGCCAACCTCATGACAGATGACCTCCCTTGCCACTTCACTTCTGCCTTCGGGG | 81 | 82 | NM_011671.4:950 |
| Ucp3 | NM_009464.3 | 1026-1125 | CTGCGTCTGGGAGCTTGGAACGTGATGATGTTTGTAACATATGAGCAACTGAAGAGGGCCTTAATGAAAGTCCAGGTACTGCGGGAATCTCCGTTTTGAA | 82 | 83 | NM_009464.3:1025 |
| Uqcr11 | NM_025650.2 | 35-134 | GCAGCGGAACGGGGTGACCCTGAGTATTGAGACCCTGCAGCGATGCTGAGCAGGTTTCTAGGCCCGCGCTACCGGGAACTGGCCAGAAACTGGATTCCCA | 86 | 86 | NM_025650.2:34 |
| Uqcrc1 | NM_025407.2 | 1081-1180 | TTTGCCAGAGTTTCCAGACCTTCAACATCTCCTACTCTGATACTGGGCTGCTGGGCGCACACTTTGTCTGTGATGCCATGAGTATCGATGACATGGTCTT | 82 | 82 | NM_025407.2:1080 |
| Uqcrc2 | NM_025899.2 | 1271-1370 | GGGCTTCCTGAGTGAAATTGGGTCACAGGCTCTAGCTGCAGGTTCTTATATGCCACCTTCTACCGTCCTTCAACAGATTGACTCCGTGGCTGATGCTGAT | 82 | 82 | NM_025899.2:1270 |
| Uqcrfs1 | NM_025710.2 | 437-536 | TGGGTGTTGCTTATGCGGCCAAAAATGTGGTCTCCCAGTTTGTTTCCAGCATGAGTGCTTCTGCTGACGTACTGGCCATGTCGAAGATCGAGATCAAGTT | 80 | 83 | NM_025710.2:436 |
| Uqcrh | NM_025641.3 | 357-456 | AAAAACTTGAAGTAAATGTGCAGATTCGTCCTCCTCAGCCTTGTCACTGGGAATCAGGAACAGTTCCTTGTGGTTCTGGACGTCGGTGTCTGATGGAGTG | 80 | 82 | NM_025641.3:356 |
| Uqcrq | NM_025352.2 | 299-398 | TTTGTAGTGGTCTACCTGATCTACACATGGGGCAACCAGGAGTTTGAGCAGTCGAAAAGGAAGAATCCAGCCATGTATGAAAATGACAAGTAGACGGCCT | 83 | 82 | NM_025352.2:298 |
| Uxt | NM_013840.3 | 467-566 | TGCTAGAGGGACTTAGAGAACTACAAGGCCTGCAGAATTTCCCAGAGCCATCTCCCCATTGACTGCATCTTCCCAGCCTCCAATATTAAAGCACCTGAAT | 89 | 82 | NM_013840.3:466 |
